# DNA Barcoding Unveils New Species of the Sexually Dimorphic Genus *Anteon* Jurine (Hymenoptera, Dryinidae) from China

**DOI:** 10.3390/insects15010018

**Published:** 2023-12-30

**Authors:** Huayan Chen, Massimo Olmi, Frode Ødegaard, Leonardo Capradossi, Jingxian Liu

**Affiliations:** 1 Key Laboratory of Plant Resources Conservation and Sustainable Utilization, South China Botanical Garden, Chinese Academy of Sciences, Guangzhou 510650, China; 2 Tropical Entomology Research Center, Via De Gasperi 10, 01100 Viterbo, Italy; olmi@unitus.it; 3 Department of Natural History, Norwegian University of Science and Technology (NTNU), NO-7491 Trondheim, Norway; frode.odegaard@ntnu.no; 4 Independent Researcher, 01017 Tuscania, Italy; leonardocapradossi.lc@gmail.com; 5 Department of Entomology, South China Agricultural University, Guangzhou 510642, China; liujingxian@scau.edu.cn

**Keywords:** Chrysidoidea, morphological identification, key, molecular species delimitation

## Abstract

**Simple Summary:**

*Anteon* Jurine, 1807 is a cosmopolitan genus of dryinid parasitoids that attack leafhoppers. Sexual dimorphism is extreme in *Anteon* species, rendering the morphological taxonomy of these parasitoids difficult. This paper employs an integrated taxonomic approach that combines morphology with DNA barcoding to investigate the species delimitation of *Anteon* from China. Of the twenty-nine morphospecies examined, twenty-four species were identified as previously known species and five were described here as new species. The sexual association of six species was confirmed by DNA barcoding, indicating that such tools are powerful to tackle the taxonomic challenges in parasitoids with sexual dimorphism.

**Abstract:**

Species of *Anteon* Jurine, 1807 are a large group of parasitoids attacking leafhoppers, which are important insect pests. Despite their great potential in pest biological control, the taxonomy and biology of these parasitoids are far from clear. Sexual dimorphism is extreme in *Anteon* species and has hampered the taxonomy of these parasitoids, resulting in many species described based on a single sex. In this paper, we employed an integrated taxonomic approach for delimitating species, combining morphological examinations with DNA barcoding, to investigate *Anteon* species from China. In total, 53 COI sequences representing 29 species of *Anteon* were obtained and analyzed. On the basis of both morphology and DNA barcoding, five new species of *Anteon* were discovered and described: *A. clariclypeum* sp. nov., *A. maguanense* sp. nov., *A. parafidum* sp. nov., *A. shaanxianum* sp. nov., and *A. shandonganum* sp. nov. The neotype of *A. claricolle* Kieffer is designated. The sexual association of six species was confirmed by DNA barcoding, which led to the synonymy of *Anteon liui* Xu, Olmi & He 2010, new syn., under *Anteon meifenganum* Olmi, 1991. Keys to species of *Anteon* from the Oriental and Eastern Palaearctic are updated to contain the five new species. Our study demonstrates that DNA barcoding is a potent tool for tackling the taxonomic challenges in parasitoids with extreme sexual dimorphism.

## 1. Introduction

The genus *Anteon* Jurine, 1807 (Hymenoptera, Dryinidae) is a large group of parasitoids attacking leafhoppers of the families Cicadellidae and Eurymelidae (Hemiptera, Cicadomorpha) [1]. Since many leafhoppers are important insect pests, these parasitoids are economically important in the biological control of leafhoppers [2]. The genus comprises 465 species distributed worldwide, except Antarctica [3]. In total, 125 species have been reported from China [2], with 116 species belonging to the Oriental region [4,5] and 9 species belonging to the Eastern Palaearctic region [6].

As has been pointed out by many studies, the taxonomy of Dryinidae is challenging due to the extreme sexual dimorphism in these parasitoids, resulting in many species described based on a single sex because the sexual association of the same species is difficult [7,8,9,10]. This situation is especially true in *Anteon*. However, this kind of taxonomic obstacle could be overcome by employing DNA barcoding methods such as the application of the mitochondrial cytochrome c oxidase 1 (*COI*) gene [9,10]. In fact, DNA sequences have been used in the taxonomy, phylogenetics and the confirmation of host associations in Dryinidae and have become an important tool [8,9,11,12,13].

As the second paper of an ongoing research campaign started by the first author (HC), with the objective to collect specimens of dryinids across China and identify the species using an integrated taxonomic approach that combines morphology and DNA barcoding, this study presents the results for the genus *Anteon*.

## 2. Materials and Methods

### 2.1. Insect Specimens and Identification

This study is based upon specimens collected by Malaise traps set up in many provinces of China (See Figure 1 in Olmi et al. [10]). Monthly collected specimens from Malaise traps were kept in 100% ethanol. Dryinidae specimens were sorted out and used for DNA sequencing and morphological examinations. The species of *Anteon* were identified by morphology with the keys provided by Xu et al. [2] and Olmi & Xu [6].

### 2.2. Repositories

The specimens examined in this study are deposited in the following collections, with the abbreviations used in the text: NTNU, Norwegian University of Science and Technology, Department of Natural History, NO-7491 Trondheim, Norway; SCAU, South China Agricultural University, Guangzhou, China; SCBG, South China Botanical Garden, Chinese Academy of Sciences, Guangzhou, China; ZJUC, Zhejiang University, Hangzhou, Zhejiang, China. Regarding the Scandinavian specimens, one mid leg of each individual was torn off and submitted to the University of Guelph in Canada for *COI* barcoding and the generated sequences were uploaded to the BOLD system [14]. All the types of Oriental and Palaearctic species of *Anteon* were examined.

### 2.3. Morphology

The terminology follows Olmi et al. [3]. Abbreviations and morphological terms used in the text are as follows: OL: the distance between inner edges of a lateral ocellus and the median ocellus; OOL: the distance from the outer edge of a lateral ocellus to the eye; OPL: the distance from the posterior edge of a lateral ocellus to the occipital carina; POL: the distance between inner edges of the lateral ocelli; and TL: the distance from the posterior edge of an eye to the occipital carina. Body length was measured from the head to abdominal tip and expressed in millimeters; other measurements are reported in relative values. The terms “metapectal–propodeal disc” and “dorsal surface of the propodeum” are here used in the sense of Kawada et al. [15] and respectively correspond to the “dorsal surface of propodeum” and “posterior surface of propodeum” used by Olmi and Xu [6] and Xu et al. [2].

### 2.4. Imaging

Multifocal images of the new species and representatives of the known species (Figures S1–S25) were made using a mirrorless camera Sony Alpha 6300 (Sony, Tokyo, Japan) with cross table Proxxon KT 70 (Proxxon, Wecker, Luxemburg) or Leica M205C multifocal equipment (Leica, Wetzlar, Germany) and a Nikon SMZ25 microscope mounted with a Nikon DS-Ri 2 digital camera system (Melville, NY, USA). Image plates were made with Adobe Photoshop CS6 Extended.

### 2.5. Sequence Analysis and Molecular Species Delimitation

Genomic DNA of the Chinese specimens was nondestructively extracted from the entire wasp using the TIANamp Micro DNA Kit (Tiangen Biotech, Beijing, China) following the methods used by Taekul et al. [16]. Voucher specimens (Table S1) are deposited in the insect collection of South China Botanical Garden, Chinese Academy of Sciences, Guangzhou, China (SCBG). The *COI* gene was amplified using the LCO1490/HCO2198 primer pair [17]. PCRs, sequencing and sequence analysis were conducted as in the Olmi et al. study [10]. All the newly generated sequences were deposited into GenBank (Table 1), and sequences of six *Anteon* species were downloaded from the BOLD system: *A. claricolle* Kieffer, 1906 (HYMNI1041), *Anteon collare* (Dalman, 1818) (HYMNI945), *Anteon ephippiger* (Dalman, 1818) (HYMNI1151), *Anteon exiguum* (Haupt, 1941) (HYMNI1251), *Anteon fulviventre* (Haliday, 1828) (HYMNI390), *Anteon gaullei* Kieffer, 1905 (HYMNI430). Sequences were aligned by codons using MUSCLE implemented in Geneious 11.0.3. The K2P distances within and between species were calculated in MEGA 11 [18]. A maximum likelihood (ML) tree was generated based on the aligned sequences using the RAxML plugin in Geneious 11.0.3. A sequence of *Aphelopus niger* Xu & He, 1999 (Hymenoptera, Dryinidae) (MZ151323) was downloaded from GenBank and used as an outgroup, according to the phylogenetic topologies present in Tribull [12].

To explore the molecular species delimitation among the studied *Anteon* species, the distance-based barcode gap approach using the Automatic Barcode Gap Discovery (ABGD, Puillandre et al. [19]) and the updated Poisson tree processes model (bPTP, Zhang et al. [20]) were tested. The ABGD method sorts the sequences to hypothetical species by partitioning and comparing the difference between sequences to identify a “barcode gap (a given threshold distance)” (Puillandre et al. [19]). The ABGD analysis was performed on the web interface (https://bioinfo.mnhn.fr/abi/public/abgd/) (accessed on 15 November 2023) using the default priors, Pmin = 0.001, Pmax = 0.1, Steps 10, and with a barcode relative gap width = 1.00, Nb bins (for distance distribution = 20). The bPTP method tests species boundaries on non-ultrametric phylogenetic trees by detecting significant differences in the numbers of substitutions between species and within species (Zhang et al. [20]). For the bPTP analysis, the ML tree generated above was used to perform the analysis on the bPTP web server (https://species.h-its.org/ptp/) (15 November 2023) with default parameters.

## 3. Results

### 3.1. Morphological Identification

In this study, we identified 28 morphospecies based on 53 specimens collected from China and Thailand. Twenty-three species were identified as previously known species and five are described below as new species (Table 1). With the help of DNA barcoding, the sexual association of six species was confirmed, leading to the synonymy of *Anteon liui* Xu, Olmi & He 2010 to *Anteon meifenganum* Olmi, 1991.

### 3.2. Molecular Analysis

**Figure 1:**
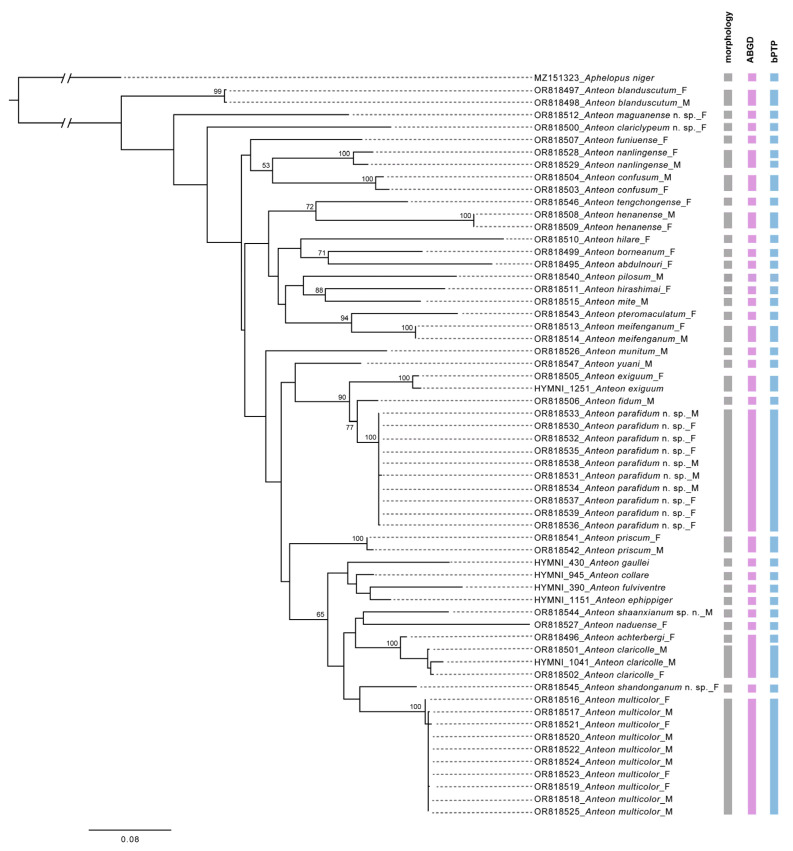
RAxML tree and result of the species delimitation of *Anteon* based on *COI* barcodes. Bootstraps values of 50 and above are indicated. The scale bar represents 0.08 substitutions per site.

The 53 newly generated *COI* sequences in the present study ranged from 576 bp to 674 bp. When analyzed in the BOLD system and GenBank, the sequence of *A. exiguum* (Haupt, 1941) received a close match (around 99%) with multiple sequences labeled as *A. exiguum*, while the sequences of *Anteon claricolle* Kieffer, 1906 received close matches with *A. albidicolle* Kieffer, 1905, which was a synonym of *A. ephippiger* (Dalman, 1818). Other species received no similar sequences in BOLD or GenBank. The intraspecific pairwise distances ranged from 0 to 3% (Table S2). The interspecific pairwise distances ranged from 3.1% to 20.7% (Table S3). The ABGD method produced 31 groups at a priori genetic distance thresholds of 0.002–0.036. *Anteon achterbergi* Olmi, 1991 was grouped together with all three specimens of *A. claricolle*, but genetic distances between these two species ranged from 3.1% to 4.6%. Based on the ML tree, the bPTP method delimited 33 putative species. The two specimens of *Anteon nanlingense* Xu, Olmi & He, 2011, collected from south China and Thailand, respectively, were assigned as putative species. For all the other species, both ABGD and bPTP returned congruent results with the morphological identification, as shown in Figure 1.

### 3.3. Systematics

***Anteon*** Jurine, 1807 [21].

See Xu et al. [2] and Olmi and Xu [6] for taxonomic details on the genus.

### 3.4. Species Descriptions

#### 3.4.1. Anteon Blanduscutum Xu, He & Rui (Figure 2 and Figure 3)

*Anteon blanduscutum* Xu, He & Rui 1996: 214 [22]; He & Xu 2002: 184 [23]; Xu et al. 2011 (Suppl.): 4 [24]; Xu et al. 2013: 78 [2].

**Material examined.** Type: holotype, female: CHINA: Zhejiang, Mt. Tianmushan, 10–12.ix.1983, Xingsheng Wan leg. (ZJUC). Other material: 1 female, CHINA: Fujian, Mt. Longqishan, 11.viii.1991, Changming Liu leg. (ZJUC); 1 female, Fujian, Mt. Longqishan, 8.VII.1991, Changming Liu leg. (ZJUC); 1 female, Hainan, Mt. Yinggeling, 17–20.vii.2010, Huayan Chen leg. (SCAU); 3 females, Hainan, Baisha County, Jiujialing, 17–20.VII.2010, Huayan Chen leg. (SCAU); 2 females, 1 male, Yunnan, Xishuangbanna, Menghai, Bulangshan Village, 1683 m, Area A2, grass, 21°44.981′ N 100°26.907′ E, 15.viii.2018, Li Ma leg., 3040566, 3011677, 3011678 (SCBG); 1 female, Zhejiang, Taishun County, Wuyanling Provincial Nature Reserve, 7.vii–5.viii.2005, (ZJUC); 1 female, Zhejiang, Lin’an, Mt. Qingliangfeng, 9.viii.2005, Min Shi leg. (SCAU).

**Distribution.** China (Zhejiang, Fujian, Hainan, Yunnan).

**Remarks**. *A. blanduscutum* was known only by females [2,22], the male of this species is here confirmed by DNA barcoding. Therefore, we present the following description and diagnosis of the male.

**Figure 2:**
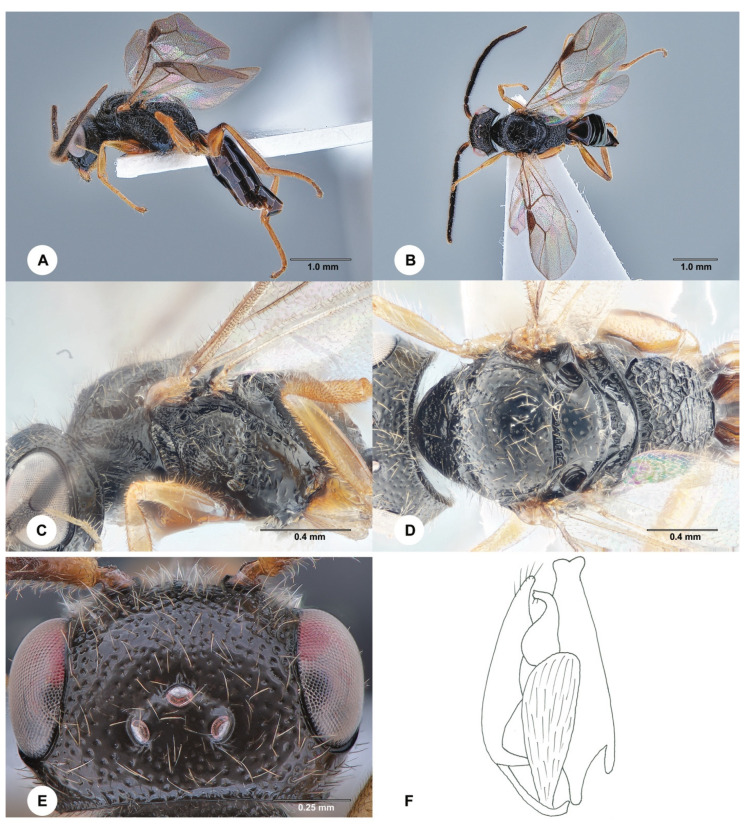
*Anteon blanduscutum* Xu, He & Rui, 1996, male (SCAU 3011678). (**A**) Habitus, lateral view. (**B**) Habitus, dorsal view. (**C**) Head and mesosoma, lateral view. (**D**) Head and mesosoma, dorsal view. (**E**) Head, dorsal view. (**F**) Genitalia (right half removed).

**Description. Male.** Body length 4.1 mm; fully winged (Figure 2A,B). Head black with mandible testaceous; antenna brown, but proximal half of scape testaceous; mesosoma black; metasoma brown; legs testaceous, except the basal extremity of metacoxa is darkened. Antenna filiform; antennomeres in the following proportions: 16:8:14:13:13:13:12:12:11:13. Head (Figure 2E) shiny, punctate, areas between punctures smooth; frontal line absent; frons with two short longitudinal keels near orbits directed towards antennal toruli; OL = 4; OOL = 9; OPL = 8; POL = 8; TL = 8; greatest breadth of lateral ocellus shorter than OL (3:4); occipital carina complete. Mesoscutum and mesoscutellum shiny, punctate, areas between punctures smooth. Notauli incomplete, present at the anterior half of mesoscutum. Metanotum shiny, largely smooth. Metapectal–propodeal complex with strong transverse keel between disc and propodeal declivity; disc reticulate rugose; propodeal declivity rugose, with two complete longitudinal keels. Forewing hyaline; distal part (Rs) of stigmal vein (2r-rs&Rs) more than 0.5, as long as the proximal (2r-rs) part (8:13). Paramere (Figure 2F) about as long as the aedeagus, without a distal inner pointed process. Tibial spurs 1/1/2.

**Diagnosis**. The diagnosis of the male of *A. blanduscutum* is as follows: head black with mandible testaceous; head punctate, areas between punctures smooth; mesoscutum punctate, areas between punctures smooth; notauli present at the anterior half of the mesoscutum; propodeal declivity rugose, with two complete longitudinal keels; distal part of the stigmal vein (2r-rs&Rs) more than 0.5 as long as the proximal part; paramere (Figure 2F) about as long as the aedeagus, without a distal inner pointed process; inner side of the paramere not sculptured by papillae (Figure 2F). Because of the above diagnosis, the male of *A. blanduscutum* can be inserted in the key to the males of the Oriental *Anteon* published by Xu et al. [2] by modifying couplet 57 as follows:

57. Head black, except mandible, clypeus and anterior margin of face testaceous . . . *A. amabile* Xu, Olmi & He.

- Head black, except mandible testaceous. . .57′

57′. Distal part of stigmal vein (2r-rs&Rs) more than 0.5 as long as proximal part (Figure 2B) . . .*A. blanduscutum* Xu, He & Rui

- Distal part of stigmal vein (2r-rs&Rs) less than 0.5 as long as proximal part. . .*A. insertum* Olmi.

#### 3.4.2. *Anteon clariclypeum* Chen, Olmi &, Ødegaard, sp. nov. (Figure 3)

urn:lsid:zoobank.org:act:88025100-DF2A-4058-8943-1A5F843CE18D

**Material examined**. Type: holotype, female: CHINA: Yunnan, Xishuangbanna, Menghai, Bulangshan Village, 21°44.761′ N 100°25.959′ E, 1595 m, Area D, forest, 16.VIII–14.IX.2018, Li Ma leg., MT, 3011671 (SCBG).

**Distribution**. China (Yunnan).

**Etymology**. The species is named *clariclypeum*, because its clypeus is testaceous (from the Latin adjective *clarus* (=clear) + the noun “clypeus”).

**Figure 3:**
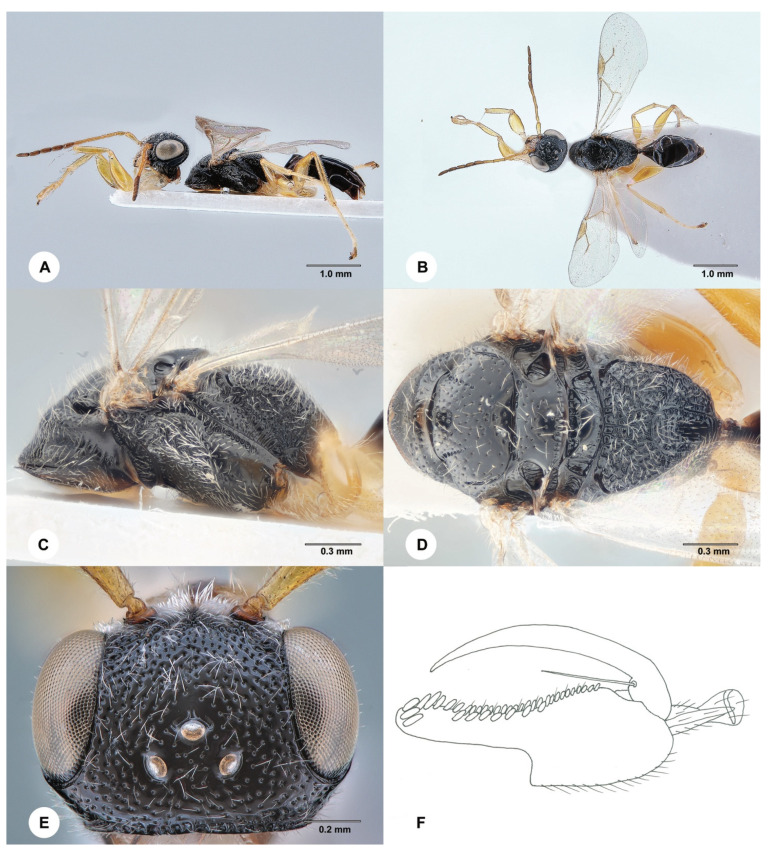
*Anteon clariclypeum* Chen, Olmi & Ødegaard, sp. nov., holotype, female (SCAU 3011671). (**A**) Habitus, lateral view. (**B**) Habitus, dorsal view. (**C**) Mesosoma, lateral view. (**D**) Mesosoma, dorsal view. (**E**) Head, dorsal view. (**F**) Chela.

**Description. Female**. Body length 3.9 mm; fully winged (Figure 3A,B). Head black, except clypeus and mouthparts testaceous; antenna yellow, except antennomeres 6–10 slightly darkened; mesosoma black; metasoma brown; legs yellow. Antenna clavate; antennomeres in the following proportions: 13:6:13:11:10:9:9:9:9:11. Head (Figure 3E) shiny, punctate, areas between punctures smooth; frontal line absent; frons without lateral keels around orbits; occipital carina complete; OL = 3; OOL = 8; OPL = 8; POL = 7; TL = 8; greatest breadth of lateral ocellus shorter than OPL (3:8). Pronotum shiny, punctate, areas between punctures smooth; pronotal tubercle reaching tegula; posterior surface of pronotum much shorter than mesoscutum (6:21). Mesoscutum and mesoscutellum shiny, punctate, areas between punctures smooth. Notauli incomplete, present at the anterior 0.6 of scutum (Figure 3D). Metanotum shiny, unsculptured. Mesopleuron shiny, punctate, areas between punctures smooth, except the proximal half is dull and rugose. Metapleuron transversely striate and rugose. Metapectal–propodeal complex with transverse keel between disc and propodeal declivity; disc reticulate rugose; propodeal declivity with two longitudinal keels, median area almost completely shiny and unsculptured and lateral areas reticulate rugose. Forewing hyaline, without dark transverse bands; distal part (Rs) of stigmal vein (2r-rs&Rs) shorter than proximal (2r-rs) part (8:13). Protarsomeres in following proportions: 11:3:3:7:19. Protarsomere 2 produced into hook. Enlarged claw (Figure 3F) with proximal prominence bearing one long bristle. Protarsomere 5 (Figure 3F) with the basal region about as long as the distal region, with two rows of about 38 lamellae without interruption to distal apex. Tibial spurs 1/1/2.

**Male**. Unknown.

**Diagnosis**. Female with head (Figure 3E) punctate and unsculptured among the punctures; clypeus testaceous; frons without lateral keels along orbits; OL shorter than POL, OPL more than twice as long as the greatest breadth of lateral ocellus; mesosoma totally black; propodeal declivity with two longitudinal keels and median area almost completely unsculptured; protarsomere 4 shorter than 1; basal region of the protarsomere 5 (Figure 3F) about as long as the distal region. *A. clariclypeum* sp. nov. is similar to *A. kresli* Olmi, 2008. However, in *A. clariclypeum*, the clypeus is testaceous; the frons has no lateral keels along the orbits directed towards the antennal toruli; protarsomere 4 is shorter than 1; the basal region of the protarsomere 5 (Figure 3F) is about as long as the distal region (in *A. kresli*, the clypeus is black; the frons has two lateral keels along the orbits directed towards antennal toruli; the protarsomere 4 is longer than 1; the basal region of the protarsomere 5 is much shorter than the distal region (figure 32A in Xu et al. 2013) [2]). Following the description of *A, clariclypeum*, the key to the females of the Oriental species of *Anteon* published by Xu et al. (2013) [2] should be modified by replacing couplet 67 as follows:

67. Propodeal declivity with median region shiny, punctate and areas between punctures smooth. . .67′

- Propodeal declivity with median region dull and rugose. . .68

67′. Clypeus black; frons with two lateral keels along orbits directed towards antennal toruli; protarsomere 4 longer than 1; basal region of protarsomere 5 much shorter than the distal region (figure 32A in Xu et al. [2]) . . .*A. kresli* Olmi

- Clypeus testaceous; frons without two lateral keels along orbits directed towards antennal toruli (Figure 3E); protarsomere 4 shorter than 1; basal region of protarsomere 5 about as long as the distal region (Figure 3F) . . .*A. clariclypeum* sp. nov.

#### 3.4.3. *Anteon claricolle* Kieffer (Figure 4 and Figure 5)

*Anteon claricollis* Kieffer, in Kieffer & Marshall, 1906: 514 [25]; Richards, 1939: 268 [26].

**Material examined**. Type: female, neotype (here designated): NORWAY: Oslo, Gaustad “Jubileumsenga”, 59.94841764° N, 10.711697° E, 110 m, 29.VI–1.VIII.2014, MT, K.M. Olsen leg. (NTNU). Other material: 1 male, NORWAY: EIS 12, Østfold, Hvaler, Søndre Sandøy, Ødegårdstranda, 59.00221° N, 11.07474° E, 8.V–15.VII.2014, Frode Ødegaard leg. (NTNU); 1 male, Østfold, Hvaler, Søndre Sandøy, 59.002° N, 11.075° E, 15.VII.2014, Frode Ødegaard leg. (NTNU); 1 male, CHINA: Inner Mongolia, Wulan Aodu Experimental Station, North, 38°46′15″ N, 108°46′41″ E, 20–30.VI.2013, MT, Yongming Luo leg., SCAU 3011712 (SCBG); 2 males, Ningxia Hui Nationality Autonomous Region, Mt. Liupanshan, 35°29′12″ N, 106°20′29″ E, 3–14.VII.2009, Huayan Chen leg., 3040792, 3040793 (SCBG); 1 male, Shandong, Linyi, Lanling County, MT, 34°51′ N, 118°4′ E, 25.VIII–8.IX.2014, Xuejun Yang leg., SCAU 3040677 (SCBG); 1 male, Sichuan, Chengdu, Longquanyi District, Baihe Town, Changsong Village, 30°31′17″ N, 104°17′0″ E, 8.X.2012, MT, SCAU 3019637 (SCBG).

**Distribution**. Norway, China (Inner Mongolia, Ningxia, Shandong, Sichuan).

**Remarks**. *Anteon claricolle* Kieffer, 1906 is a species ignored in all treatments of Palaearctic *Anteon*, including the main revisions led by Olmi [6,27]. The main reason for this omission is that the type was considered lost (see also Richards [26]) and the original description is unreliable. Kieffer examined six females of this species, all deposited in Marshall’s collection [25]. In the labels, the localities of capture were indicated by the following abbreviations: Bugsby, Bfm., B.T. and N. (all localities apparently situated in England). However, these specimens are not deposited in the main European collections, including the Museum of Natural History of Paris (France) and the Natural History Museum of London (UK). Richards [26] wrote that the types were lost and considered *A. claricolle* as a junior synonym of *A. ephippiger* (Dalman, 1818) var. *collaris* (Dalman, 1818). In recent years, one of the authors (Frode Ødegaard) collected some female specimens with a pale yellow prothorax, whose morphology and color corresponded to the description of *A. claricolle*. COI sequences of these specimens supported that they were different from specimens of *A. ephippiger* var. *collaris* (i.e., females of *A. ephippiger* with head and mesosoma black, except that the prothorax is ferruginous or reddish). In addition, Frode Ødegaard also collected males of *Anteon* with COI sequences identical to those of the females of *A. claricolle* and different from the COI sequences of males of *A. ephippiger* var. *collaris*. Therefore, we decided to resurrect *A. claricolle* by designating a female neotype (see the description below).

**Figure 4:**
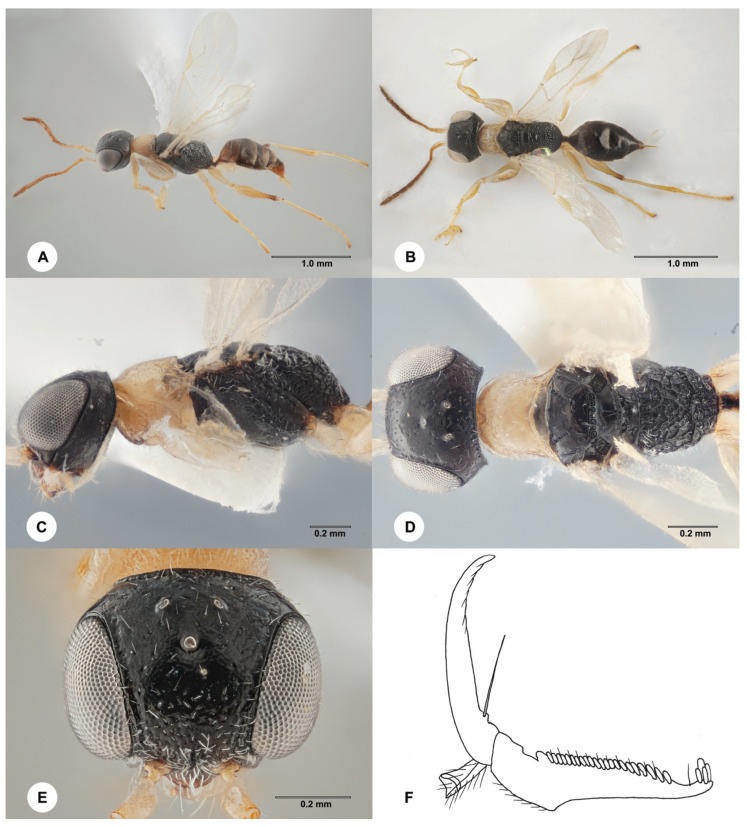
*Anteon claricolle* Kieffer, 1906, neotype, female. (**A**) Habitus, lateral view. (**B**) Habitus, dorsal view. (**C**) Head and mesosoma, lateral view. (**D**) Head and mesosoma, dorsal view. (**E**) Head and anterior mesosoma, dorsal view. (**F**) Chela.

**Description. Female, neotype**. Body length 2.4 mm; fully winged (Figure 4). Head black, except mandible and clypeus are testaceous; antenna yellow; mesosoma black, except prothorax is pale yellow (Figure 4A–D); metasoma brown; legs yellow. Antenna clavate; antennomeres in following proportions: 8:5:5:5:5:4:4:4:5:6. Head (Figure 4E) shiny, finely punctate, areas between punctures smooth; frontal line absent; frons with two lateral keels along orbits directed towards antennal toruli; occipital carina complete; OL = 3; OOL = 5; OPL = 4; POL = 4; TL = 4; greatest breadth of lateral ocellus shorter than OPL (2:4). Pronotum shiny, finely punctate, areas between punctures smooth, with the posterior surface longer than mesoscutum (11:7); pronotal tubercle reaching tegula. Mesoscutum, mesoscutellum and metanotum shiny, punctate, areas between punctures smooth. Notauli incomplete, present at the anterior 0.6 of the mesoscutum (Figure 4D). Metapectal–propodeal complex with strong transverse keel between disc and propodeal declivity; disc reticulate rugose; propodeal declivity rugose, with two longitudinal keels. Forewing hyaline; distal part (Rs) of the stigmal vein (2r-rs&Rs) much shorter than the proximal (2r-rs) part (3:8). Protarsomeres in the following proportions: 5:2:2:7:14. Protarsomere 3 produced into a hook. Enlarged claw (Figure 4F) with a proximal prominence bearing one long bristle. Protarsomere 5 (Figure 4F) with one row of about 22 lamellae; distal apex with a group of about four lamellae. Tibial spurs 1/1/2.

**Figure 5:**
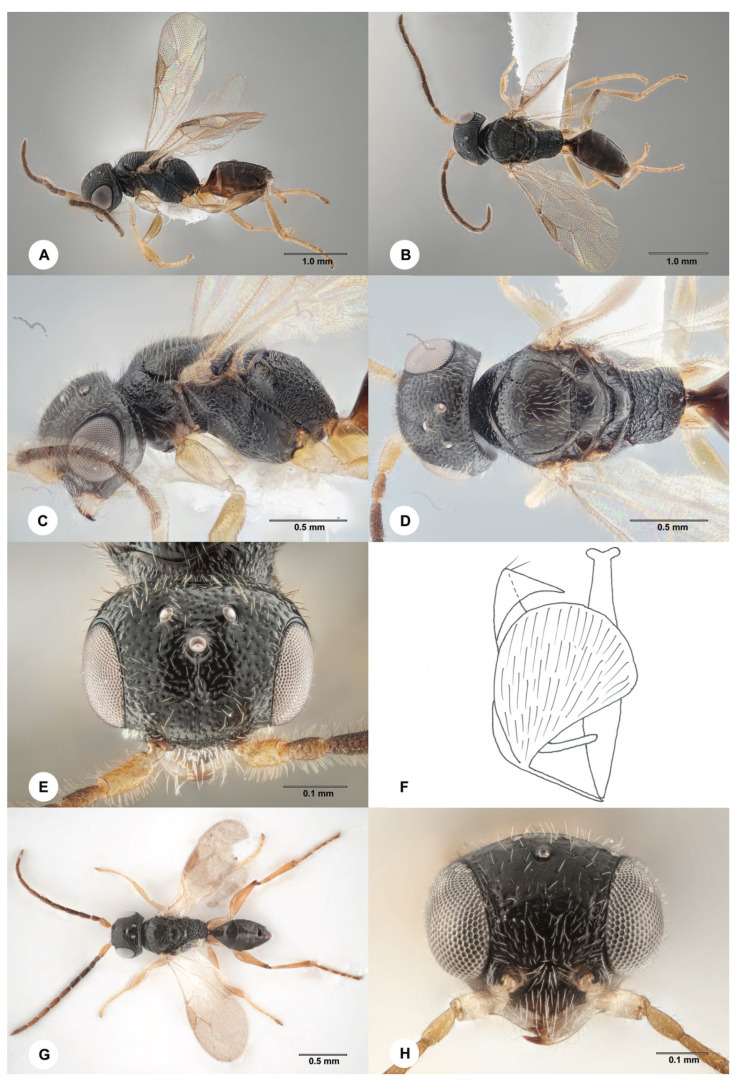
*Anteon claricolle* Kieffer, 1906, male, (**A**–**F**): SCAU 3011712) (**A**) Habitus, lateral view; (**B**) habitus, dorsal view; (**C**) head and mesosoma, lateral view; (**D**) head and mesosoma, dorsal view; (**E**) head and anterior mesosoma, dorsal view; (**F**) genitalia (right half removed); (**G**,**H**): from Norway) (**G**) habitus, dorsal view; (**H**) head, anterior view.

**Description. Male from China**. Body length 3.1 mm; fully winged (Figure 5A,B). Head black, except mandible testaceous and clypeus ferruginous; antenna brown, except scape yellow; mesosoma black; metasoma brown; legs yellow. Antenna filiform; antennomeres in the following proportions: 8:5:6.5:6:6:6:6:6:6:9. Head (Figure 5E) shiny, punctate, areas between punctures smooth; frontal line absent; frons with median longitudinal furrow; OL = 3; OOL = 6; OPL = 4; POL = 5TL = 5; greatest breadth of lateral ocellus shorter than OL (2:3); occipital carina complete. Mesoscutum shiny, punctate, areas between punctures smooth. Notauli incomplete, reaching approximately 0.5 × the length of the mesoscutum (Figure 5D). Scutellum and metanotum shiny, smooth. Metapectal–propodeal complex with strong transverse keel between disc and propodeal declivity; disc reticulate rugose; propodeal declivity rugose, with two complete longitudinal keels (Figure 5D). Forewing hyaline; distal part (Rs) of the stigmal vein (2r-rs&Rs) shorter than the proximal (2r-rs) part (2.5:8). Paramere (Figure 5F) with a distal inner pointed process provided of inner distal margin not excavated; dorsal membranous band very large (Figure 5F). Tibial spurs 1/1/2.

**Description. Male from Norway**. Body length 1.7 mm; fully winged (Figure 5G). Head black with mandible testaceous and clypeus ferruginous; antenna brown, except scape testaceous and pedicel brown-testaceous; mesosoma black; metasoma brown; legs yellow, except metacoxa proximally brown and distal extremity of metafemur brown. Antenna filiform; antennomeres in the following proportions: 8:5:6:5:7:7:7:7:6:9. Head (Figure 5H) shiny, finely punctate, areas between punctures smooth; frontal line incomplete, absent in anterior third of frons; frons without lateral keels around orbits directed towards antennal toruli; OL = 2; OOL = 5; OPL = 3; POL = 4; TL = 5; greatest breadth of lateral ocellus about as long as OL; occipital carina complete. Mesoscutum shiny, finely punctate, areas between punctures smooth, strongly punctate near anterior margin. Notauli incomplete, present at the anterior 0.6 of the mesoscutum (Figure 5G). Mesoscutellum unsculptured. Metanotum rugose. Metapectal–propodeal complex with strong transverse keel between disc and propodeal declivity; disc reticulate rugose; propodeal declivity rugose, with two longitudinal keels. Forewing hyaline, without dark transverse bands; distal part (Rs) of the stigmal vein (2r-rs&Rs) much shorter than the proximal (2r-rs) part. Genitalia with a distal inner pointed process and dorsal membranous process (shape like that shown in Figure 5F). Tibial spurs 1/1/2.

**Diagnosis**. Female (Figure 4A–D) with head black, finely punctate, areas between punctures smooth; mesosoma black, except prothorax pale yellow (Figure 4C,D); mesoscutum punctate, areas between punctures smooth (Figure 4D); notauli incomplete, present at the anterior 0.6 of the mesoscutum (Figure 4D); propodeal declivity rugose, with two longitudinal keels (Figure 4D); forewing hyaline (Figure 4A); protarsomere 5 (Figure 4F) with one row of about 22 lamellae. Male (Figure 5) with head black, except mandible testaceous and occasionally clypeus ferruginous; head (Figure 5E,H) punctate, areas between punctures smooth; propodeal declivity provided with two longitudinal keels and median area as rugose as lateral regions (Figure 5D); genitalia with a very large dorsal membranous band (Figure 5F); paramere (Figure 5F) with a very long distal inner pointed process extended apically, provided of inner distal margin not excavated; legs yellow (Figure 5A,G).

The female of *A. claricolle* can be included in the key to the *Anteon* females of Fennoscandia and Denmark [7] by replacing couplet 12 as follows:

12. Head dull, always clearly and strongly granulated; antennomere 1 approximately twice as long as 4. . . *A. fulviventre* (Haliday)

- Head shining, punctate, without sculpture among punctures (Figure 4E) or very weakly granulated; antennomere 1 approximately as long as or slightly longer than 4. . .13

13. Notauli incomplete, reaching about 0.3–0.5 length of the mesoscutum; mesosoma variably colored, occasionally black, except prothorax reddish or testaceous (never pale yellow); head variably colored, if black, mandible testaceous and clypeus black. . . *A. ephippiger* (Dalman)

- Notauli incomplete, reaching about 0.60 length of the mesoscutum; prothorax pale yellow (rest of mesosoma black); head black, except mandible and clypeus testaceous. . .*A. claricolle* Kieffer

The male of *A. claricolle* can be included in the key to the *Anteon* males of Fennoscandia and Denmark [7] by replacing couplet 11 as follows:

11. Paramere with dorsal membranous band very short (Plate 11H in Olmi and Xu [6]); head in part slightly granulated, in part rugose, in part punctate, occasionally alutaceous, with sculpture usually slightly distinct. . . *A. exiguum* (Haupt)

- Paramere with very long dorsal membranous band (figure 5F; plates 11C, 12E, 15H in Olmi and Xu [6]); head punctate, areas between punctures smooth; rarely slightly granulated among punctures; head surface never alutaceous. . . 11′

11′. Distal inner process of paramere very long, with inner margin not or slightly excavated (Figure 5F) . . . *A. claricolle* Kieffer

- Distal inner process of paramere shorter and with inner margin not excavated (figures 11C and 12E in Olmi and Xu [6]) . . . 12

12. Head more strongly punctate, areas between punctures smooth or very slightly granulated, with a short or long frontal line. . . *A. gaullei* Kieffer

- Head finely punctate, smooth, areas between punctures smooth or very slightly granulated, usually without frontal line. . . *A. ephippiger* (Dalman)

The female of *A. claricolle* can be included in the key to the *Anteon* females of the Eastern Palaearctic region [6] by replacing couplet 50 as follows:

50. Notauli incomplete, reaching about 0.3–0.5 length of the mesoscutum. . . *A. ephippiger* (Dalman)

- Notauli incomplete, reaching about 0.60–0.80 length of the mesoscutum. . . 51

51. Mesosoma completely testaceous, except disc of the metapectal–propodeal complex brown. . . *A. shandonganum* sp. Nov.

- Mesosoma totally black; occasionally black, except prothorax pale yellow (Figure 4D) . . . 52

52 Clypeus testaceous (Figure 4C); prothorax pale yellow (Figure 4D) . . . *A. claricolle* Kieffer

- Clypeus and prothorax black. . . *A. munitum* Olmi

The male of *A. claricolle* can be included in the key to the males of the *Anteon* males of the Eastern Palaearctic published by Olmi and Xu [6] by replacing couplet 38 as follows:

38. Propodeal declivity with median area almost completely smooth, not rugose. . . *A. sulawesianum* Olmi

- Propodeal declivity with median area rugose. . . 38′

38′. Distal inner process of paramere very long (figure 5F; Plate 26C in Xu et al. [2]) . . . 38′’.

- Distal inner process of paramere shorter (Plates 11C, 12E in Olmi and Xu [6]) . . . 39

38′’. Distal inner process of paramere with inner margin slightly excavated (Plate 26C in Xu et al. [2]) . . . *A. fidum* Olmi

- Distal inner process of paramere with inner margin not excavated (Figure 5F) . . . *A. claricolle* Kieffer

#### 3.4.4. *Anteon maguanense* Chen, Olmi & Liu, sp. nov. (Figure 6)

urn:lsid:zoobank.org:act:1E2871CD-57E1-4343-A46B-A514F2EC62B7

**Material examined. Type**: holotype, female: CHINA: Yunnan, Muchang County, Maguan Town, coniferous forest, 22.91888° N, 104.162851° E, 1336 m, MT, VI.2017, Li Ma leg., 3011738 (SCBG).

**Distribution.** China (Yunnan).

**Etymology**. The species is named after Maguan Town, the main town near the capture locality.

**Figure 6:**
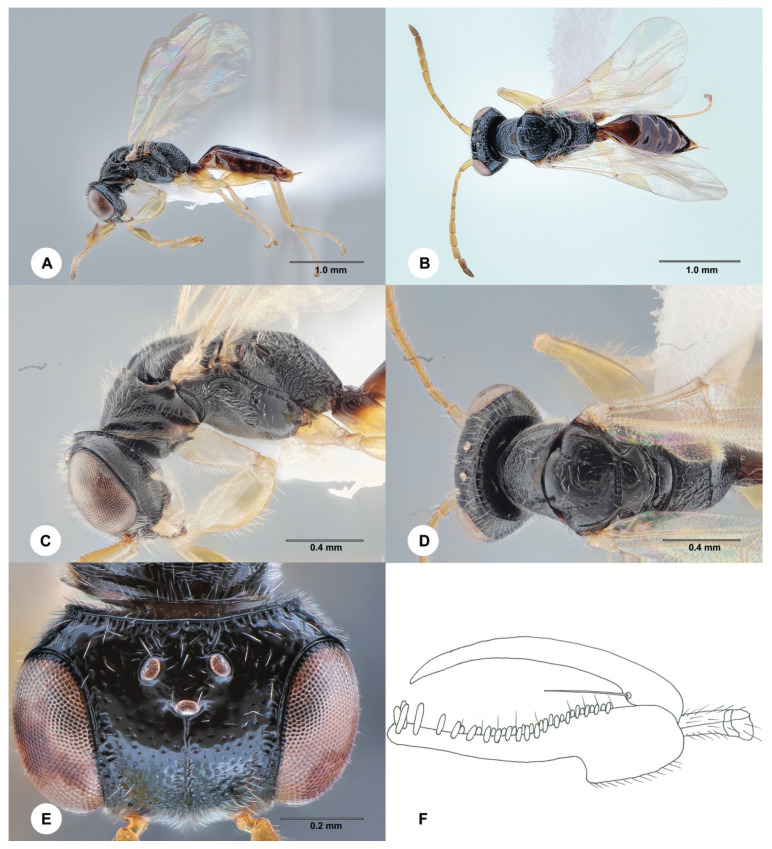
*Anteon maguanense* Chen, Olmi & Liu, sp. nov., holotype, female (SCAU 3011738). (**A**) Habitus, lateral view. (**B**) Habitus, dorsal view. (**C**) Head and mesosoma, lateral view. (**D**) Head and mesosoma, dorsal view. (**E**) Head, dorsal view. (**F**) Chela.

**Description. Female, holotype.** Body length 2.8 mm; fully winged (Figure 6A,B). Head black with mandible testaceous; antenna testaceous; mesosoma black; metasoma brown; legs yellow with proximal extremity of metacoxa black. Antenna clavate; antennomeres in the following proportions: 11:5:7:7:6:6:6:6:5:8. Head (Figure 6E) shiny, punctate, areas between punctures smooth, except anterior third of frons rugose; vertex without two oblique keels connecting lateral ocelli to occipital carina; frontal line complete; frons with two lateral longitudinal keels along orbits directed towards antennal toruli; OL = 3; OOL = 5; OPL = 3; POL = 4; TL = 5; greatest breadth of lateral ocelli as long as OPL; occipital carina complete. Pronotum shiny, with the anterior surface about as long as the posterior surface; posterior surface shiny, punctate, areas between punctures smooth, much shorter than mesoscutum (6:16); pronotal tubercle reaching tegula. Mesoscutum and mesoscutellum shiny, punctate, areas between punctures smooth. Notauli incomplete, present at the anterior 0.7 of the mesoscutum (Figure 6D). Metanotum shiny, unsculptured. Metapectal–propodeal complex with strong transverse keel between disc and propodeal declivity; disc reticulate rugose; propodeal declivity rugose, with two complete longitudinal keels. Forewing hyaline; distal part (Rs) of the stigmal vein (2r-rs&Rs) shorter than the proximal (2r-rs) part (6:10). Protarsomeres in the following proportions: 6:3:3:9.5:19. Enlarged claw (Figure 6F) with proximal prominence bearing one long bristle. Protarsomere 5 (Figure 6F) straight before distal apex, with two rows of about 29 lamellae; distal apex with four lamellae. Tibial spurs 1/1/2.

**Male**. Unknown.

**Diagnosis**. Female with face partly reticulate rugose and partly punctate, areas between punctures smooth; head vertex without oblique keels connecting lateral ocelli to occipital carina; posterior surface of the pronotum about as long as the anterior surface, about 0.5 as long as the mesoscutum; forewing hyaline without dark transverse bands; propodeal declivity with two longitudinal keels and median area as rugose as lateral areas; protarsomere 4 longer than segment 1; protarsomere 5 with the basal part much shorter than the distal part; protarsomere 5 is straight before the distal apex (Figure 6F). *A. maguanense* sp. nov. is similar to *A. semirugosum* Xu, Olmi, Guglielmino & Chen, 2012. However, in the new species, the head vertex has no oblique keels connecting lateral ocelli to occipital carina and the protarsomere 5 is straight before the distal apex (Figure 6F) (in *A. semirugosum*, head vertex with two oblique keels connecting lateral ocelli to occipital carina and protarsomere 5 curved before the distal apex (Plate 44A in Xu et al. [2]). Following the description of *A. maguanense*, the key to the females of the Oriental species of *Anteon* published by Xu et al. [2] should be modified by replacing couplet 98 as follows:

98. Face completely punctate, areas between punctures smooth; posterior surface of pronotum about as long as mesoscutum. . . *A. wangi* Xu, He & Olmi

- Face partly rugose, with the rest of the surface punctate, areas between punctures smooth; posterior surface of pronotum about 0.5 as long as mesoscutum. . . 98′

98′. Vertex of head with two oblique keels connecting lateral ocelli to occipital carina; protarsomere 5 curved before distal apex (figure 44A in Xu et al. [2]) . . . *A. semirugosum* Xu, Olmi & Guglielmino

- Vertex of head without oblique keels connecting lateral ocelli to occipital carina; protarsomere 5 straight before distal apex (Figure 6F) . . . *A. maguanense* sp. nov.

#### 3.4.5. *Anteon meifenganum* Olmi (Figure 7 and Figure 8)

*Anteon meifenganum* Olmi 1991: 173 [28]; Xu et al. 2013: 135 [2].

*Anteon liui* Xu, Olmi & He 2010: 404 [29] (**new syn.**).

**Material examined**: 1 male, CHINA: Shaanxi, Yang County, Huayang Town, 33.675537° N, 107.349632° E, 922 m, 12.IV–12.V.2017, MT, Haoyu Liu leg., SCAU 3040520 (SCBG); 1 female, Shaanxi, Yang County, Huayang Town, 33.382156° N, 107.507905° E, 1154 m, 26.V–26.VI.2017, MT, Haoyu Liu leg., SCAU 3040514 (SCBG).

**Distribution**. China (Taiwan, Guizhou, Hunan, Shaanxi, Zhejiang), Myanmar, Thailand (Xu et al. 2013).

**Remarks**. *Anteon meifenganum* Olmi, 1991 and *A. liui* Xu, Olmi & He 2010 were described, respectively, by males [28] and females [6]. The *COI* sequences of the new material of these two species generated in this study are identical, indicating that they are just the opposite sex of the same species. Therefore, *A. liui* is here treated as a junior synonym of *A. meifenganum*.

**Figure 7:**
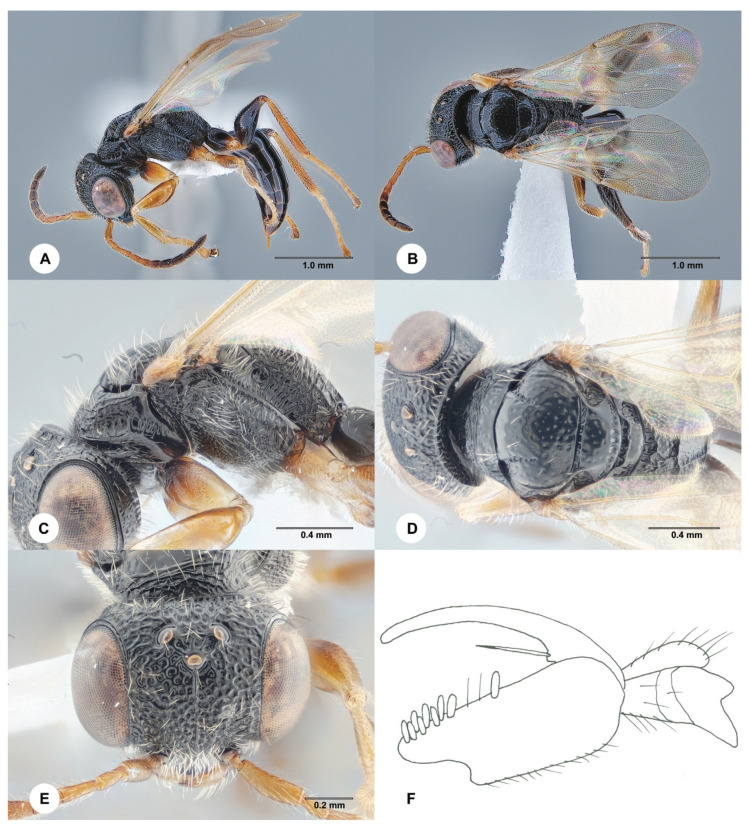
*Anteon meifenganum* Olmi, 1991, female (SCAU 3040514). (**A**) Habitus, lateral view. (**B**) Habitus, dorsal view. (**C**) Head and mesosoma, lateral view. (**D**) Head and mesosoma, dorsal view. (**E**) Head, dorsal view. (**F**) Chela.

**Figure 8:**
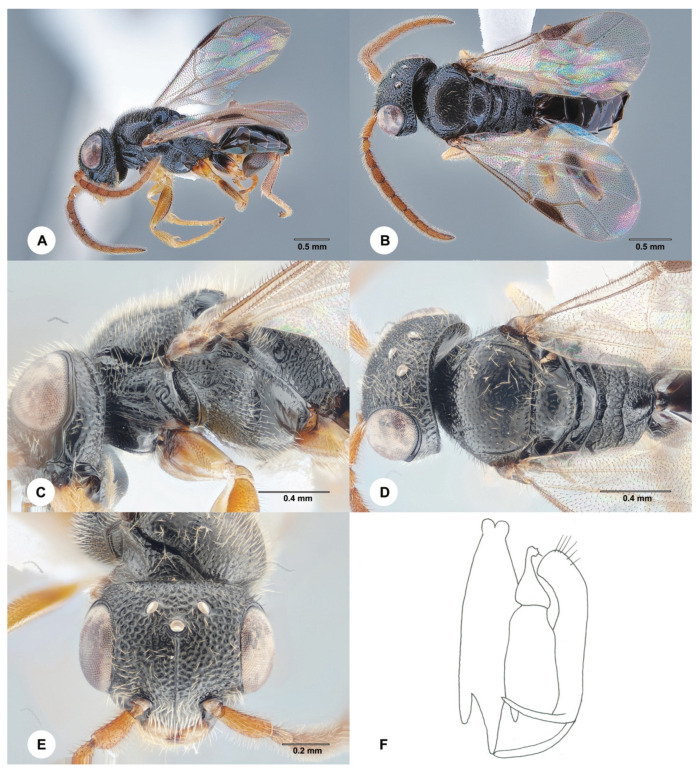
*Anteon meifenganum* Olmi, 1991, male (SCAU 3040520). (**A**) Habitus, lateral view. (**B**) Habitus, dorsal view. (**C**) Head and mesosoma, lateral view. (**D**) Head and mesosoma, dorsal view. (**E**) Head, anterior view. (**F**) Genitalia (left half removed).

#### 3.4.6. *Anteon parafidum* Chen, Olmi & Ødegaard, sp. nov. (Figure 9 and Figure 10)

urn:lsid:zoobank.org:act:06445F95-8DC6-4E6E-B027-695946992551

**Material examined. Types**: female, holotype: CHINA: Yunnan, Xishuangbanna, Menghai, Bulangshan Village, 1683 m, Area A, grass, 21°45.037′ N, 100°26.715′ E, 17.V–21.VI.2018, MT, Li Ma leg., SCAU 3011658 (SCBG). Paratypes: 1 male, same locality label as holotype, SCAU 3011662 (SCBG); 1 female, 1 male, Yunnan, Xishuangbanna, Menghai, Bulangshan Village, 1683 m, Area A2, grass, 21°44.981′ N, 100°26.907′ E, 15.VIII.2018, MT, Li Ma leg., SCAU 3040566, SCAU 3040567 (SCBG); 1 female, 1 male, same locality as holotype, but 21.VIII–20.IX.2019, SCAU 3044018, SCAU 3044026 (SCBG); 1 female, 1 male, same locality as holotype, but 20.VII–21.VIII.2019, SCAU 3044089, SCAU 3044088 (SCBG); 1 male, same locality as holotype, but 28.V–28.VI.2019, SCAU 3044066 (SCBG); 1 female, Yunnan, Xishuangbanna, Menghai, Bulangshan Village, 1683 m, Area A2, grass, 21°44.981′ N, 100°26.907′ E, 20.IV–28.V.2019, MT, Li Ma leg., 3044033 (SCBG).

**Distribution.** China (Yunnan).

**Etymology**. The species is named *parafidum*, because it is morphologically similar to *Anteon fidum* Olmi, 1991 (from the Greek prefix para- (meaning “close to”) and *fidum*).

**Figure 9:**
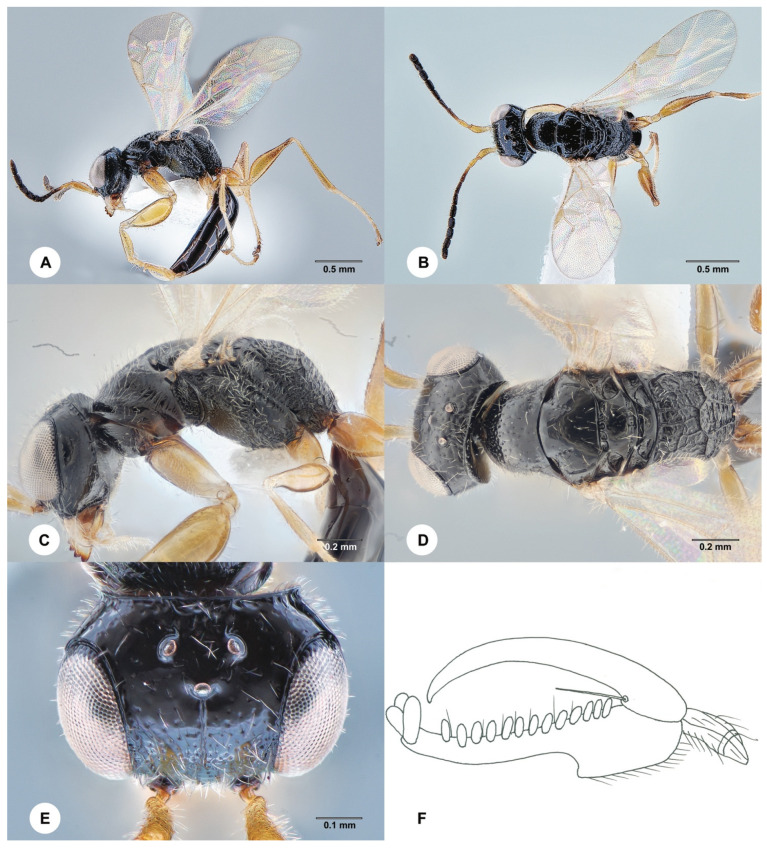
*Anteon parafidum* Chen, Olmi & Ødegaard, sp. nov., holotype, female (SCAU 3011658). (**A**) Habitus, lateral view. (**B**) Habitus, dorsal view. (**C**) Head and mesosoma, lateral view. (**D**) Head and mesosoma, dorsal view. (**E**) Head, dorsal view. (**F**) Chela.

**Description. Female, holotype.** Body length 2.6 mm; fully winged (Figure 9A,B). Head black with mandible testaceous; antenna brown with scape and pedicel testaceous; mesosoma black; metasoma brown; legs testaceous. Antenna clavate; antennomeres in the following proportions: 11:5:7:6:5:5:5:5:5:7. Head (Figure 9E) shiny, punctate, areas between punctures smooth; frontal line complete; frons with two lateral longitudinal keels along orbits directed towards antennal toruli; occipital carina complete; OL = 2; OOL = 5; OPL = 4; POL = 5; TL = 4; greatest breadth of lateral ocellus as long as OL. Pronotum shiny, punctate, areas between punctures smooth, with the posterior surface shorter than the mesoscutum (9:12); pronotal tubercle reaching tegula. Mesoscutum shiny, punctate, areas between punctures smooth. Notauli incomplete, present at the anterior 0.5 of the mesoscutum (Figure 9D). Mesoscutellum and metanotum shiny, unsculptured. Metapectal–propodeal complex with strong transverse keel between disc and propodeal declivity; disc reticulate rugose; propodeal declivity rugose, with two complete longitudinal keels. Forewing hyaline; distal part (Rs) of the stigmal vein (2r-rs&Rs) much shorter than the proximal (2r-rs) part (2:10). Protarsomeres in the following proportions: 6:2:2.5:7:16. Enlarged claw (Figure 9F) with proximal prominence bearing one long bristle. Protarsomere 5 (Figure 9F) with the basal part slightly shorter than the distal part, with one row of 13 large lamellae; distal apex with three large lamellae. Tibial spurs 1/1/2.

**Figure 10:**
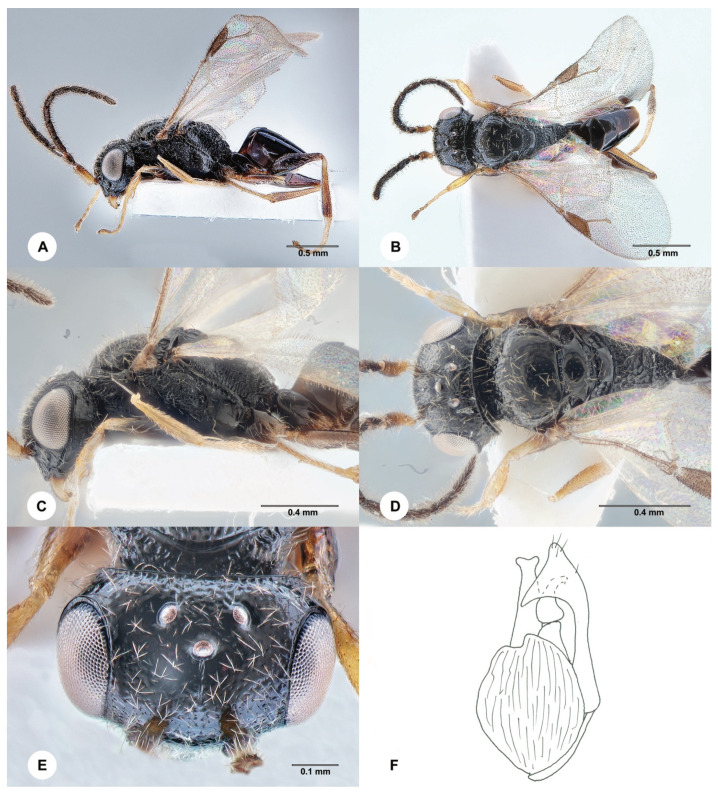
*Anteon parafidum* Chen, Olmi & Ødegaard, sp. nov., paratype, male (SCAU 3011662). (**A**) Habitus, lateral view. (**B**) Habitus, dorsal view. (**C**) Head and mesosoma, lateral view. (**D**) Head and mesosoma, dorsal view. (**E**) Head, anterior view. (**F**) Genitalia (left half removed).

**Description of Male.** Fully winged (Figure 10A,B); length 2.1 mm. Head black with mandible testaceous; antenna brown with ventral side of scape testaceous; mesosoma black; metasoma brown; legs brown with tarsi and protibia testaceous. Antenna filiform; antennomeres in the following proportions: 10:6:7:7:7:7:7:7:7:9. Head (Figure 10E) shiny, punctate, areas between punctures smooth; OL = 3; OOL = 5; OPL = 3; POL = 5; TL = 3; greatest breadth of lateral ocellus shorter than OPL (2:3); occipital carina complete; frontal line absent; frons without lateral longitudinal keels along orbits. Mesoscutum punctate, areas between punctures smooth. Notauli incomplete, present at the anterior 0.5 of the scutum (Figure 10D). Mesoscutellum and metanotum shiny, unsculptured. Metapectal–propodeal complex with strong transverse keel between disc and propodeal declivity; disc reticulate rugose; propodeal declivity rugose, with two longitudinal keels. Forewing hyaline; distal part (Rs) of the stigmal vein (2r-rs&Rs) much shorter than the proximal (2r-rs) part (2.5:8). Paramere (Figure 10F) with distal inner pointed process; inner distal margin of pointed process slightly excavated. Dorsal process of paramere shorter than volsella (Figure 10F). Tibial spurs 1/1/2.

**Diagnosis**. Female (Figure 10E) with head punctate and areas between punctures smooth; OL shorter than POL; OPL twice as long as the greatest breadth of lateral ocellus; mesosoma black; propodeal declivity with two complete longitudinal keels and median region as rugose as lateral regions; protarsomere 4 slightly longer than 1; protarsomere 5 provided with very large lamellae, with the basal part shorter than the distal part (Figure 10F). Male (Figure 10E) with head punctate and areas between punctures smooth; propodeal declivity with two complete longitudinal keels and median region as rugose as lateral regions; pointed distal inner process of paramere with inner distal margin slightly excavated; paramere with dorsal process shorter than volsella (Figure 10F).

The female of *A. parafidum* is very similar to *A. fidum* Olmi, 1991. However, in *A. parafidum*, protarsomere 5 has its basal part shorter than the distal part (Figure 9F) (in *A. fidum*, protarsomere 5 with the basal region about as long as the distal region (figure 26D in Xu et al. [2]). Following the description of *A. parafidum* sp. nov., the key to the females of the Oriental *Anteon* published by Xu et al. [2] should be modified by replacing couplet 68 as follows:

68. Protarsomere 5 with very large lamellae (Figure 9F) . . . 68′

- Protarsomere 5 with small lamellae (Plates 19F, 22A in Xu et al. [2]) . . . 69

68′. Protarsomere 5 with the basal region about as long as the distal region (Plate 26D in Xu et al. [2]) . . . *A. fidum* Olmi

- Protarsomere 5 with the basal part shorter than the distal part (Figure 9F) . . . *A. parafidum* sp. nov.

The male of *A. parafidum* sp. nov. is very similar to *A. fidum* Olmi, 1991, *A. priscum* Olmi, 1991, *A. shaanxianum* sp. nov. For the differences between *A. parafidum* and the other species, see updated keys for *A. shaanxianum* sp. nov.

#### 3.4.7. *Anteon priscum* Olmi (Figure 11)

*Anteon priscum* Olmi 1991: 164 [28]; Xu et al. 2013: 155 [2].

**Material examined**: 1 male, CHINA: Jiangsu, Nanjing, Xianlin, Mt. Duoshan, 32°6′51″ N 118°54′43″ E, 9–15.IV.2012, MT, Jie Zhao leg., SCAU 3011723 (SCBG); 1 female, Jiangsu, Nanjing, Xianlin, Mt. Duoshan, 32°6′51″ N 118°54′43″ E, 30.IV–7.V.2012, MT, Jie Zhao leg., SCAU 3011631 (SCBG).

**Distribution**. China (Gansu, Henan, Shaanxi, Ningxia, Jiangsu, Zhejiang, Fujian, Guangdong, Taiwan, Guizhou, Xizang, Yunnan), India, Indonesia [2].

**Figure 11:**
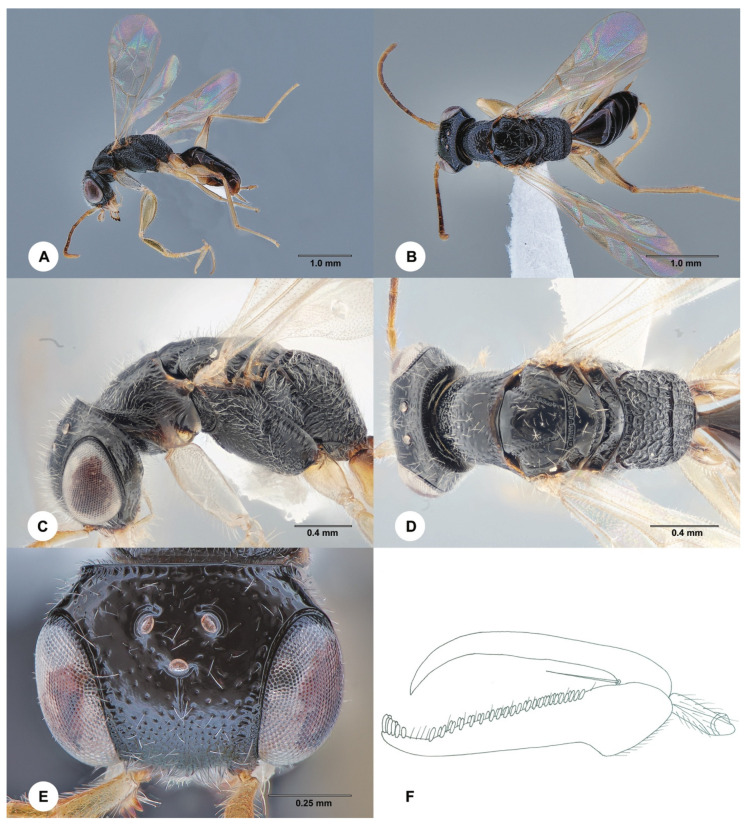
*Anteon priscum* Olmi, 1991, female (SCAU 3011631). (**A**) Habitus, lateral view. (**B**) Habitus, dorsal view. (**C**) Head and mesosoma, lateral view. (**D**) Head and mesosoma, dorsal view. (**E**) Head, dorsal view. (**F**) Chela.

**Remarks**. *A. priscum* was known only by males [2,28]. The female of this species is here confirmed by *COI* sequences. Therefore, we present the following diagnosis and description of the female:

**Description. Female.** Body length 4.1 mm; fully winged (Figure 11A,B). Head black with mandible and clypeus testaceous; antenna brown with scape and pedicel testaceous; mesosoma black; metasoma brown; legs yellow. Antenna clavate; antennomeres in the following proportions: 12:6:9:8:8:8:7:7:7:9. Head (Figure 11E) shiny, punctate, areas between punctures smooth; frontal line incomplete, shortly present in front of anterior ocellus; frons with two lateral longitudinal keels along orbits directed towards antennal toruli; OL = 4; OOL = 7; OPL = 6; POL = 5; TL = 6; greatest breadth of lateral ocellus shorter than OPL (3:6); occipital carina complete. Pronotum smooth, shiny, punctate, areas between punctures smooth; posterior surface of pronotum much longer than the anterior surface, shorter than mesoscutum (15:20); pronotal tubercle reaching tegula. Mesoscutum shiny, punctate, areas between punctures smooth. Notauli incomplete, present at the anterior 0.9 of the mesoscutum (Figure 11D). Mesoscutellum and metanotum shiny, unsculptured. Metapectal–propodeal complex with strong transverse keel between disc and propodeal declivity; disc reticulate rugose; propodeal declivity rugose, with two complete longitudinal keels. Forewing (Figure 11A) hyaline; distal part (Rs) of the stigmal vein (2r-rs&Rs) much shorter than the proximal (2r-rs) part (5:12). Protarsomeres in the following proportions: 8:3:4:14:25. Enlarged claw (Figure 11F) with proximal prominence bearing one long bristle. Protarsomere 5 (Figure 11F) with two rows of about 37 small lamellae and basal part much shorter than the distal part; distal apex with about 8 lamellae. Tibial spurs 1/1/2.

**Diagnosis of Female**: Head black, except mandible and clypeus testaceous; head punctate, areas between punctures smooth (Figure 11E); frontal line incomplete, shortly visible in front of anterior ocellus (Figure 11E); mesoscutum punctate, areas between punctures smooth (Figure 11D); notauli reaching approximately 0.9 × length of the mesoscutum (Figure 11D); propodeal declivity with two complete longitudinal keels and median area as rugose as lateral areas; distal part of the stigmal vein (2r-rs&Rs) less than 0.5 as long as the proximal part (Figure 11A); protarsomere 5 very slender (Figure 11F). Because of the above diagnosis, the female of *A. priscum* can be inserted in the key to the females of the Oriental *Anteon* published by Xu et al. [2] by modifying couplet 69 as follows:

69. Protarsomere 5 very broad (Plate 22A in Xu et al. 2013) . . . *A. caii* Xu, He & Olmi

- Protarsomere 5 very slender (figure 11F; Plate 19F in Xu et al. [2]) . . . 69′

69′. Clypeus black; frontal line complete; notauli reaching about 0.5 × length of the mesoscutum. . . *A. blanduscutum* Xu, He & Rui

- Clypeus testaceous; frontal line incomplete, shortly visible in front of anterior ocellus (Figure 11E); notauli reaching about 0.9 × length of the mesoscutum (Figure 11D) . . . *A. priscum* Olmi

#### 3.4.8. *Anteon shaanxianum* Chen, Olmi & Ødegaard, sp. nov. (Figure 12)

urn:lsid:zoobank.org:act:104C58C7-DA63-4DBA-BEE1-1F9C40F87488

**Material examined. Type**: male, holotype: CHINA: Shaanxi, Yang County, Huayang Town, 1154m, MT, 33.382156° N, 107.507905° E, 26.V–26.VI.2017, Haoyu Liu leg., SCAU 3040516 (SCBG).

**Distribution**. China (Shaanxi).

**Etymology**. The species is named after Shaanxi province, the locality of the holotype.

**Figure 12:**
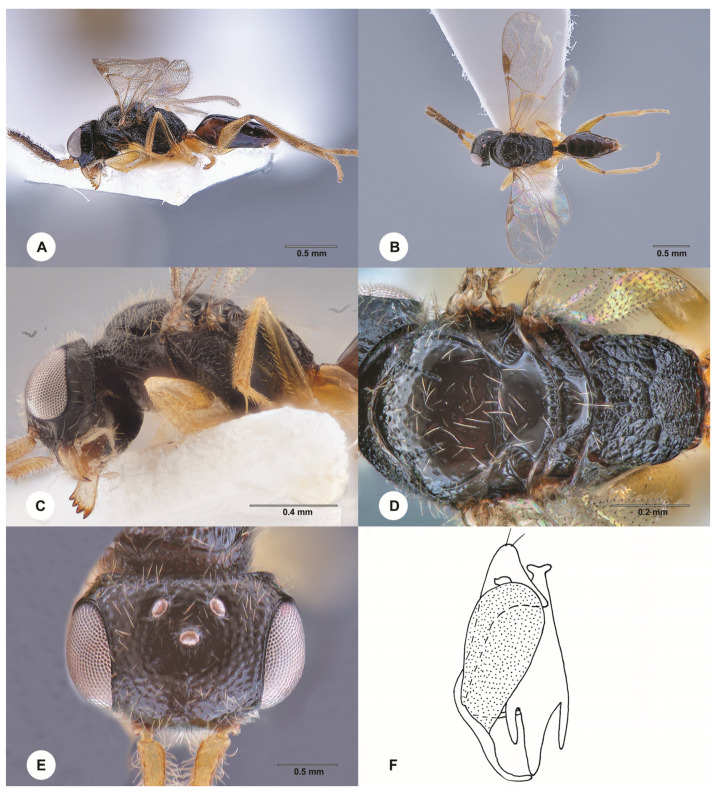
*Anteon shaanxianum* Chen, Olmi & Ødegaard, sp. nov., holotype, male (SCAU 3040516). (**A**) Habitus, lateral view. (**B**) Habitus, dorsal view. (**C**) Head and mesosoma, lateral view. (**D**) Mesosoma, dorsal view. (**E**) Head, dorsal view. (**F**) Genitalia (left half removed).

**Description. Male.** Body length 2.1 mm; fully winged (Figure 12A,B). Head black with mandible testaceous and clypeus ferruginous; antenna brown with scape yellow; mesosoma black; metasoma brown; legs yellow. Antenna filiform, with dense setae; antennomeres in the following proportions: 8:5:6:7:7:6:6:6:6:7. Head (Figure 12E) shiny, punctate, areas between punctures smooth; OL = 3; OOL = 5; OPL = 2; POL = 4; TL = 4; greatest breadth of the lateral ocellus as long as OPL; occipital carina complete; frontal line incomplete, present in front of the anterior ocellus and absent in the anterior half of frons; frons without lateral longitudinal keels along orbits directed towards antennal toruli. Mesoscutum punctate, areas between punctures smooth. Notauli incomplete, present at the anterior 0.7 of the scutum (Figure 12D). Mesoscutellum and metanotum shiny, unsculptured. Metapectal–propodeal complex with strong transverse keel between disc and propodeal declivity; disc reticulate rugose; propodeal declivity rugose, with two longitudinal keels. Forewing hyaline; distal part (Rs) of the stigmal vein (2r-rs&Rs) much shorter than the proximal (2r-rs) part (2.5:8). Paramere (Figure 12F) with distal inner pointed process; inner distal margin of pointed process slightly excavated. Dorsal process of paramere shorter than volsella (Figure 12F). Tibial spurs 1/1/2.

**Female**. Unknown.

**Diagnosis**. Male with head black and punctate, areas between punctures smooth; clypeus ferruginous; frontal line incomplete, present in front of the anterior ocellus and absent in the anterior half of frons; notauli present at the anterior 0.7 of mesoscutum; propodeal declivity with two complete longitudinal keels and median area as rugose as lateral areas; distal part of the stigmal vein (2r-rs&Rs) less than 0.5 as long as the proximal part; paramere with pointed distal inner process (Figure 12F), provided with inner distal margin slightly excavated; dorsal process of paramere shorter than volsella (Figure 12F); legs yellow.

*Anteon shaanxianum* sp. nov. is similar to *A. parafidum* sp. nov. and *A. fidum* Olmi, 1991, and the key to the males of the Oriental species of *Anteon* published by Xu et al. [2] should be modified by replacing couplet 59 as follows:

59. Paramere with distal inner rounded process (Plate 38I in Xu et al. [2]) . . . *A. parapriscum* Olmi

- Paramere with distal inner process more or less pointed (figures 10F and 12F; Plates 26C, 40B, C in Xu et al. [2]) . . . 60

60. Distal inner process of paramere with inner distal margin not excavated (Plate 40B, C in Xu et al. [2]) . . . *A. priscum* Olmi

- Distal inner process of paramere with inner distal margin slightly excavated (Figure 10F and Figure 12F; Plate 26C in Xu et al. [2]) . . . 60′

60′. Frontal line complete. . . *A. fidum* Olmi

- Frontal line absent or incomplete. . . 60″

60′’. Notauli present at about the anterior 0.5 of the mesoscutum (Figure 10D); frontal line absent; clypeus black. . . *A. parafidum* sp. nov.

- Notauli present at about the anterior 0.7 of the mesoscutum (Figure 12D); frontal line incomplete, present in front of the anterior ocellus and absent in the anterior half of frons; clypeus ferruginous. . . *A. shaanxianum* sp. nov.

#### 3.4.9. *Anteon shandonganum* Olmi, Chen & Liu, sp. nov. (Figure 13)

urn:lsid:zoobank.org:act:3BA2FB31-6A33-45F4-8BFE-DC90D3AF298A

**Material examined. Type**: holotype, female: CHINA: Shandong, Shanghe County, MT3, 37°16′4″ N 117°9′10″ E, 23–29.VI.2018, Jiahe Yan leg., SCAU 3011688 (SCBG).

**Distribution**. China (Shandong).

**Etymology**. The species is named after Shandong Province, the locality of the holotype.

**Figure 13:**
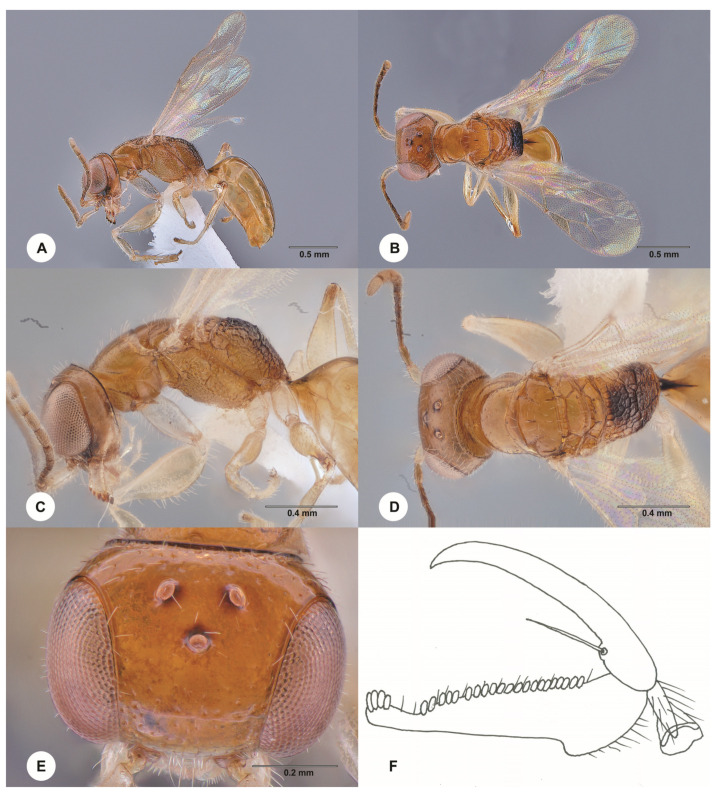
*Anteon shandonganum* Olmi, Chen & Liu, sp. nov., holotype, female (SCAU 3011688). (**A**) Habitus, lateral view. (**B**) Habitus, dorsal view. (**C**) Head and mesosoma, lateral view. (**D**) Head and mesosoma, dorsal view. (**E**) Head, dorsal view. (**F**) Chela.

**Description. Female, holotype.** Body length 2.2 mm; fully winged (Figure 13A,B). Body testaceous, except disc of metapectal–propodeal complex brown. Antenna clavate; antennomeres in the following proportions: 8:4:8:4:3:3:3:4:4:5. Head (Figure 13E) shiny, punctate, areas between punctures smooth; frontal line almost completely absent, only very shortly present in front of anterior ocellus; frons without lateral longitudinal keels along orbits directed towards antennal toruli; occipital carina complete; OL = 3; OOL = 4; OPL = 3; POL = 4; TL = 4; greatest breadth of the lateral ocellus shorter than OPL (2:3). Pronotum with the anterior surface much shorter than the posterior surface; posterior surface shiny, punctate, areas between punctures smooth, slightly shorter than mesoscutum (7:9); pronotal tubercle reaching tegula. Mesoscutum and mesoscutellum shiny, finely punctate, areas between punctures smooth. Notauli incomplete, present at the anterior 0.6 of the scutum (Figure 13D). Metapectal–propodeal complex with strong transverse keel between disc and propodeal declivity; disc reticulate rugose; propodeal declivity reticulate rugose, with two longitudinal keels. Forewing hyaline, without dark transverse bands; distal part (Rs) of the stigmal vein (2r-rs&Rs) much shorter than the proximal (2r-rs) part (3:8). Protarsomeres in the following proportions: 5:2:2:6:13. Enlarged claw (Figure 13F) with proximal prominence bearing one long bristle. Protarsomere 5 (Figure 13F) with the basal part much shorter than the distal part, with one row of about 21 lamellae; distal apex with four lamellae. Tibial spurs 1/1/2.

**Male**. Unknown.

**Diagnosis**. Female with head testaceous, punctate and areas between punctures smooth; frontal line almost completely absent, only very shortly present in front of anterior ocellus; ocellar triangle not delimited by keels; mesosoma mostly testaceous; posterior surface of the pronotum about as broad as long; mesoscutum slightly longer than the posterior region of pronotum; notauli present at about the anterior 0.6 of the mesoscutum; propodeal declivity reticulate rugose, with two longitudinal keels; forewing hyaline; protarsomere 4 slightly longer than 1; protarsomere 5 with the basal part much shorter than the distal part (Figure 13F).

*Anteon shandonganum* sp. nov. is similar to *A. quatei* Olmi, 1991. However, in *A. shandonganum*, the frontal line is almost completely absent, the mesoscutum is slightly longer than the posterior region of the pronotum, the notauli (Figure 13D) reach about 0.6 × length of the mesoscutum (in *A. quatei*, the frontal line is complete; mesoscutum about twice as long as the posterior region of pronotum; notauli reaching about 0.9 × length of the mesoscutum). Following the description of *A. shandonganum*, the key to the females of the Oriental *Anteon* published by Xu et al. [2] should be modified by replacing couplet 63 as follows:

63. Head black with mandible, clypeus and anterior region of face between antennal toruli brown-testaceous; notauli reaching about 0.25 × length of mesoscutum. . . *A. zonarium* Xu, Olmi & He

- Head totally or almost totally testaceous, partly darkened; notauli reaching about 0.6–0.9 × length of the mesoscutum. . . 63′

63′. Frontal line complete; mesoscutum about twice as long as the posterior region of the pronotum; notauli reaching about 0.9 × length of the mesoscutum. . . *A. quatei* Olmi

- Frontal line almost completely absent; mesoscutum slightly longer than the posterior region of pronotum (Figure 13D); notauli reaching about 0.6 × length of the mesoscutum (Figure 13D) . . . *A. shandonganum* sp. nov.

The key to the females of the Eastern Palaearctic *Anteon* published by Olmi & Xu [6] should be modified by replacing couplet 50 as follows:

50. Notauli reaching about 0.65–0.80 × length of the mesoscutum. . . *A. munitum* Olmi

- Notauli reaching 0.3–0.6 × length of the mesoscutum. . . 50′

50′. Notauli reaching about 0.3–0.5 × length of the mesoscutum. . . *A. ephippiger* (Dalman)

- Notauli reaching about 0.6 × length of the mesoscutum (Figure 13D) . . . *A. shandonganum* sp. nov.

## 4. Discussion

The pronounced sexual dimorphism in Dryinidae makes it difficult to associate the opposite sexes of the same species. In the past taxonomic practice of the sexually dimorphic genus *Anteon*, many species were described based on a single sex. Such a practice might result in that one species would have been described twice, leading to the exaggeration of a higher diversity. On the other hand, while some species were described by both sexes, the female and male were often associated tentatively, i.e., by the comparison of some morphological characters, or because they were collected in the same locality. However, such tentative associations might result in two species that have been lumped into one species, underestimating the real diversity of these parasitoids. DNA barcode-based methods have become powerful in delimitating species boundaries and the confirmation of female–male associations for parasitoid groups with extreme sexual dimorphism [10,30,31]. Our present study on *Anteon* further demonstrates that DNA barcoding is useful to enhance species delimitation and the sexual association of the same species in Dryinidae. The interspecific distances are larger than intraspecific distance for the COI sequences, with the intraspecific distances generally lower than 3%, while the interspecific distances are higher than 3.1% (Tables S2 and S3). The results produced by the two molecular species delimitation methods, i.e., ABGD and bPTP, are mostly congruent with the morphological identification. In addition, the sexual association of six species was confirmed by DNA sequences and lead us to synonymize *A. liui* with *A. meifenganum*.

Given the great potential of *Anteon* in the biological control of leafhoppers, the confirmation of the host association is also important. As indicated by a few researchers, DNA barcoding can be useful for clarifying host–parasitoid relationships in Dryinidae [9,13], which requires a more complete DNA barcode database as a reference. This study represents another effort towards the achievement of this goal.

## Figures and Tables

**Table 1:** List of sequenced species and accession numbers.

Code	Species	Sex	GenBank Accession No.
SCAU 3040430	*Anteon abdulnouri* Olmi, 1987	female	OR818495
SCAU 3011714	*Anteon achterbergi* Olmi, 1991	female	OR818496
SCAU 3011677	*Anteon blanduscutum* Xu, He & Rui, 1996	female	OR818497
SCAU 3011678	*Anteon blanduscutum* Xu, He & Rui, 1996	male	OR818498
SCAU 3011669	*Anteon borneanum* Olmi, 1984	female	OR818499
SCAU 3011671	*Anteon clariclypeum* sp. n.	female	OR818500
SCAU 3011712	*Anteon claricolle* Kieffer, 1906	male	OR818501
SCAU 3040561	*Anteon claricolle* Kieffer, 1906	female	OR818502
SCAU 3040517	*Anteon confusum* Olmi, 1991	female	OR818503
SCAU 3044058	*Anteon confusum* Olmi, 1991	male	OR818504
SCAU 3011621	*Anteon exiguum* (Haupt, 1941)	female	OR818505
SCAU 3040512	*Anteon fidum* Olmi, 1991	male	OR818506
SCAU 3040522	*Anteon funiuense* Xu, He & Olmi, 2001	female	OR818507
SCAU 3040511	*Anteon henanense* Xu, He & Olmi, 2001	male	OR818508
SCAU 3044072	*Anteon henanense* Xu, He & Olmi, 2001	female	OR818509
SCAU 3011713	*Anteon hilare* Olmi, 1984	female	OR818510
SCAU 3011676	*Anteon hirashimai* Olmi, 1993	female	OR818511
SCAU 3011738	*Anteon maguanense* sp. n.	female	OR818512
SCAU 3040514	*Anteon meifenganum* Olmi, 1991	female	OR818513
SCAU 3040520	*Anteon meifenganum* Olmi, 1991	male	OR818514
SCAU 3040515	*Anteon mite* Olmi, 1996	male	OR818515
SCAU 3011661	*Anteon multicolor* Xu, He & Olmi, 1998	female	OR818516
SCAU 3040571	*Anteon multicolor* Xu, He & Olmi, 1998	male	OR818517
SCAU 3040573	*Anteon multicolor* Xu, He & Olmi, 1998	male	OR818518
SCAU 3040574	*Anteon multicolor* Xu, He & Olmi, 1998	female	OR818519
SCAU 3044011	*Anteon multicolor* Xu, He & Olmi, 1998	male	OR818520
SCAU 3044014	*Anteon multicolor* Xu, He & Olmi, 1998	female	OR818521
SCAU 3044065	*Anteon multicolor* Xu, He & Olmi, 1998	male	OR818522
SCAU 3044074	*Anteon multicolor* Xu, He & Olmi, 1998	female	OR818523
SCAU 3044114	*Anteon multicolor* Xu, He & Olmi, 1998	male	OR818524
SCAU 3048997	*Anteon multicolor* Xu, He & Olmi, 1998	male	OR818525
SCAU 3040524	*Anteon munitum* Olmi, 1984	male	OR818526
SCAU 3040521	*Anteon naduense* Olmi 1987	female	OR818527
SCAU 3040433	*Anteon nanlingense* Xu, Olmi & He, 2011	female	OR818528
SCAU 3048821	*Anteon nanlingense* Xu, Olmi & He, 2011	male	OR818529
SCAU 3011658	*Anteon parafidum* sp. n.	female	OR818530
SCAU 3011662	*Anteon parafidum* sp. n.	male	OR818531
SCAU 3040566	*Anteon parafidum* sp. n.	female	OR818532
SCAU 3040567	*Anteon parafidum* sp. n.	male	OR818533
SCAU 3044018	*Anteon parafidum* sp. n.	male	OR818534
SCAU 3044026	*Anteon parafidum* sp. n.	female	OR818535
SCAU 3044033	*Anteon parafidum* sp. n.	female	OR818536
SCAU 3044066	*Anteon parafidum* sp. n.	male	OR818537
SCAU 3044088	*Anteon parafidum* sp. n.	male	OR818538
SCAU 3044089	*Anteon parafidum* sp. n.	female	OR818539
SCAU 3040518	*Anteon pilosum* Xu, Olmi & He, 2010	male	OR818540
SCAU 3011631	*Anteon priscum* Olmi, 1991	female	OR818541
SCAU 3011723	*Anteon priscum* Olmi, 1991	male	OR818542
SCAU 3040519	*Anteon pteromaculatum* Xu, Olmi, Guglielmino & Chen, 2012	female	OR818543
SCAU 3040516	*Anteon shaanxianum* sp. n.	male	OR818544
SCAU 3011688	*Anteon shandonganum* sp. n.	female	OR818545
SCAU 3040523	*Anteon tengchongense* Xu, He & Olmi, 1998	female	OR818546
SCAU 3011682	*Anteon yuani* Xu, He & Olmi, 1998	male	OR818547

## Data Availability

All data are available in this paper.

## Supplementary Materials

The following supporting information can be downloaded at https://www.mdpi.com/article/10.3390/insects15010018/s1, Figure S1: *Anteon abdulnouri* Olmi, 1987, female (SCAU 3040430); Figure S2: *Anteon achterbergi* Olmi, 1991, female (SCAU 3011714); Figure S3: *Anteon blanduscutum* Xu, He & Rui, 1996, female (SCAU 3011677); Figure S4: *Anteon borneanum* Olmi, 1984, female (SCAU 3011669); Figure S5: *Anteon confusum* Olmi, 1991, female (SCAU 3040517); Figure S6: *Anteon confusum* Olmi, 1991, male (SCAU 3044058); Figure S7: *Anteon exiguum* (Haupt, 1941), female (SCAU 3011621); Figure S8: *Anteon fidum* Olmi, 1991, male (SCAU 3040512); Figure S9: *Anteon funiuense* Xu, He & Olmi, 2001, female (SCAU 3040522); Figure S10: *Anteon henanense* Xu, He & Olmi, 2001, female (SCAU 3044072); Figure S11: *Anteon henanense* Xu, He & Olmi, 2001, male (SCAU 3040511); Figure S12: *Anteon hilare* Olmi, 1984, female (SCAU 3011713); Figure S13: *Anteon hirashimai* Olmi, 1993, female (SCAU 3011676); Figure S14: *Anteon mite* Olmi, 1996, male (SCAU 3040515); Figure S15: *Anteon multicolor* Xu, He & Olmi, 1998, female (SCAU 3011661); Figure S16: *Anteon multicolor* Xu, He & Olmi, 1998, male (SCAU 3044011); Figure S17: *Anteon munitum* Olmi, 1984, male (SCAU 3040524); Figure S18: *Anteon naduense* Olmi 1987, female (SCAU 3040521); Figure S19: *Anteon nanlingense* Xu, Olmi & He, 2011, female (SCAU 3040433); Figure S20: *Anteon nanlingense* Xu, Olmi & He, 2011, male (SCAU 3048821); Figure S21: *Anteon pilosum* Xu, Olmi & He, 2010, male (SCAU 3040518); Figure S22: *Anteon priscum* Olmi, 1991, male (SCAU 3011723); Figure S23: *Anteon pteromaculatum* Xu, Olmi, Guglielmino & Chen, 2012, female (SCAU 3040519); Figure S24: *Anteon tengchongense* Xu, He & Olmi, 1998, female (SCAU 3040523); Figure S25: *Anteon yuani* Xu, He & Olmi 1998, male (SCAU 3011682); Table S1: Details of sequenced specimens; Table S2: Genetic distance of COI within Anteon species under K2P model; Table S3: Interspecific pairwise distance of Anteon based on COI sequences (%).
