# Supplementary material for: DNA Barcoding Unveils New Species of the Sexually Dimorphic Genus Anteon Jurine (Hymenoptera, Dryinidae) from China
Source: Insects. 2023 Dec 30;15(1):18. doi: 10.3390/insects15010018 (PMC10816535; doi:10.3390/insects15010018)
Supplement: Supplementary file 1 [file insects-15-00018-s001.zip › insects-2766672-supplementary.pdf]

DNA Barcoding Unveils New Species of the Sexually Dimorphic Genus *Anteon* Jurine (Hymenoptera, Dryinidae) from China

Huayan Chen [1,*], Massimo Olmi [2], Frode Ødegaard [3], Leonardo Capradossi [4] and Jingxian Liu [5]

[1] Key Laboratory of Plant Resources Conservation and Sustainable Utilization, South China Botanical Garden, Chinese Academy of Sciences, Guangzhou 510650, China
[2] Tropical Entomology Research Center, Via De Gasperi 10, 01100 Viterbo, Italy; olmi@unitus.it
[3] Department of Natural History, Norwegian University of Science and Technology (NTNU), NO-7491 Trondheim, Norway; frode.odegaard@ntnu.no
[4] Independent Researcher, 01017 Tuscania, Italy; leonardocapradossi.lc@gmail.com
[5] Department of Entomology, South China Agricultural University, Guangzhou 510642, China; liujingxian@scau.edu.cn
* Correspondence: huayanc@scbg.ac.cn

Supplement material

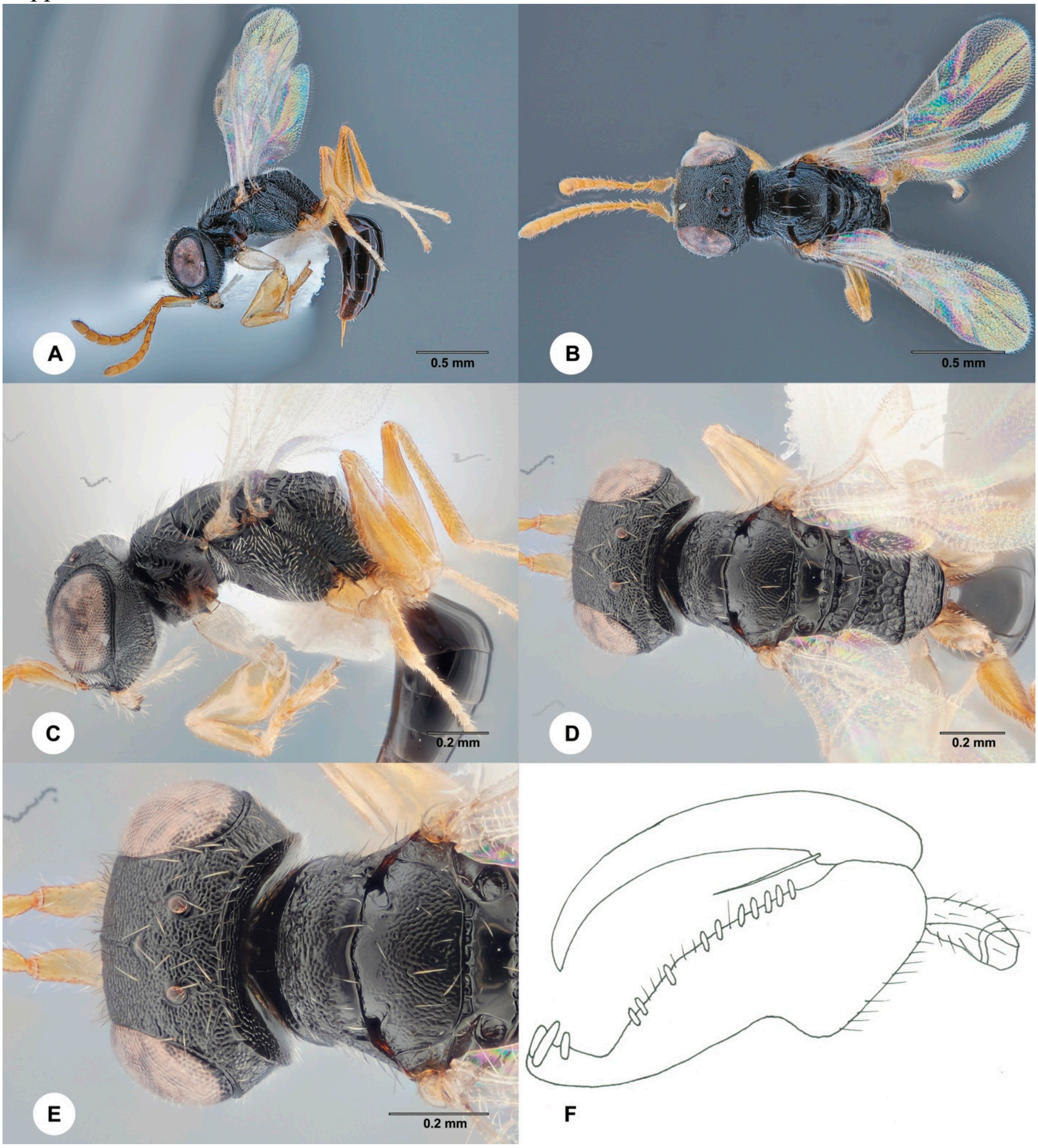

Figure S1. *Anteon abdulnouri* Olmi, 1987, female (SCAU 3040430) (**A**) Habitus, lateral view (**B**) Habitus, dorsal view (**C**) Head and mesosoma, lateral view (**D**) Head and mesosoma, dorsal view (**E**) Head and anterior mesosoma, dorsal view (**F**) Chela

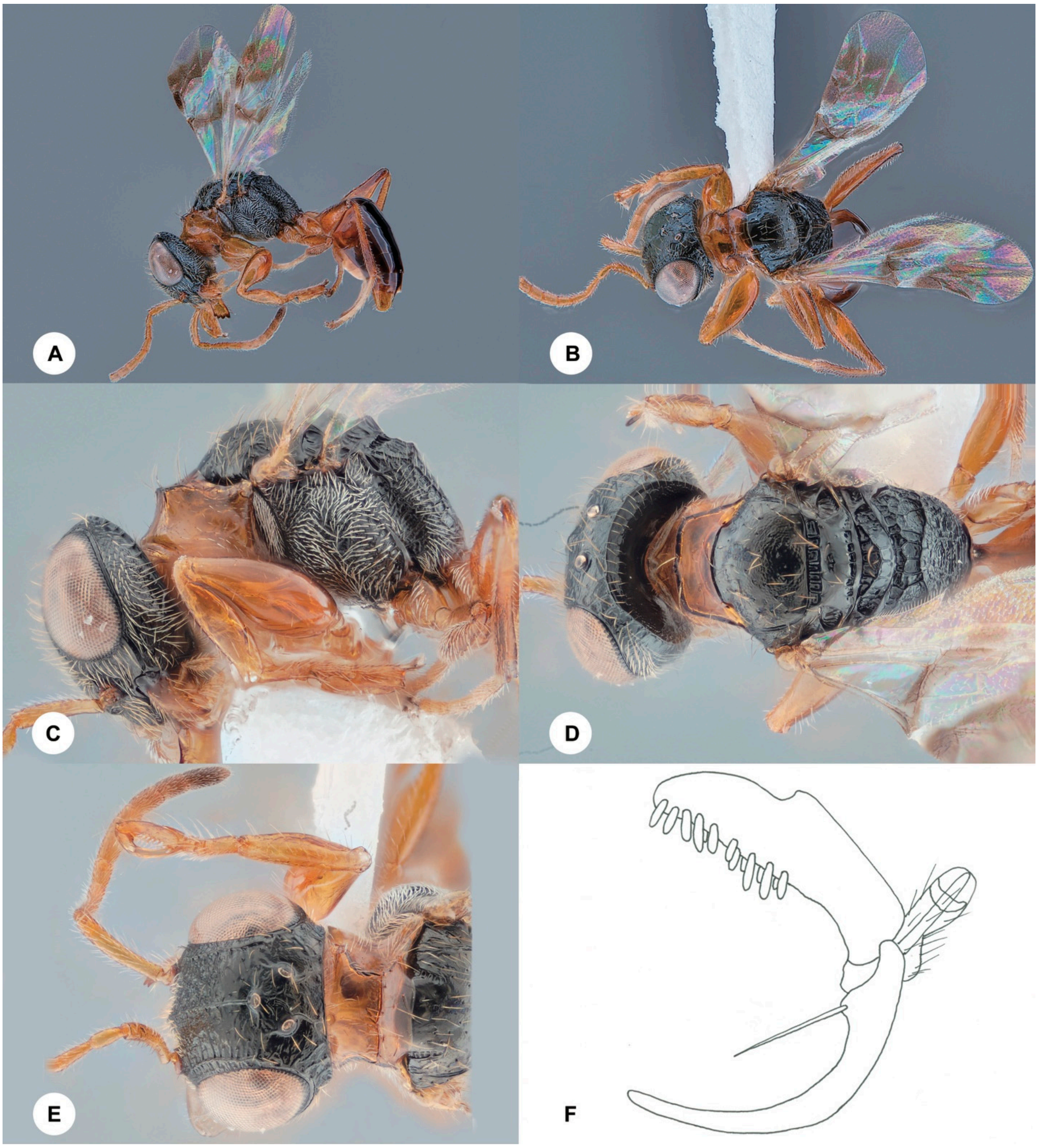

Figure S2. *Anteon achterbergi* Olmi, 1991, female (SCAU 3011714) (**A**) Habitus, lateral view (**B**) Habitus, dorsal view (**C**) Head and mesosoma, lateral view (**D**) Head and mesosoma, dorsal view (**E**) Head and anterior mesosoma, dorsal view (**F**) Chela

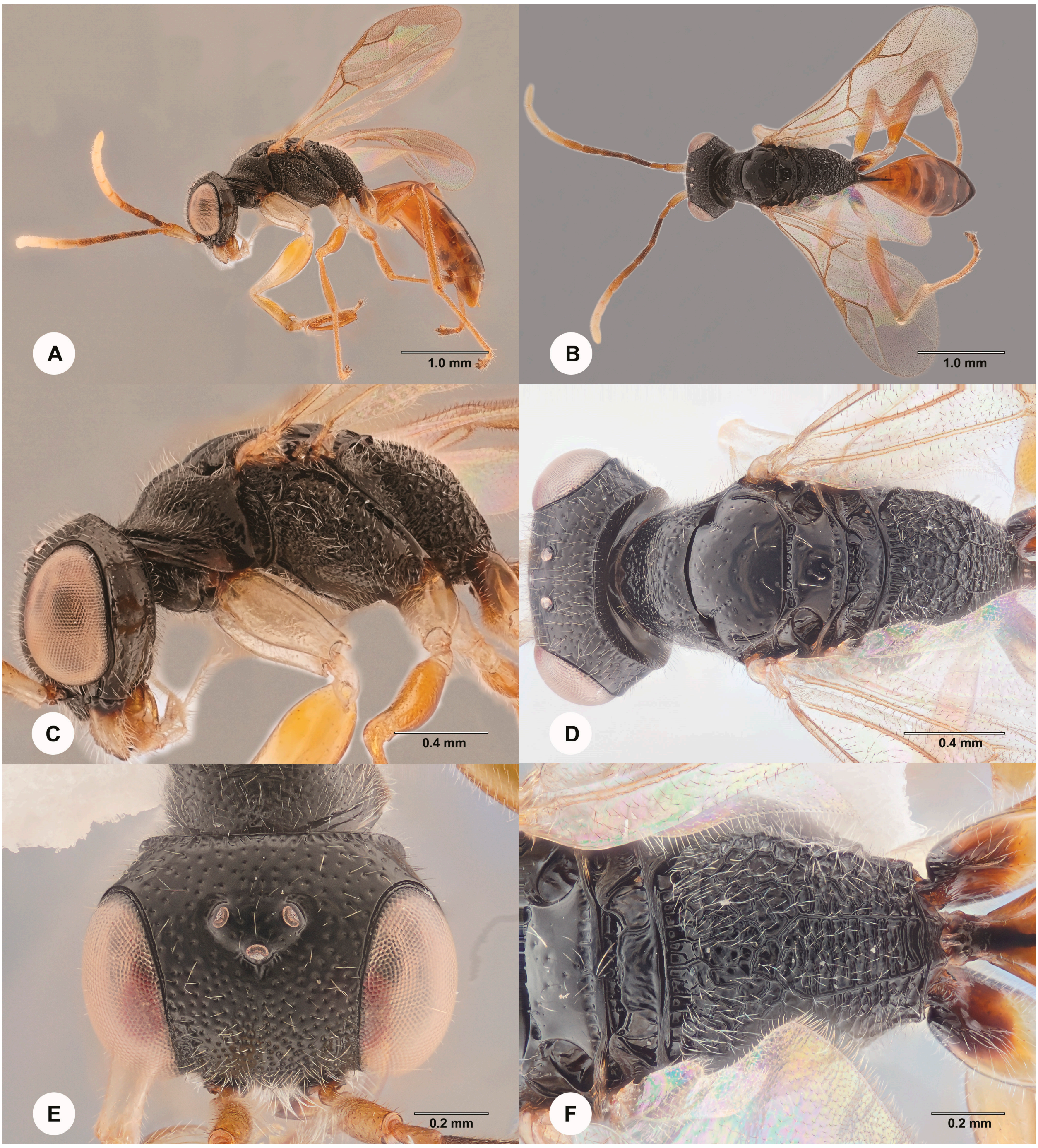

Figure S3. *Anteon blanduscutum* Xu, He & Rui, 1996, female (SCAU 3011677) (**A**) Habitus, lateral view (**B**) Habitus, dorsal view (**C**) Head and mesosoma, lateral view (**D**) Head and mesosoma, dorsal view (**E**) Head, dorsal view (**F**) Propodeum, dorsal view.

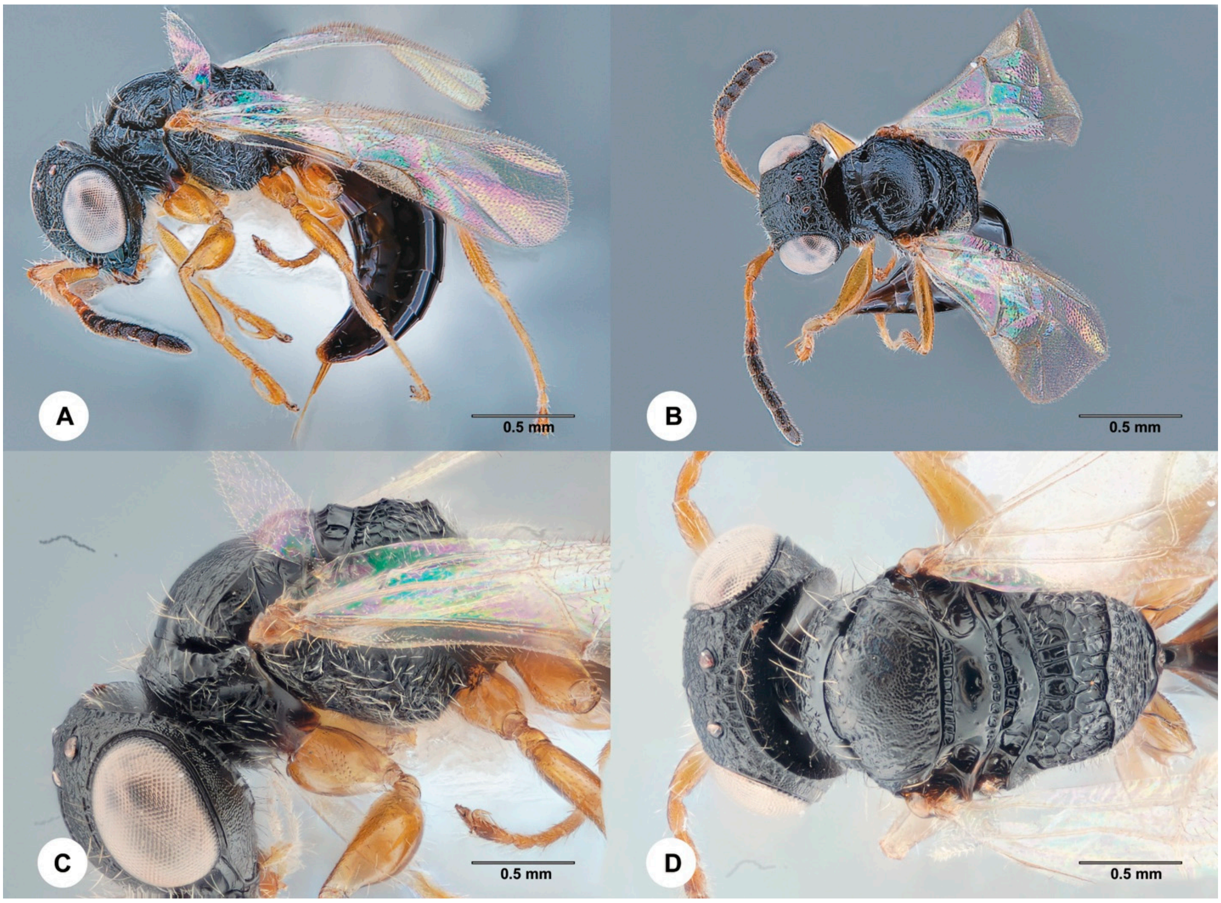

Figure S4. *Anteon borneanum* Olmi, 1984, female (SCAU 3011669) (**A**) Habitus, lateral view (**B**) Habitus, dorsal view (**C**) Head and mesosoma, lateral view (**D**) Head and mesosoma, dorsal view

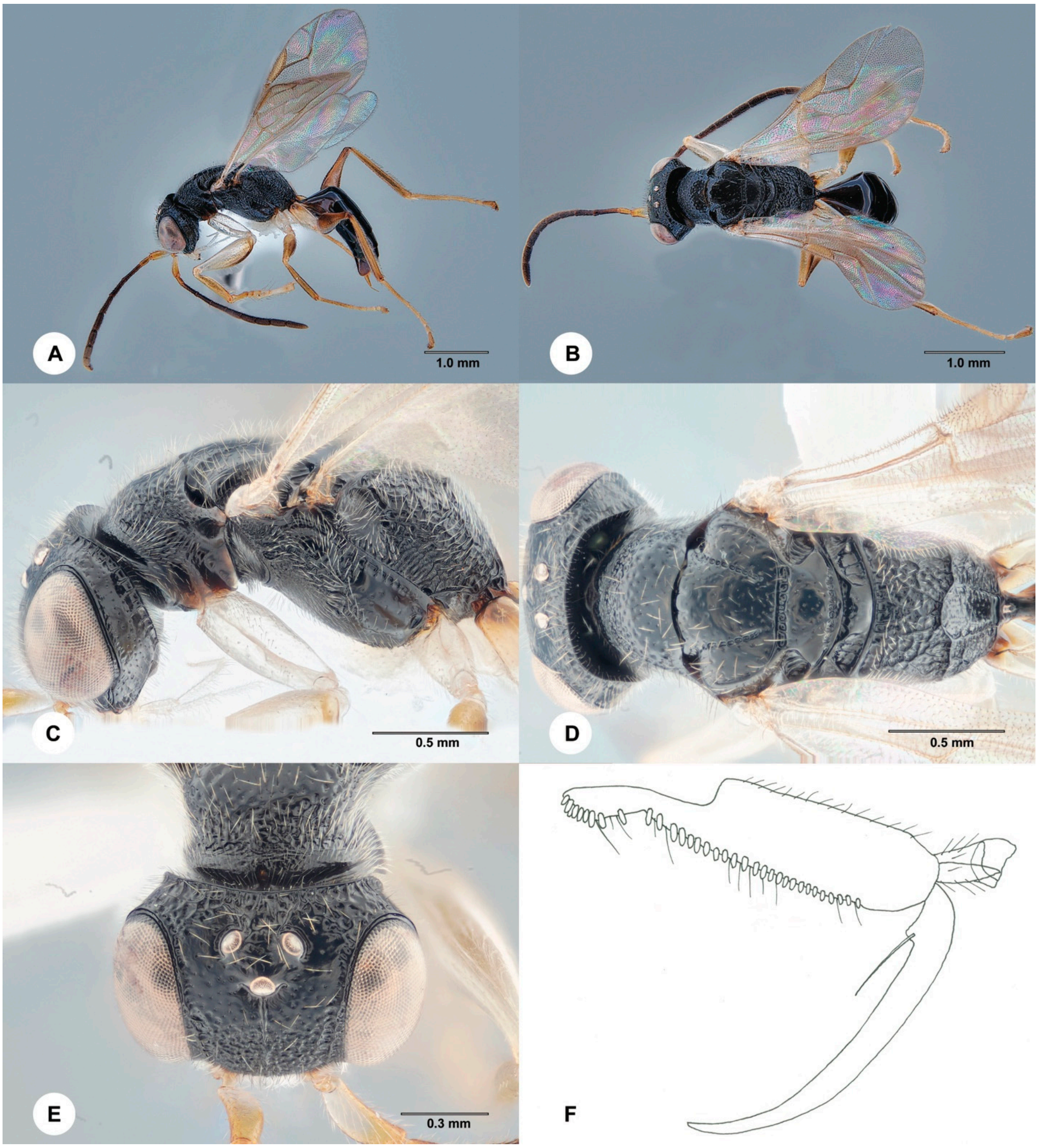

Figure S5. *Anteon confusum* Olmi, 1991, female (SCAU 3040517) (**A**) Habitus, lateral view (**B**) Habitus, dorsal view (**C**) Head and mesosoma, lateral view (**D**) Head and mesosoma, dorsal view (**E**) Head and anterior mesosoma, dorsal view (**F**) Chela

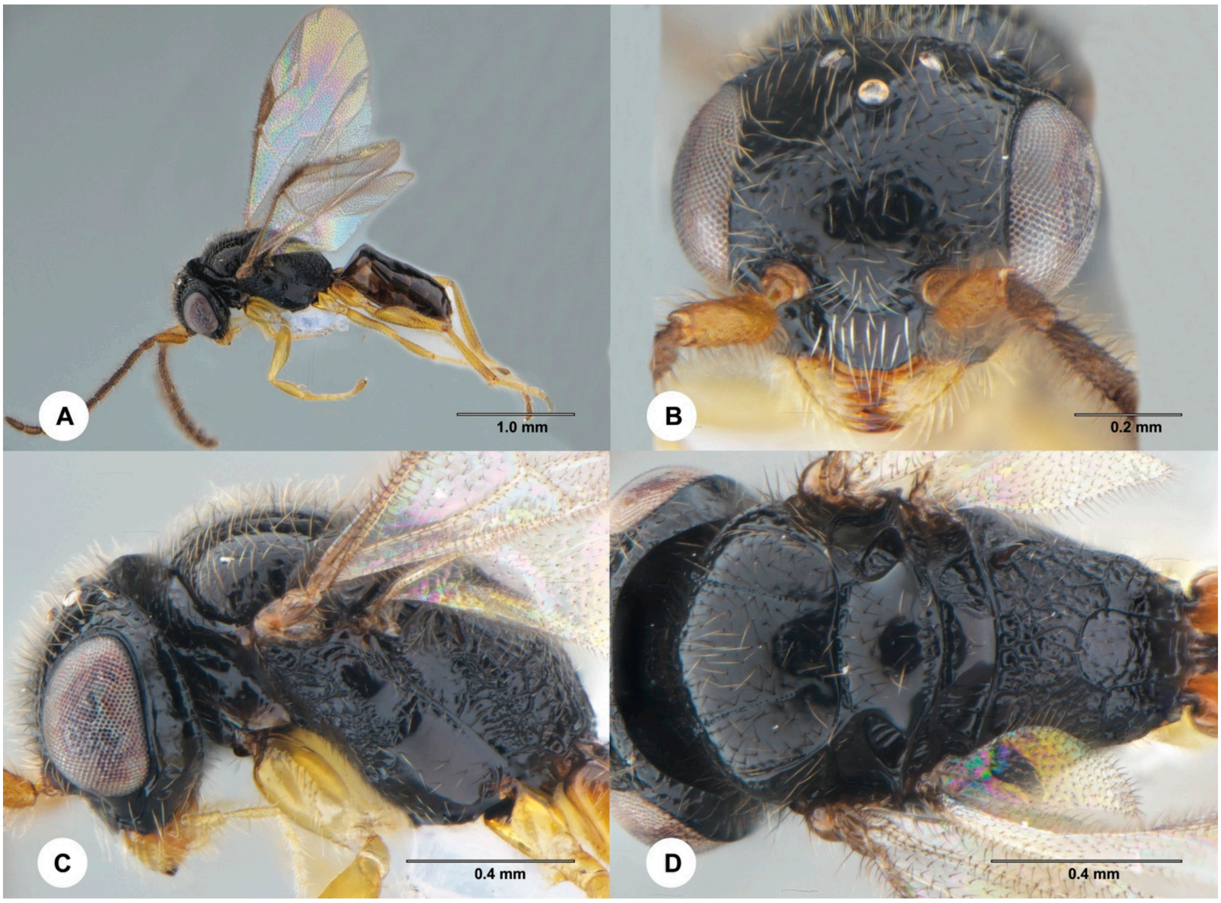

Figure S6. *Anteon confusum* Olmi, 1991, male (SCAU 3044058) (**A**) Habitus, lateral view (**B**) Habitus, dorsal view (**C**) Head and mesosoma, lateral view (**D**) Head and mesosoma, dorsal view

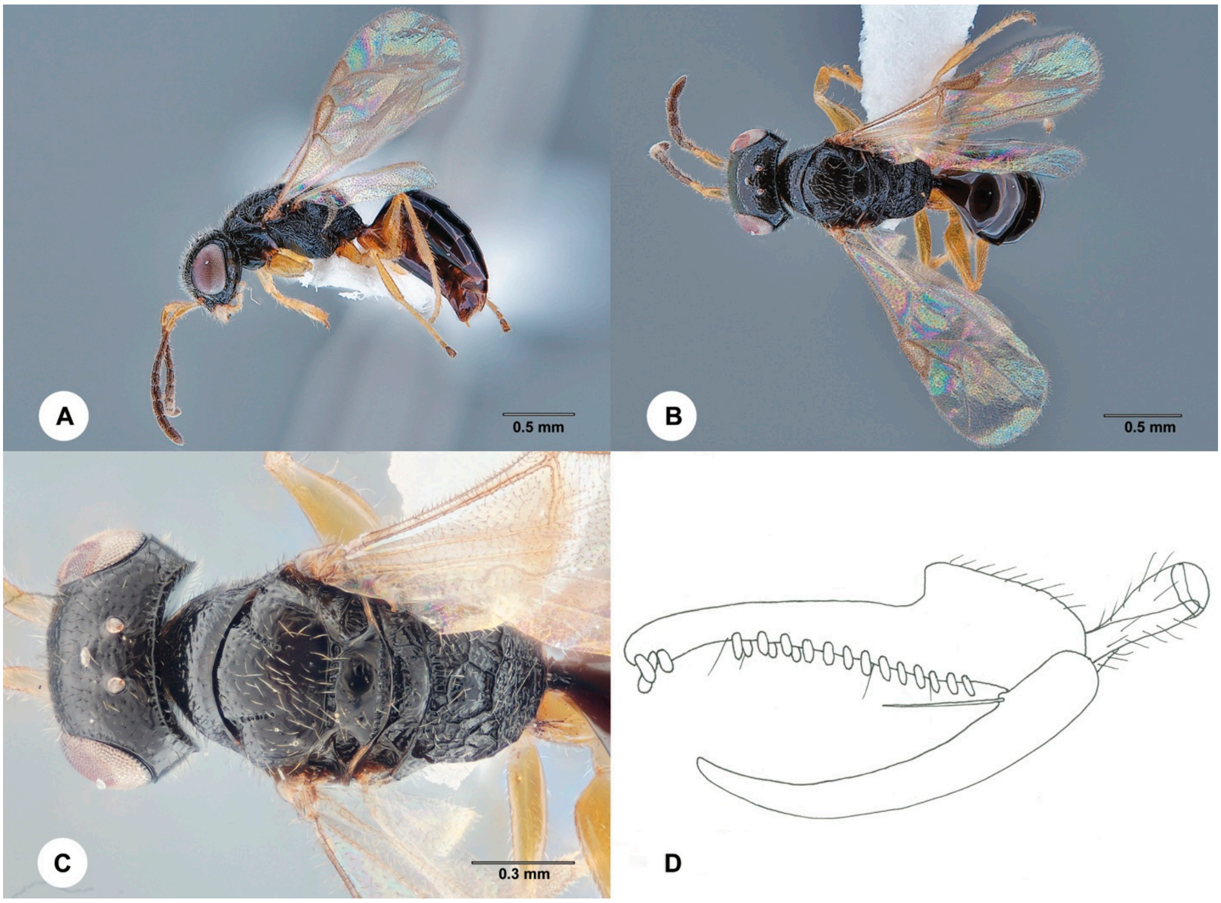

Figure S7. *Anteon exiguum* (Haupt, 1941), female (SCAU 3011621) (**A**) Habitus, lateral view (**B**) Habitus, dorsal view (**C**) Head and mesosoma, lateral view (**D**) Head and mesosoma, dorsal view (**E**) Head and anterior mesosoma, dorsal view (**F**) Chela

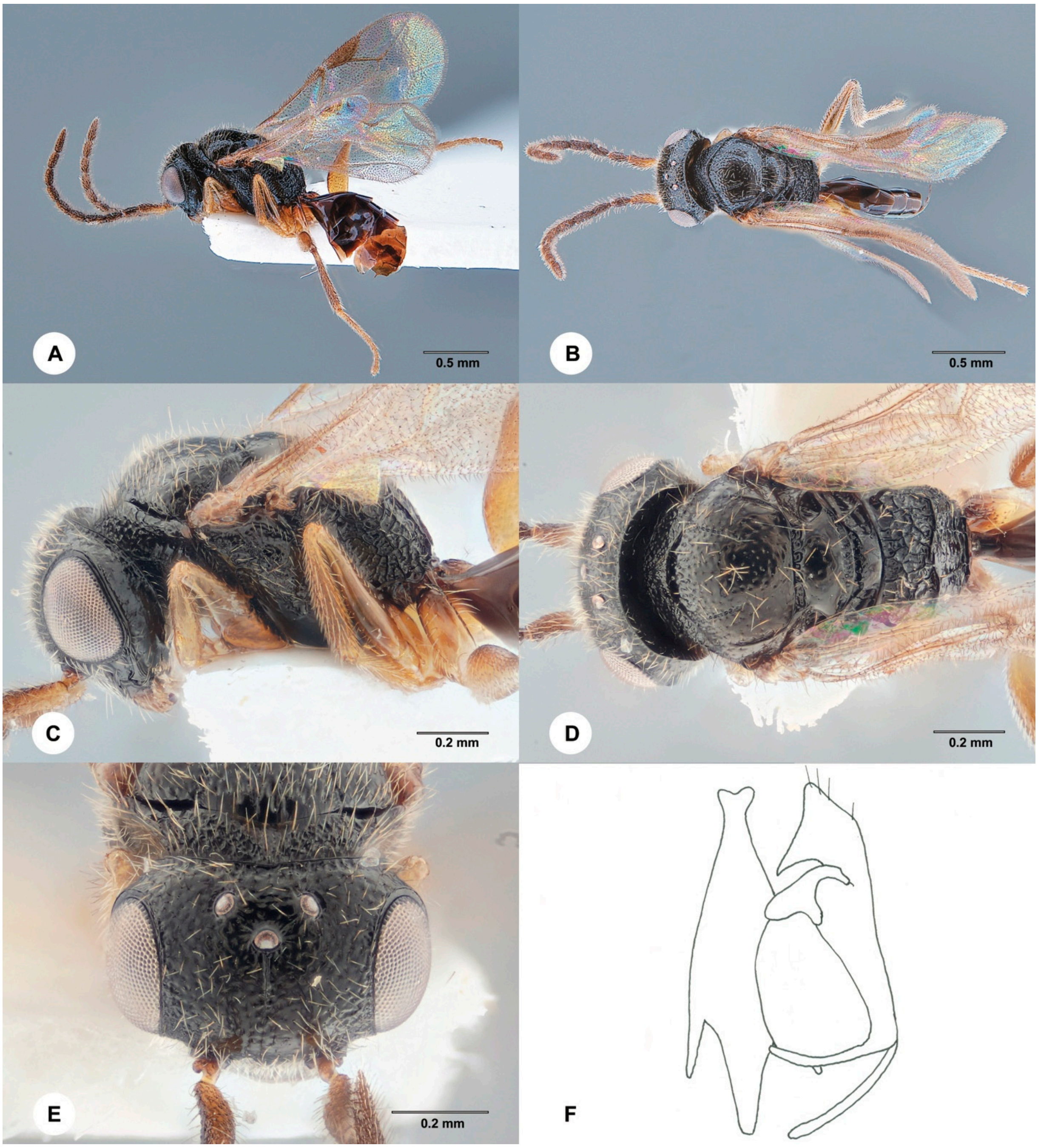

Figure S8. *Anteon fidum* Olmi, 1991, male (SCAU 3040512) (**A**) Habitus, lateral view (**B**) Habitus, dorsal view (**C**) Head and mesosoma, lateral view (**D**) Head and mesosoma, dorsal view (**E**) Head, dorsal view (**F**) Genitalia (left half removed)

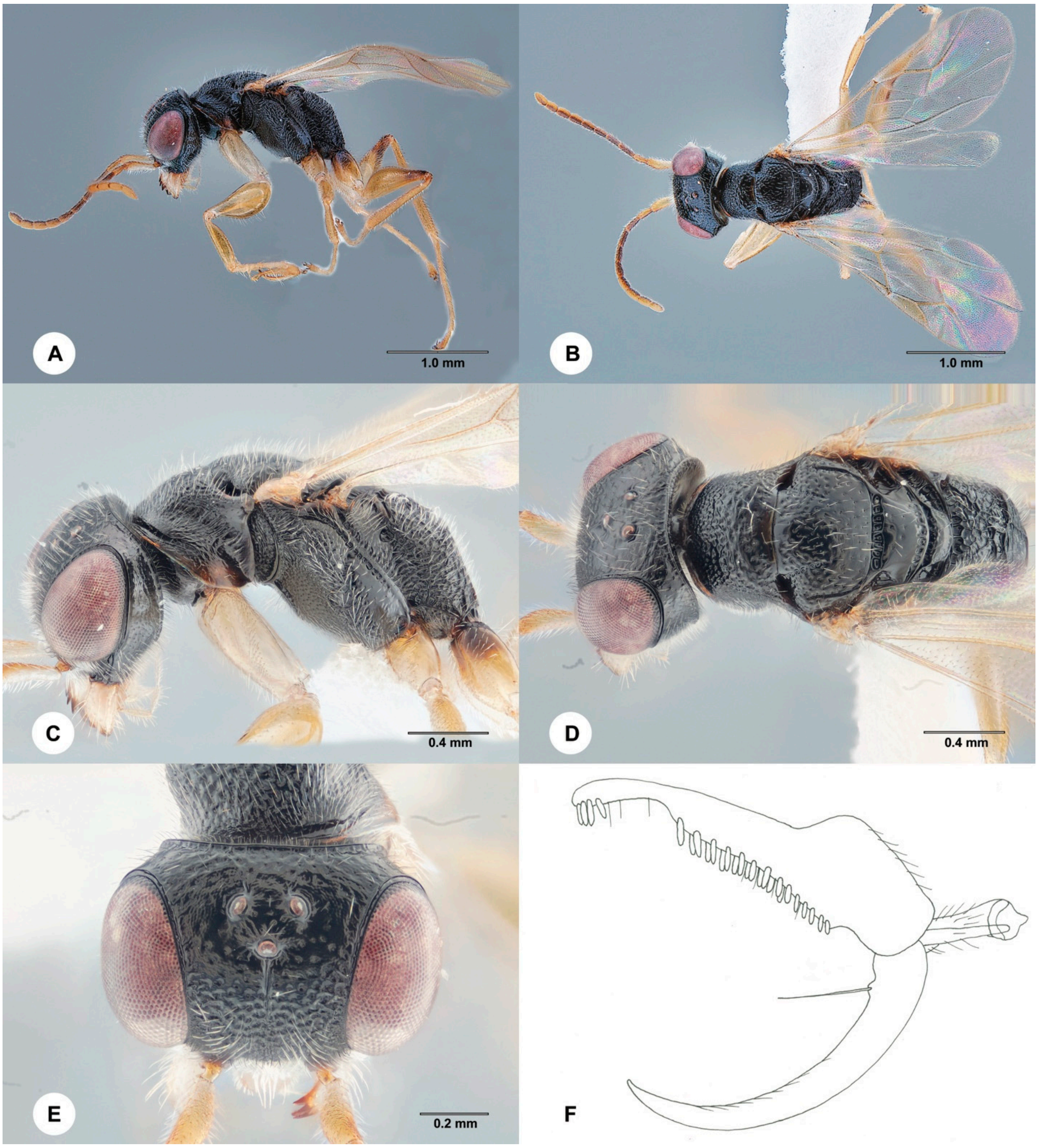

Figure S9. *Anteon funiuense* Xu, He & Olmi, 2001, female (SCAU 3040522) (**A**) Habitus, lateral view (**B**) Habitus, dorsal view (**C**) Head and mesosoma, lateral view (**D**) Head and mesosoma, dorsal view (**E**) Head, dorsal view (**F**) Chela.

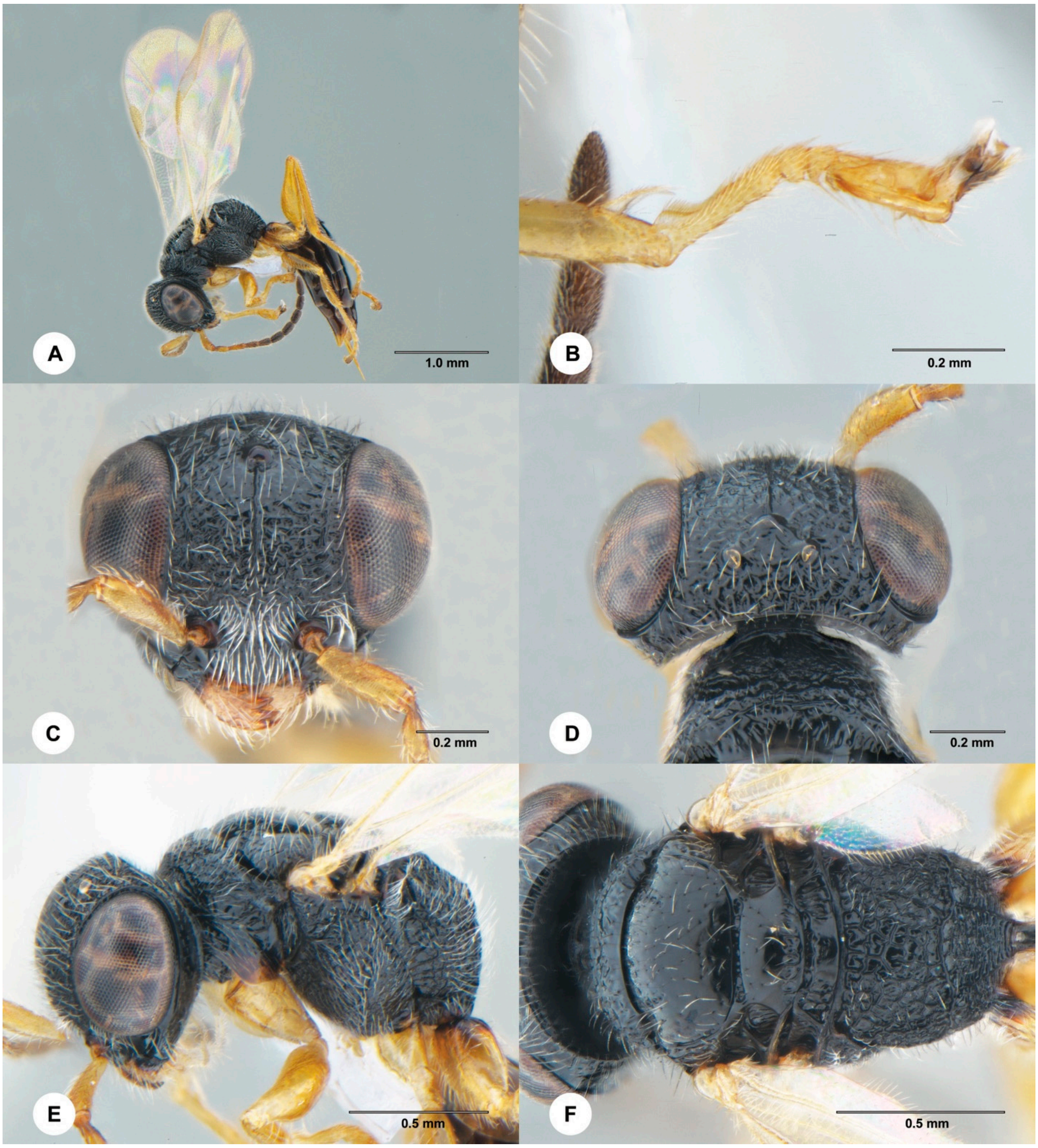

Figure S10. *Anteon henanense* Xu, He & Olmi, 2001, female (SCAU 3044072) (**A**) Habitus, lateral view (**B**) Chela (**C**) Head, anterior view (**D**) Head, dorsal view (**E**) Mesosoma, lateral view (**F**) Mesosoma, dorsal view.

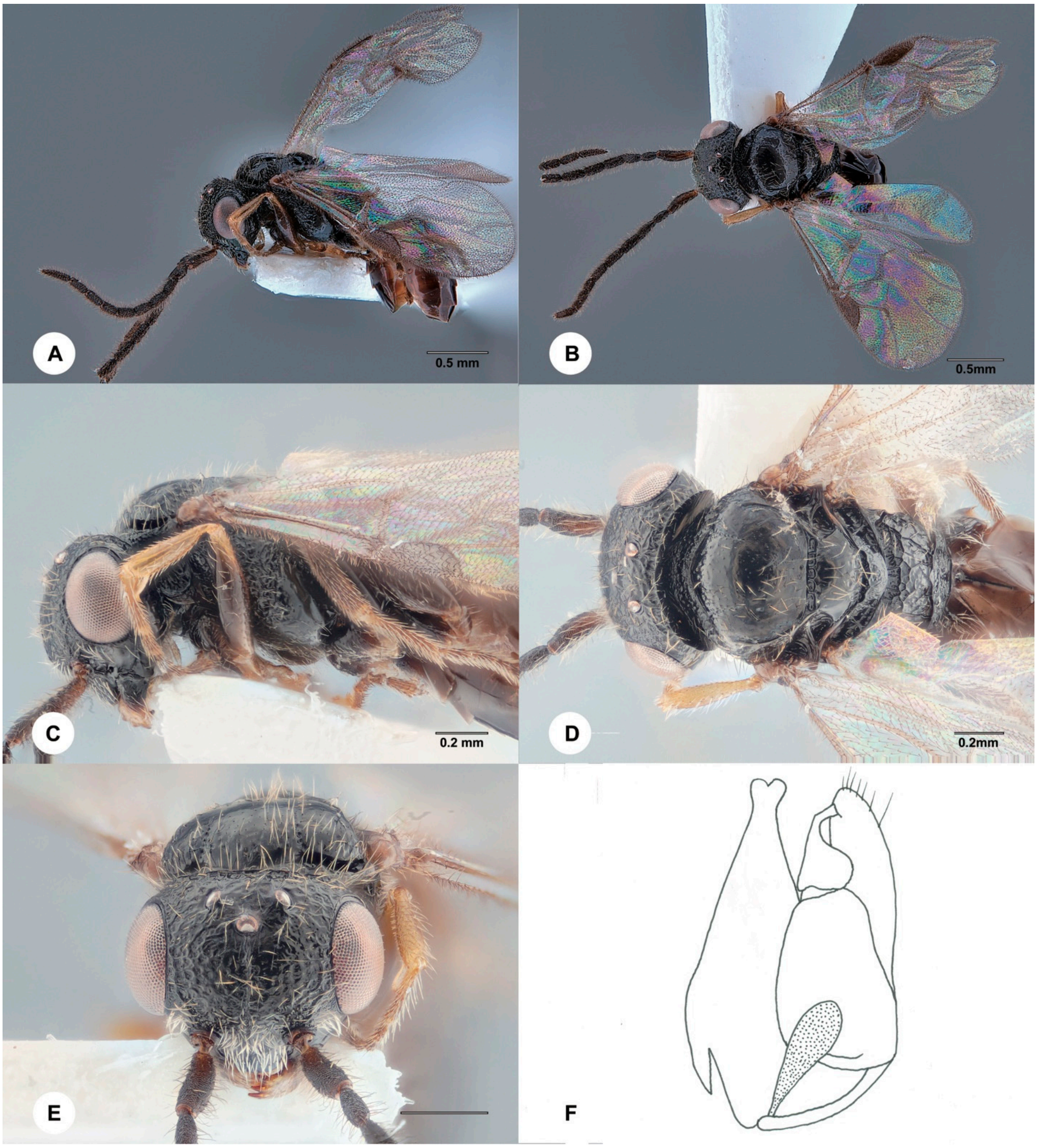

Figure S11. *Anteon henanense* Xu, He & Olmi, 2001, male (SCAU 3040511) (**A**) Habitus, lateral view (**B**) Habitus, dorsal view (**C**) Head and mesosoma, lateral view (**D**) Head and mesosoma, dorsal view (**E**) Head, anterior view (**F**) Genitalia (left half removed)

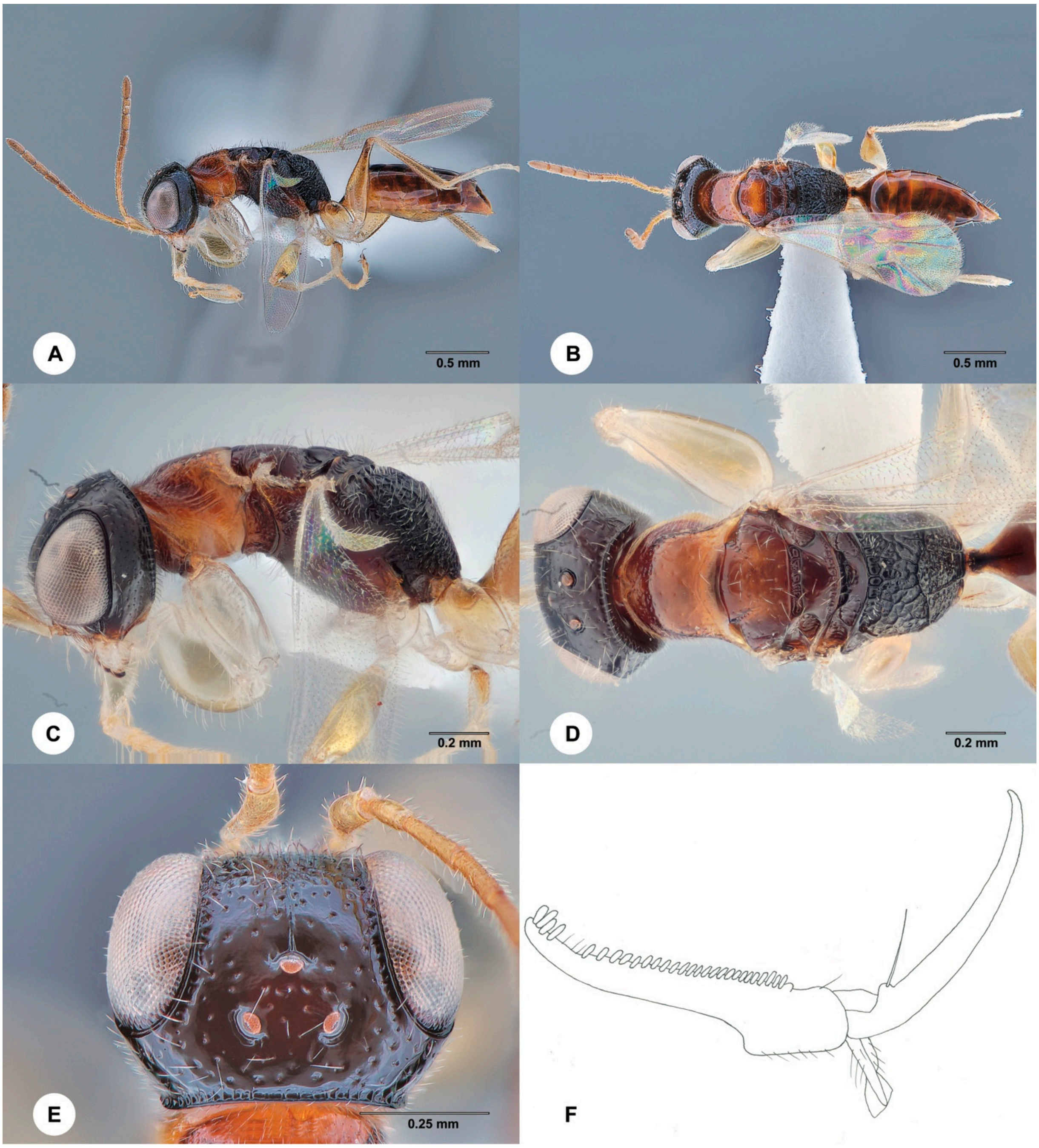

Figure S12. *Anteon hilare* Olmi, 1984, female (SCAU 3011713) (**A**) Habitus, lateral view (**B**) Habitus, dorsal view (**C**) Head and mesosoma, lateral view (**D**) Head and mesosoma, dorsal view (**E**) Head, dorsal view (**F**) Chela.

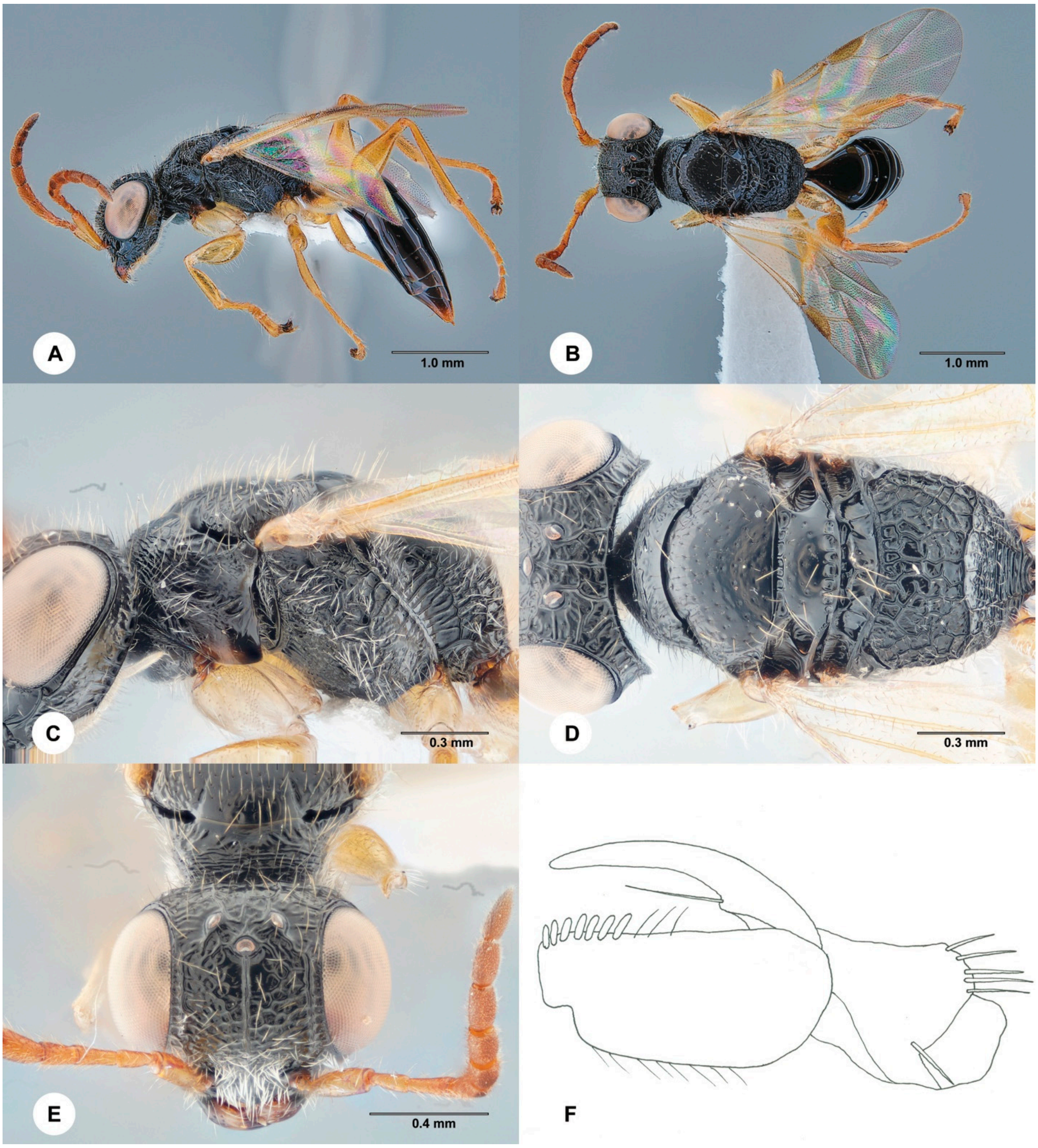

Figure S13. *Anteon hirashimai* Olmi, 1993, female (SCAU 3011676) (**A**) Habitus, lateral view (**B**) Habitus, dorsal view (**C**) Head and mesosoma, lateral view (**D**) Head and mesosoma, dorsal view (**E**) Head, dorsal view (**F**) Chela.

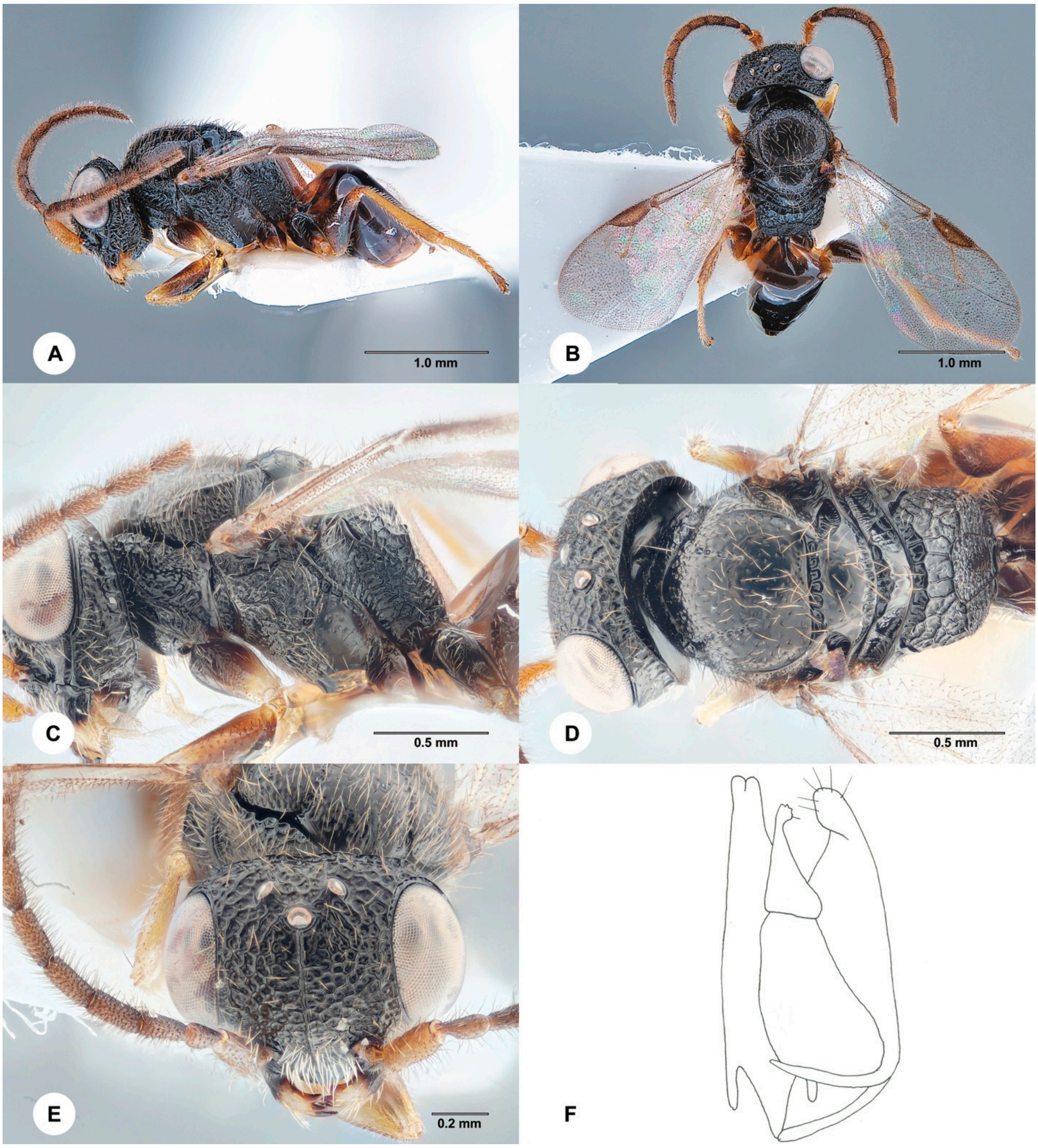

Figure S14. *Anteon mite* Olmi, 1996, male (SCAU 3040515) (**A**) Habitus, lateral view (**B**) Habitus, dorsal view (**C**) Head and mesosoma, lateral view (**D**) Head and mesosoma, dorsal view (**E**) Head, anterior view (**F**) Genitalia (left half removed).

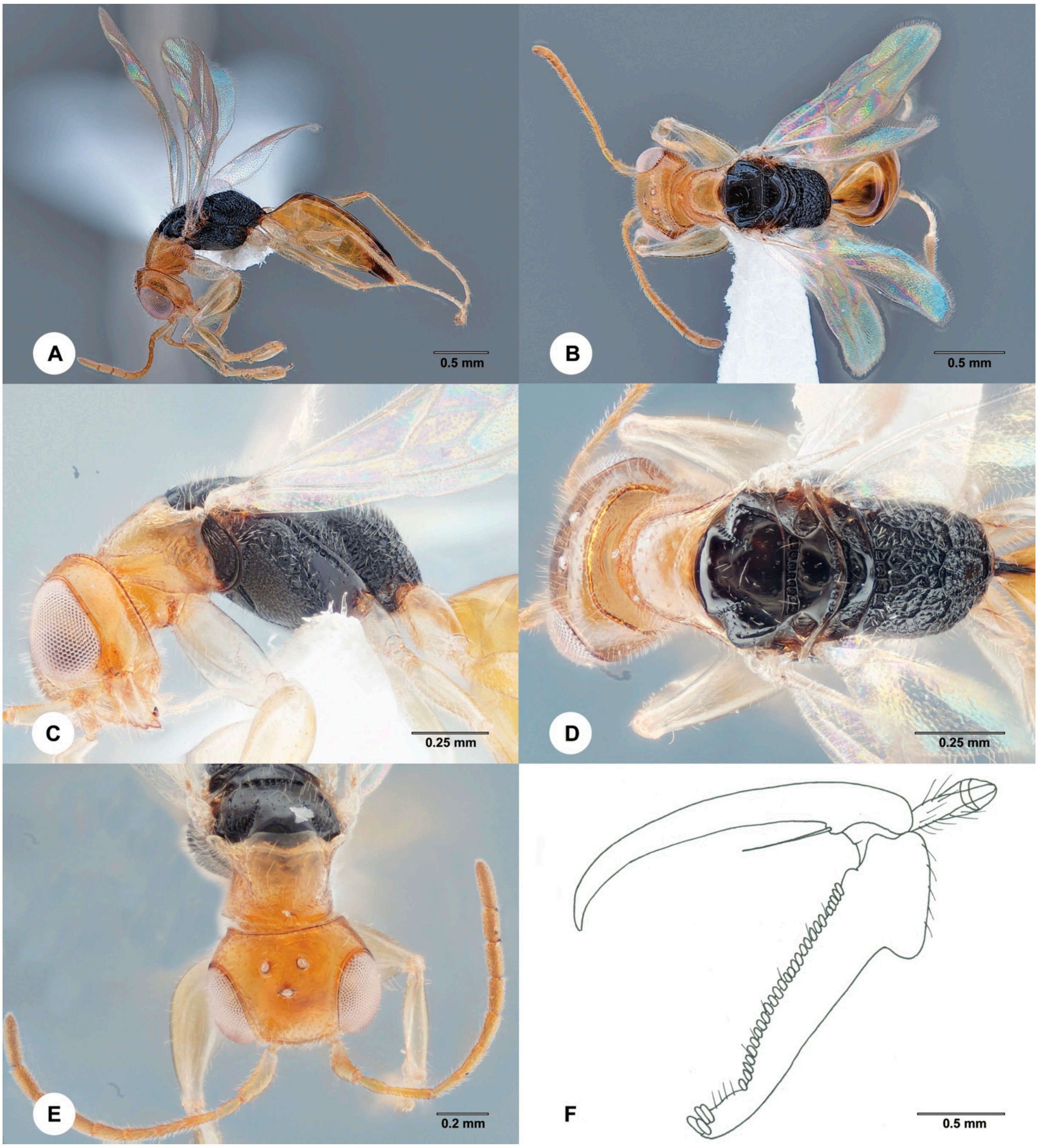

Figure S15. *Anteon multicolor* Xu, He & Olmi, 1998, female (SCAU 3011661) (**A**) Habitus, lateral view (**B**) Habitus, dorsal view (**C**) Head and mesosoma, lateral view (**D**) Head and mesosoma, dorsal view (**E**) Head, dorsal view (**F**) Chela.

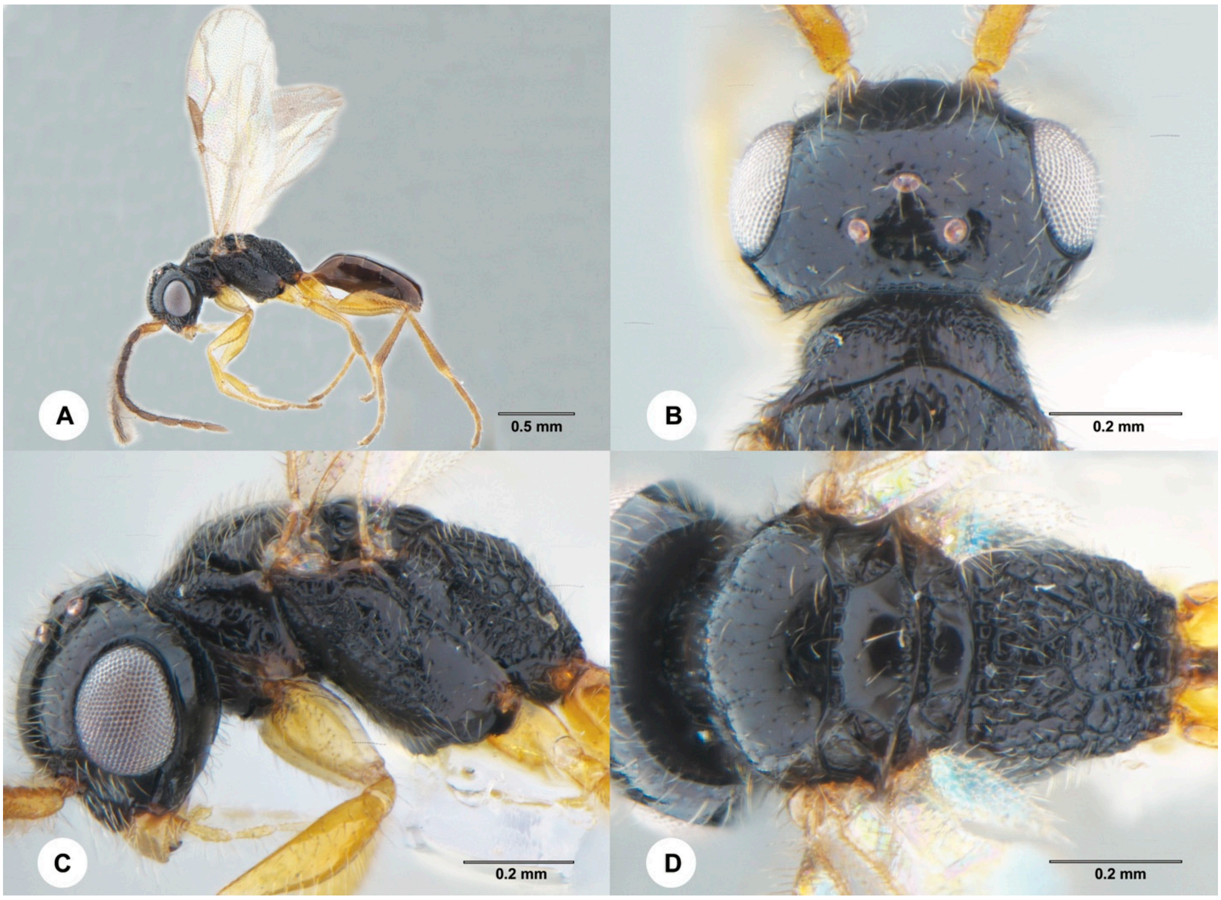

Figure S16. *Anteon multicolor* Xu, He & Olmi, 1998, male (SCAU 3044011) (**A**) Habitus, lateral view (**B**) Head, dorsal view (**C**) Head and mesosoma, lateral view (**D**) Head and mesosoma, dorsal view.

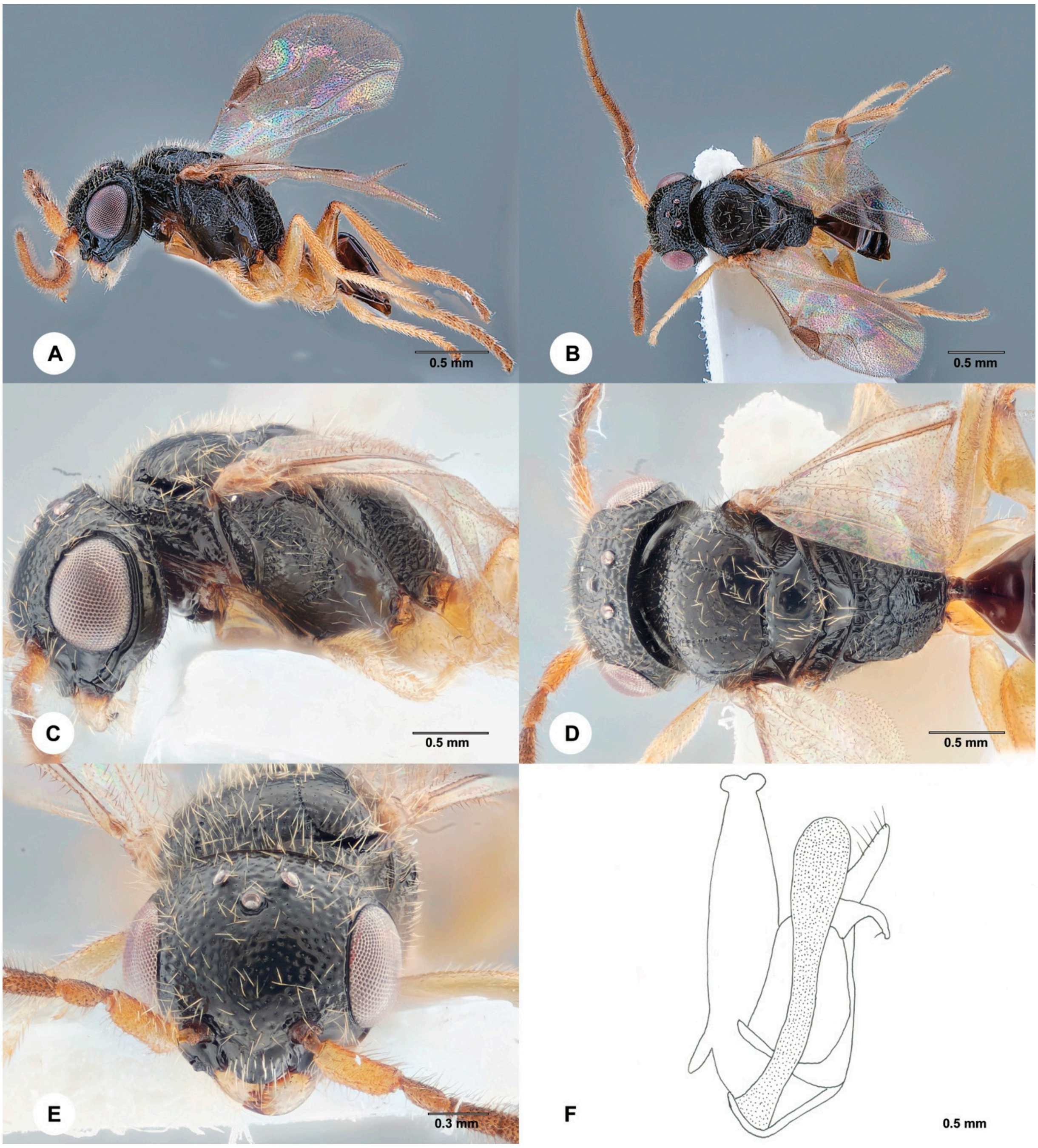

Figure S17. *Anteon munitum* Olmi, 1984, male (SCAU 3040524) (**A**) Habitus, lateral view (**B**) Habitus, dorsal view (**C**) Head and mesosoma, lateral view (**D**) Head and mesosoma, dorsal view (**E**) Head, anterior view (**F**) Genitalia (left half removed).

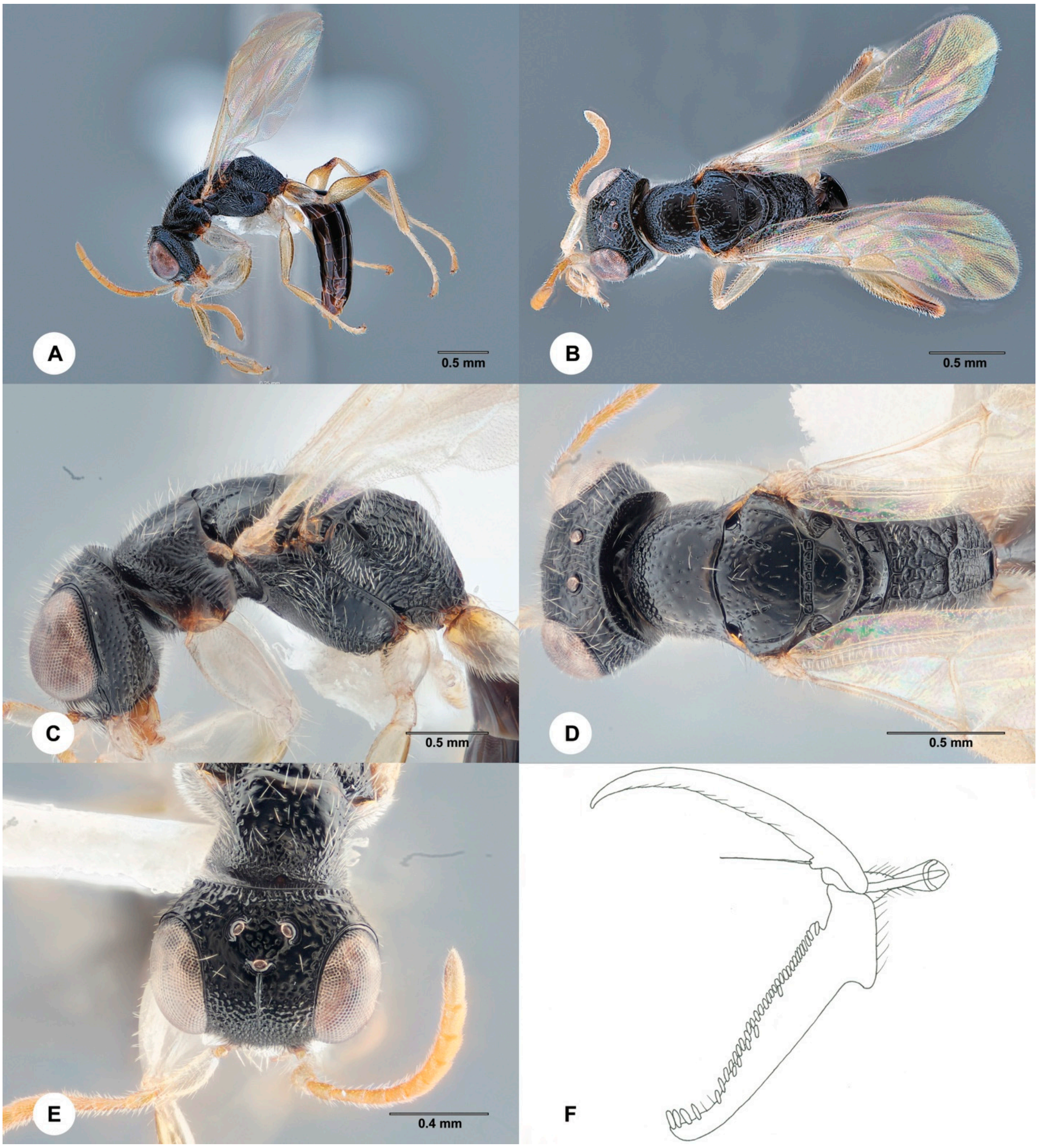

Figure S18. *Anteon naduense* Olmi 1987, female (SCAU 3040521) (**A**) Habitus, lateral view (**B**) Habitus, dorsal view (**C**) Head and mesosoma, lateral view (**D**) Head and mesosoma, dorsal view (**E**) Head, dorsal view (**F**) Chela.

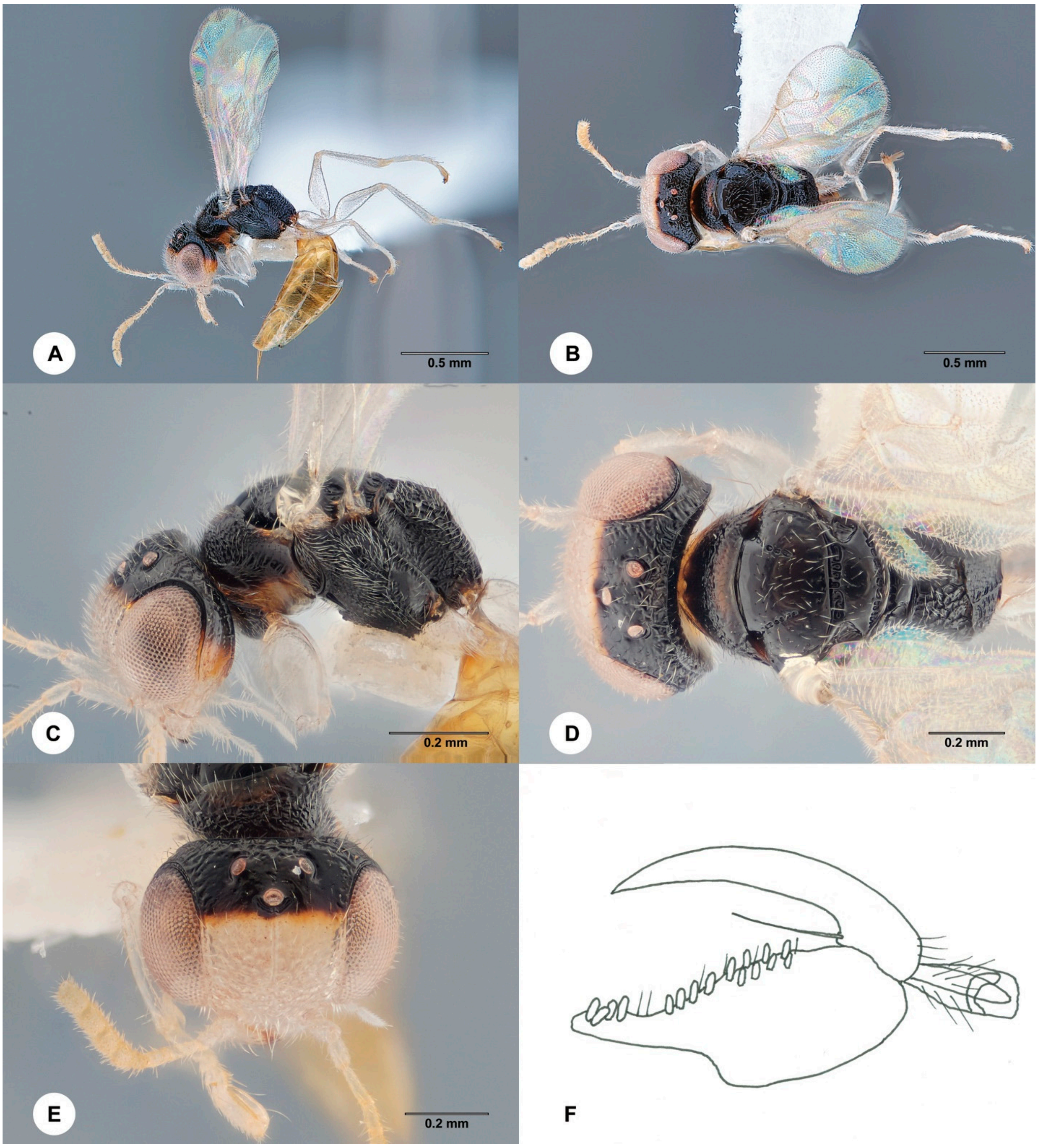

Figure S19. *Anteon nanlingense* Xu, Olmi & He, 2011, female (SCAU 3040433) (**A**) Habitus, lateral view (**B**) Habitus, dorsal view (**C**) Head and mesosoma, lateral view (**D**) Head and mesosoma, dorsal view (**E**) Head, anterior view (**F**) Chela.

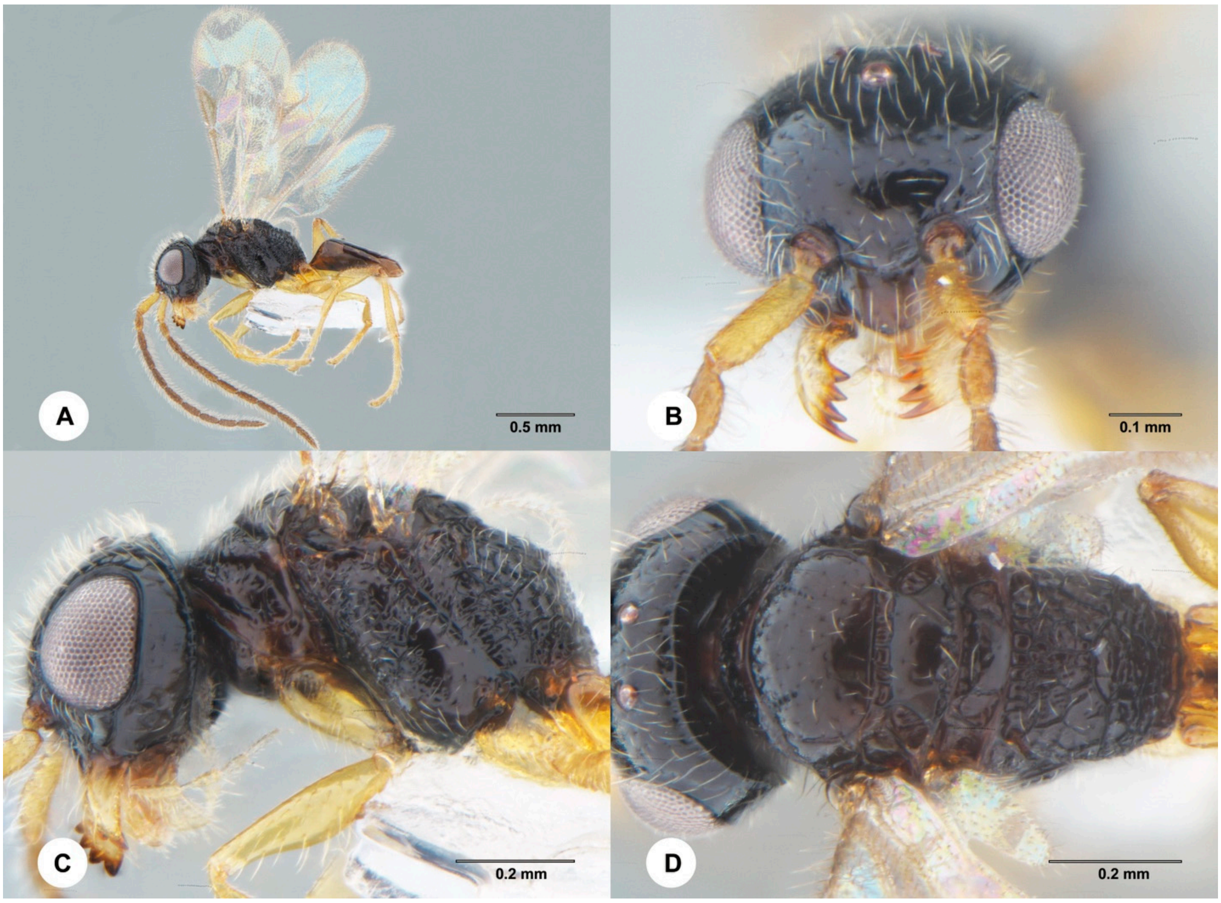

Figure S20. *Anteon nanlingense* Xu, Olmi & He, 2011, male (SCAU 3048821) (**A**) Habitus, lateral view (**B**) Head, dorsal view (**C**) Head and mesosoma, lateral view (**D**) Head and mesosoma, dorsal view.

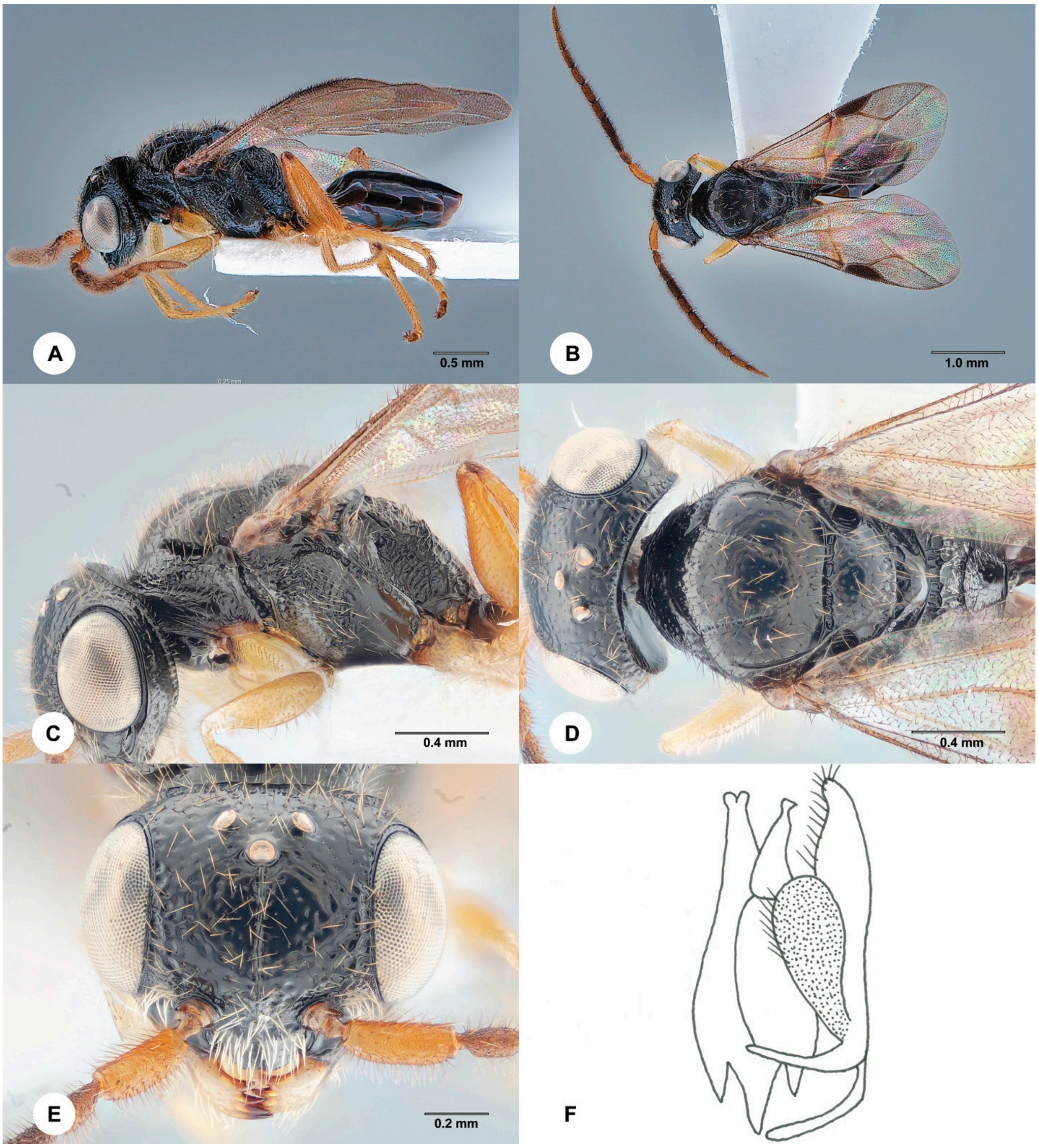

Figure S21. *Anteon pilosum* Xu, Olmi & He, 2010, male (SCAU 3040518) (**A**) Habitus, lateral view (**B**) Habitus, dorsal view (**C**) Head and mesosoma, lateral view (**D**) Head and mesosoma, dorsal view (**E**) Head, anterior view (**F**) Genitalia (left half removed).

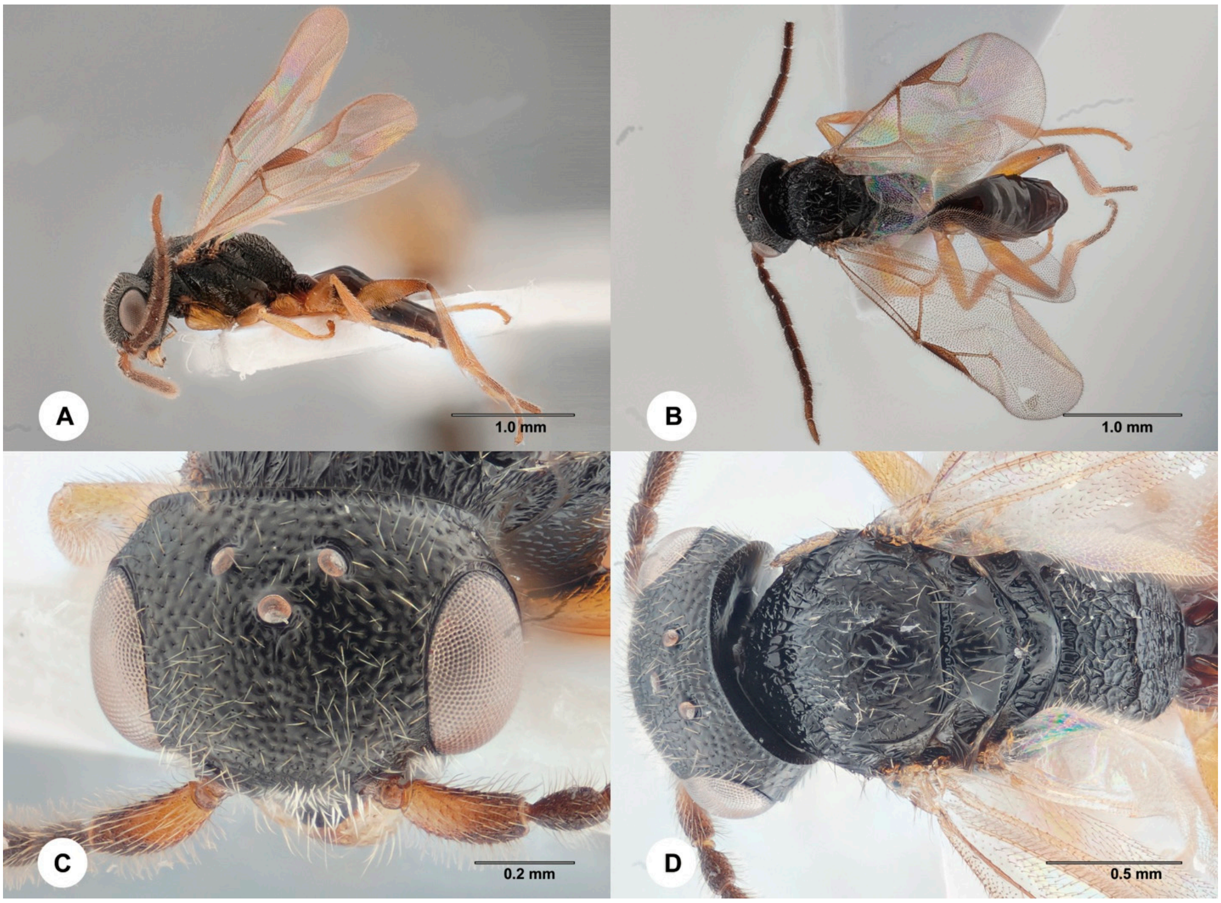

Figure S22. *Anteon priscum* Olmi, 1991, male (SCAU 3011723) (**A**) Habitus, lateral view (**B**) Habitus, dorsal view (**C**) Head, dorsal view (**D**) Head and mesosoma, dorsal view.

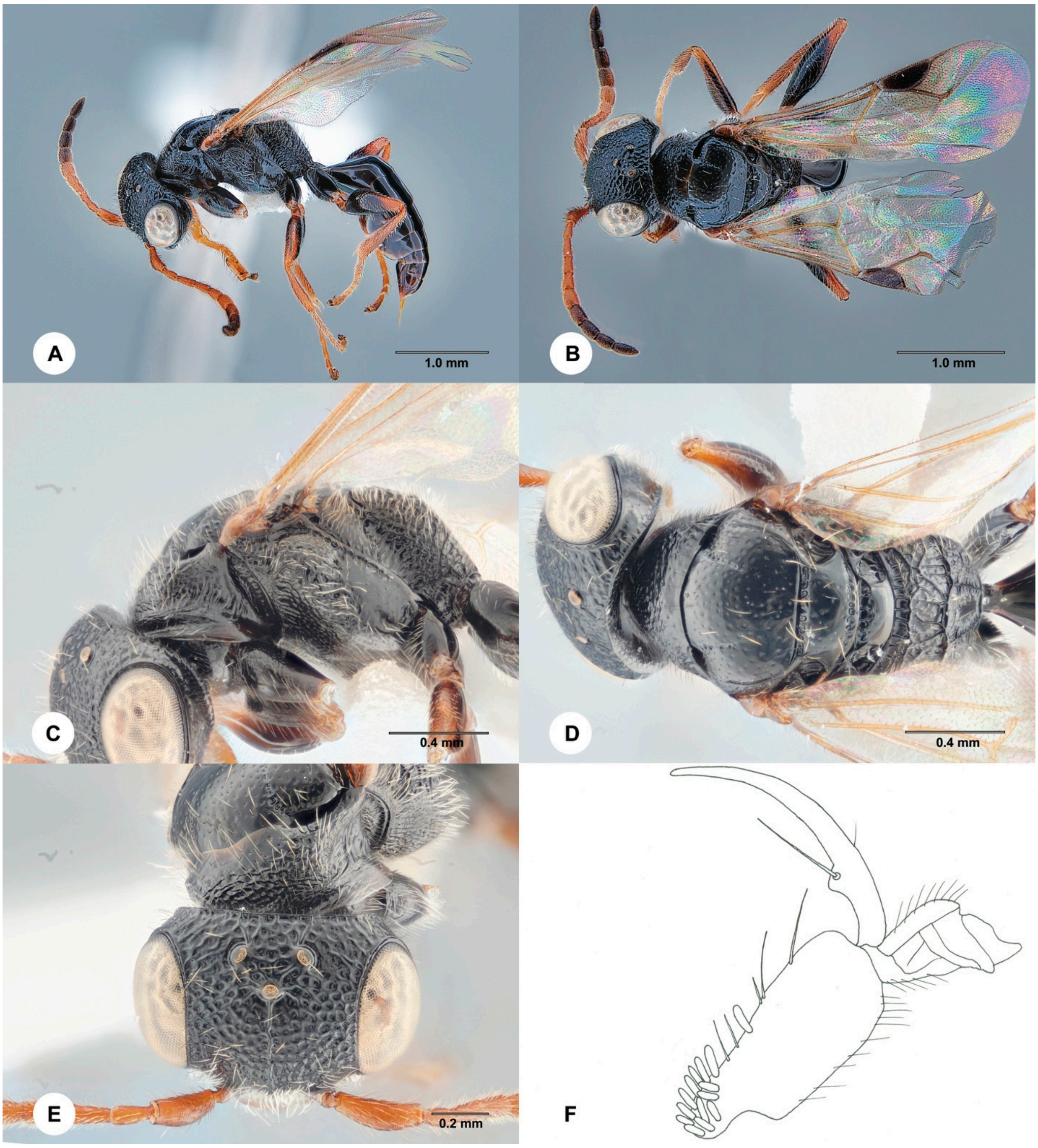

Figure S23. *Anteon pteromaculatum* Xu, Olmi, Guglielmino & Chen, 2012, female (SCAU 3040519) (**A**) Habitus, lateral view (**B**) Habitus, dorsal view (**C**) Head and mesosoma, lateral view (**D**) Head and mesosoma, dorsal view (**E**) Head, dorsal view (**F**) Chela.

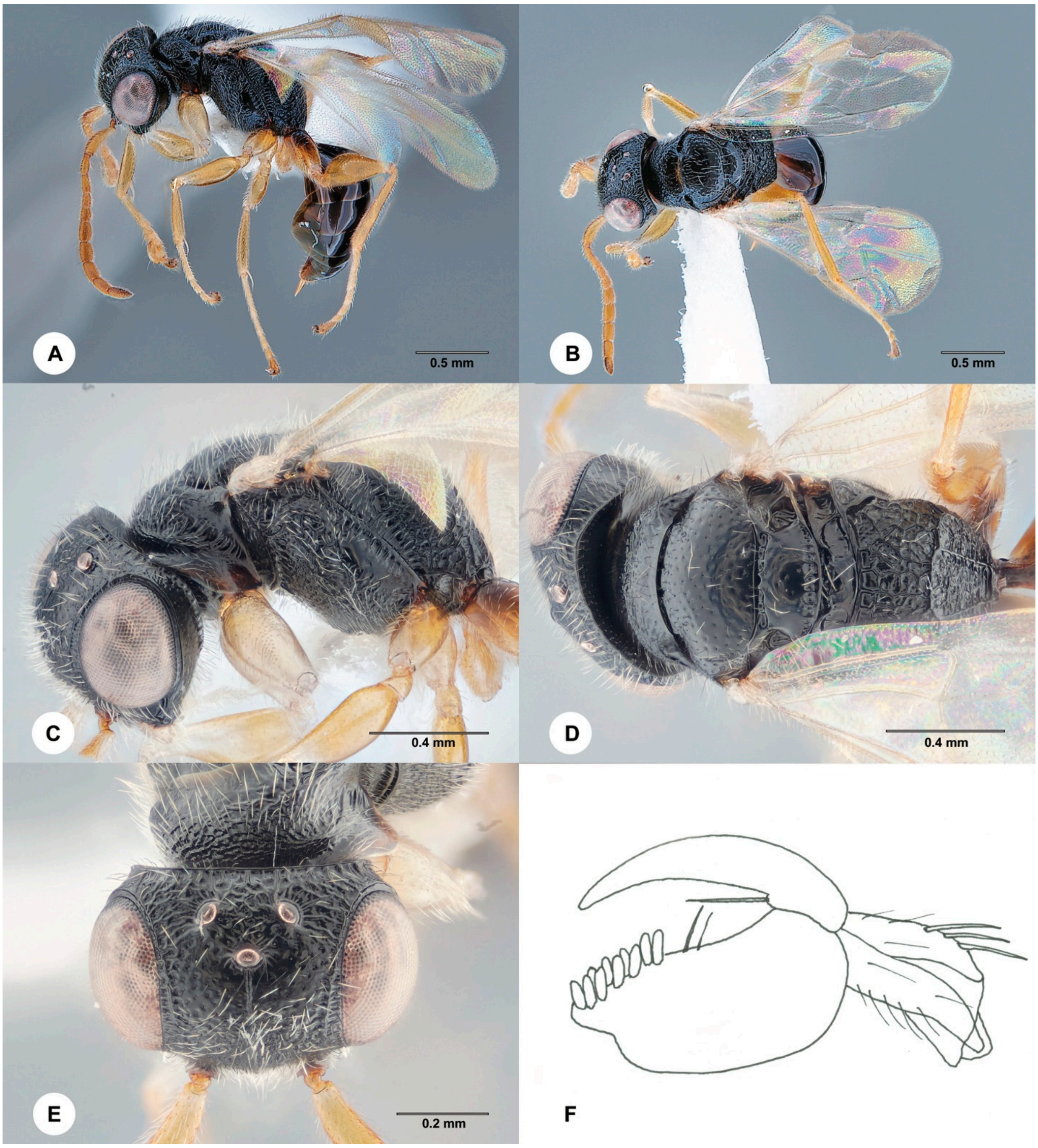

Figure S24. *Anteon tengchongense* Xu, He & Olmi, 1998, female (SCAU 3040523) (**A**) Habitus, lateral view (**B**) Habitus, dorsal view (**C**) Head and mesosoma, lateral view (**D**) Head and mesosoma, dorsal view (**E**) Head, dorsal view (**F**) Chela.

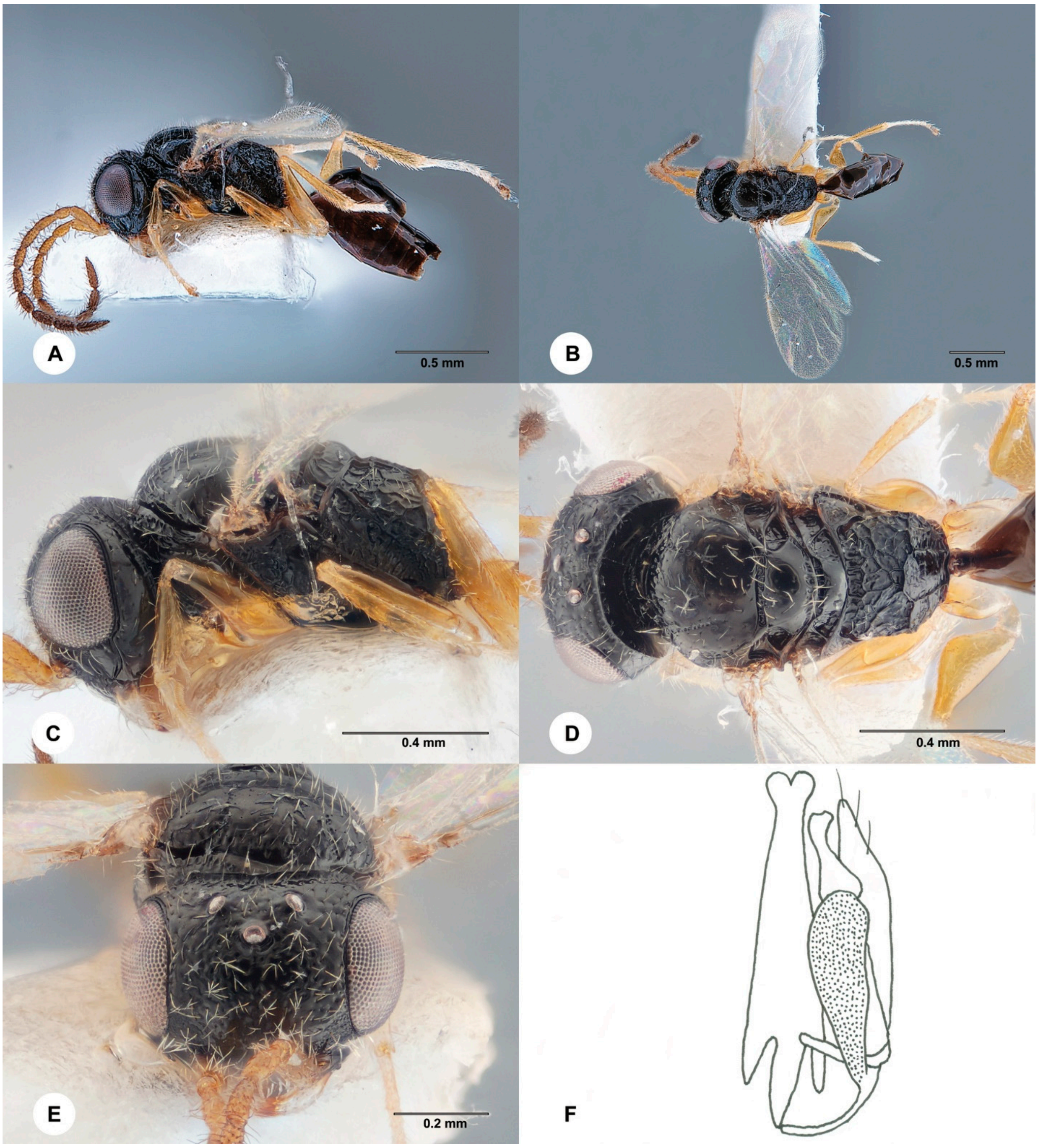

Figure S25. *Anteon yuani* Xu, He & Olmi 1998, male (SCAU 3011682) (**A**) Habitus, lateral view (**B**) Habitus, dorsal view (**C**) Head and mesosoma, lateral view (**D**) Head and mesosoma, dorsal view (**E**) Head, anterior view (**F**) Genitalia (left half removed).

Table S1. Details of sequenced specimens

Code	Figure No.	species	Sex	Collecting data
SCAU 3040430	Figure S1	*Anteon abdulnouri* Olmi, 1987	female	CHINA: Shandong, Linyi, Lanling County, 34°51'N, 118°4'E, 5-15.viii.2014, MT, Xuejun Yang
SCAU 3011714	Figure S2	*Anteon achterbergi* Olmi, 1991	female	CHINA: Shandong, Linyi, Lanling County, 34°51'N 118°4'E, 14-24.vii.2014, MT, Xuejun Yang
SCAU 3011677	Figure S3	*Anteon blanduscutum* Xu, He & Rui, 1996	female	CHINA: Yunnan, Xishuangbanna, Menghai, Bulangshan Village, 21°44.981′N, 100°26.907′E, 1683 m, Area A2, grass, 15.viii.2018, Li Ma
SCAU 3011678	Figure 2	*Anteon blanduscutum* Xu, He & Rui, 1996	male	CHINA: Yunnan, Xishuangbanna, Menghai, Bulangshan Village, 21°44.981′N, 100°26.907′E, 1683 m, Area A2, grass, 15.viii.2018, Li Ma
SCAU 3011669	Figure S4	*Anteon borneanum* Olmi, 1984	female	CHINA: Yunnan, Xishuangbanna, Menghai, Bulangshan Village, 21°44.745′N, 100°26.07′E, 1621m, Area D, near road, 20.vi-20.vii.2018, MT, Li Ma
SCAU 3011671	Figure 3	*Anteon clariclypeum* sp. n.	female	CHINA: Yunnan, Xishuangbanna, Menghai, Bulangshan Village, 21°44.761′N, 100°25.959′E, 1595 m, Area D, forest, MT, 16.viii-14.ix.2018, Li Ma
SCAU 3011712	Figure 5	*Anteon claricolle* Kieffer, 1906	male	CHINA: Inner Mongolia, Wulan Aodu Experimental Station, 38°46'15"N, 108°46'41"E, 20-30.vi.2013, MT, Yongming Luo
SCAU 3040561		*Anteon claricolle* Kieffer, 1906	female	CHINA: Beijing, Yuanqing County, Zhangshanying Town, 40.50847°N,115.78884°E, 807 m, 27.vi-4.vii.2012, MT, Jinyu Hu
SCAU 3040517	Figure S5	*Anteon confusum* Olmi, 1991	female	CHINA: Shaanxi, Yang County, Huayang Town, 33.382156°N, 107.507905°E, 1154m, 26.v-26.vi.2017, MT, Haoyu Liu
SCAU 3044058	Figure S6	*Anteon confusum* Olmi, 1991	male	CHINA: Yunnan, Xishuangbanna, Menghai, Bulangshan Village, 21°44.619′N, 100°26.721′E, 1692 m, Area C, forest, 28.v-28.vi.2019, MT, Li Ma
SCAU 3011621	Figure S7	*Anteon exiguum* (Haupt, 1941)	female	CHINA: Hebei, Xiaowutai National Nature Reserve, 39°52.048'N, 114°56.446'E, 1364 m, 13-20.viii.2012,MT, Haiming Zhang
SCAU 3040512	Figure S8	*Anteon fidum* Olmi, 1991	male	CHINA: Yunnan, Lanping Dist. Mt. Lasha, 26.324010°N, 99.275624°E, 2500 m, farm land, 20-30.vii.2018, MT, Jin-Ku Li
SCAU 3040522	Figure S9	*Anteon funiuense* Xu, He & Olmi, 2001	female	CHINA: Shaanxi, Yang County Yishui Town, 33.441897°N, 107.361713°E, 901 m, 24.v-24.vi.2017, MT, Haoyu Liu
SCAU 3044072	Figure S10	*Anteon henanense* Xu, He & Olmi, 2001	female	CHINA: Yunnan, Lanping Dist., Mt. Lasha, 26.324161°N, 99.256617°E, 2700 m, sample 564, 11-26.viii.2019, MT2, A. Reshchikov et al.
SCAU 3040511	Figure S11	*Anteon henanense* Xu, He & Olmi, 2001	male	CHINA:Yunnan, Lanping Dist. Mt. Lasha, 26.324010°N, 99.275624°E, 2500m, farm land, 20-30.vii.2018, MT, Jin-Ku Li
SCAU 3011713	Figure S12	*Anteon hilare* Olmi, 1984	female	CHINA: Shandong, Linyi, Lanling County, 34°51'N, 118°4'E, 14-24.vii.2014, MT, Xuejun Yang
SCAU 3011676	Figure S13	*Anteon hirashimai* Olmi, 1993	female	CHINA: Yunnan, Xishuangbanna, Menghai, Bulangshan Village, 21°44.619′N 100°26.721′E, 1692 m, Area C, forest, 21.vi-20.vii.2018, MT, Li Ma
SCAU 3011738	Figure 6	*Anteon maguanense* sp. n.	female	CHINA: Yunnan, Maguan Town, Muchang County, 22.91888°N, 104.162851°E, 1336 m, coniferous forest, vi.2017, MT, Li Ma
SCAU 3040514	Figure 7	*Anteon meifenganum* Olmi, 1991	female	CHINA: Shaanxi, Yang County Huayang Town, 33.382156°N, 107.507905°E, 1154 m, 26.v-26.vi.2017, MT, Haoyu Liu
SCAU 3040520	Figure 8	*Anteon meifenganum* Olmi, 1991	male	CHINA: Shaanxi, Yang County Huayang Town, 33.675537°N, 107.349632°E, 922 m, 12.iv-12.v.2017, MT, Haoyu Liu

SCAU 3040515	Figure S14	*Anteon mite* Olmi, 1996	male	CHINA: Shaanxi, Yang County Huayang Town, 33.382156°N, 107.507905°E, 1154 m, 26.v-26.vi.2017, MT, Haoyu Liu
SCAU 3011661	Figure S15	*Anteon multicolor* Xu, He & Olmi, 1998	female	CHINA: Yunnan, Xishuangbanna, Menghai, Bulangshan Village, 21°45.037´N, 100°26.715´E, 1683 m, Area A, grass, 17.v-21.vi.2018, MT, Li Ma
SCAU 3040571		*Anteon multicolor* Xu, He & Olmi, 1998	male	CHINA: Yunnan, Xishuangbanna, Menghai, Bulangshan Village, 21°44.521´N 100°26.647´E, 1646 m, Area C, grass, 17.v-21.vi.2018, MT, Li Ma
SCAU 3040573		*Anteon multicolor* Xu, He & Olmi, 1998	male	CHINA: Yunnan, Xishuangbanna, Menghai, Bulangshan Village, 21°44.521´N 100°26.647´E, 1646 m, Area C, grass, 17.v-21.vi.2018, MT, Li Ma
SCAU 3040574		*Anteon multicolor* Xu, He & Olmi, 1998	female	CHINA: Yunnan, Xishuangbanna, Menghai, Bulangshan Village, 21°44.521´N 100°26.647´E, 1646 m, Area C, grass, 17.v-21.vi.2018, MT, Li Ma
SCAU 3044011	Figure S16	*Anteon multicolor* Xu, He & Olmi, 1998	male	CHINA: Yunnan, Xishuangbanna, Menghai, Bulangshan Village, 21°44.521´N 100°26.647´E, 1646 m, Area C, grass, 28.vi-19.vii.2019, MT, Li Ma
SCAU 3044014		*Anteon multicolor* Xu, He & Olmi, 1998	female	CHINA: Yunnan, Xishuangbanna, Menghai, Bulangshan Village, 21°44.521´N 100°26.647´E, 1646 m, Area C, grass, 28.vi-19.vii.2019, MT, Li Ma
SCAU 3044065		*Anteon multicolor* Xu, He & Olmi, 1998	male	CHINA: Guangdong, Guangzhou, South China Botanical Garden, Pugang, 23°11’25.82’’N, 113°21’47.01’’E, 7.vii-7.viii.2019， MT7, Shixiao Luo
SCAU 3044074		*Anteon multicolor* Xu, He & Olmi, 1998	female	CHINA: Yunnan, Lanping Dist., Mt. Lasha, 26.324161°N, 99.256617°E, 2700 m, sample 564 11-26.viii.2019, MT2, A. Reshchikov et al.
SCAU 3044114		*Anteon multicolor* Xu, He & Olmi, 1998	male	CHINA:Yunnan, Lanping Dist. Mt. Lasha, 26.324161°N, 99.256617°E, 2700m, boskage, 10-20.vii.2018, MT, Jin-Ku Li
SCAU 3048997		*Anteon multicolor* Xu, He & Olmi, 1998	male	CHINA: Yunnan, Mt. Gaoligong, 25°23'59.6"N, 98°42'26.6"E, 1951m, 1-15.vi.2020, MT, Yi Lang
SCAU 3040524	Figure S17	*Anteon munitum* Olmi, 1984	male	CHINA: Shaanxi, Yang County, Yishui Town, 33.441897°N, 107.361713°E, 901 m, MT, 24.v-24.vi.2017, MT, Haoyu Liu
SCAU 3040521	Figure S18	*Anteon naduense* Olmi 1987	female	CHINA: Shaanxi, Yang County, Yishui Town, 33.441897°N, 107.361713°E, 901 m, MT, 24.v-24.vi.2017, MT, Haoyu Liu
SCAU 3040433	Figure S19	*Anteon nanlingense* Xu, Olmi & He, 2011	female	THAILAND: Mae Hong Son Prov., Pai, 19°22’4.4”N, 98°30’16”E, 745m, 1.ii-26.iii.2016, MT, A. Reshchikov
SCAU 3048821	Figure S20	*Anteon nanlingense* Xu, Olmi & He, 2011	male	CHINA: Guangdong, Shenzhen, Huaqiaocheng, wet land, 22.533217°N, 113.968771°E, 31.iii-29.iv.2020, MT, Longlong Chen
SCAU 3011658	Figure 9	*Anteon parafidum* sp. n.	female	CHINA: Yunnan, Xishuangbanna, Menghai, Bulangshan Village, 21°45.037´N, 100°26.715´E, 1683 m, Area A, grass, 17.v-21.vi.2018, MT, Li Ma
SCAU 3011662	Figure 10	*Anteon parafidum* sp. n.	male	CHINA: Yunnan, Xishuangbanna, Menghai, Bulangshan Village, 21°45.037´N, 100°26.715´E, 1683 m, Area A, grass, 17.v-21.vi.2018, MT, Li Ma
SCAU 3040566		*Anteon parafidum* sp. n.	female	CHINA: Yunnan, Xishuangbanna, Menghai, Bulangshan Village, 21°44.981´N 100°26.907´E, 1683 m, Area A2, grass, 15.viii.2018, MT, Li Ma
SCAU 3040567		*Anteon parafidum* sp. n.	male	CHINA: Yunnan, Xishuangbanna, Menghai, Bulangshan Village, 21°44.981´N 100°26.907´E, 1683 m, Area A2, grass, 15.viii.2018, MT, Li Ma
SCAU 3044018		*Anteon parafidum* sp. n.	male	CHINA: Yunnan, Xishuangbanna, Menghai, Bulangshan Village, 21°45.037´N, 100°26.715´E, 1683 m, Area A1, grass, 21.viii-20.ix.2019, MT, Li Ma

SCAU 3044026		*Anteon parafidum* sp. n.	female	CHINA: Yunnan, Xishuangbanna, Menghai, Bulangshan Village, 21°45.037′N, 100°26.715′E, 1683 m, Area A1, grass, 21.viii-20.ix.2019, MT, Li Ma
SCAU 3044033		*Anteon parafidum* sp. n.	female	CHINA: Yunnan, Xishuangbanna, Menghai, Bulangshan Village, 21°44.981′N, 100°26.907′E, 1683 m, Area A2, grass, 20.iv-28.v.2019, MT, Li Ma
SCAU 3044066		*Anteon parafidum* sp. n.	male	CHINA: Yunnan, Xishuangbanna, Menghai, Bulangshan Village, 21°45.037′N, 100°26.715′E, 1683 m, Area A1, grass, 28.v-28.vi.2019, MT, Li Ma
SCAU 3044088		*Anteon parafidum* sp. n.	male	CHINA: Yunnan, Xishuangbanna, Menghai, Bulangshan Village, 21°45.037′N, 100°26.715′E, 1683 m, Area A1, grass, 20.vii.-21.viii.2019, MT, Li Ma
SCAU 3044089		*Anteon parafidum* sp. n.	female	CHINA: Yunnan, Xishuangbanna, Menghai, Bulangshan Village, 21°45.037′N, 100°26.715′E, 1683 m, Area A1, grass, 20.vii.-21.viii.2019, MT, Li Ma
SCAU 3040518	Figure S21	*Anteon pilosum* Xu, Olmi & He, 2010	male	CHINA: Shaanxi, Yang County, Huayang Town, 33.382156°N, 107.507905°E, 1154 m, 26.v-26.vi.2017, MT, Haoyu Liu
SCAU 3011631	Figure 11	*Anteon priscum* Olmi, 1991	female	CHINA: Jiangsu, Nanjing, Xianlin, Mt. Duoshan, 32°6'51"N, 118°54'43"E, 30.iv-7.v.2012, MT, Jie Zhao
SCAU 3011723	Figure S22	*Anteon priscum* Olmi, 1991	male	CHINA: Jiangsu, Nanjing, Xianlin, Mt. Duoshan, 32°6'51"N 118°54'43"E, 9-15.iv.2012, MT, Jie Zhao
SCAU 3040519	Figure S23	*Anteon pteromaculatum* Xu, Olmi, Guglielmino & Chen, 2012	female	CHINA: Shaanxi, Yang County, Huayang Town, 33.675537°N, 107.349632°E, 922 m, 12.iv-12.v.2017, MT, Haoyu Liu
SCAU 3040516	Figure 12	*Anteon shaanxianum* sp. n.	male	CHINA: Shaanxi, Yang County, Huayang Town, 33.382156°N, 107.507905°E, 1154 m, 26.v-26.vi.2017, MT, Haoyu Liu
SCAU 3011688	Figure 13	*Anteon shandonganum* sp. n.	female	CHINA: Shandong, Shanghe County, 37°16′4′′N, 117°9′10′′E, 23-29.vi.2018, MT3, Jiahe Yan
SCAU 3040523	Figure S24	*Anteon tengchongense* Xu, He & Olmi, 1998	female	CHINA: Shaanxi, Yang County, Yishui Town, 33.441897°N 107.361713°E, 901 m, 24.v-24.vi.2017, MT, Haoyu Liu
SCAU 3011682	Figure S25	*Anteon yuani* Xu, He & Olmi, 1998	male	CHINA: Guangdong, Guangzhou, University Town, 23°2′55"N 113°23′12"E, forest, 16-26.x.2018, MT, Huayan Chen

Table S2. Genetic distance of *COI* within *Anteon* species under K2P model

Species	Distance(%)
Anteon abdulnouri	NA
Anteon achterbergi	NA
Anteon blanduscutum	1.6
Anteon borneanum	NA
Anteon clariclypeum	NA
Anteon claricolle	0.8–1.8
Anteon collare	NA
Anteon confusum	2
Anteon ephippiger	NA
Anteon exiguum	1.4
Anteon fulviventre	NA
Anteon fidum	NA
Anteon funiuense	NA
Anteon gaullei	NA
Anteon henanense	0
Anteon hilare	NA
Anteon hirashimai	NA
Anteon maguanense	NA
Anteon meifenganum	0
Anteon mite	NA
Anteon multicolor	0.8–0.5
Anteon munitum	NA
Anteon naduense	NA
Anteon nanlingense	3
Anteon parafidum	0.8–0.3
Anteon pilosum	NA

Anteon priscum	0.6
Anteon pteromaculatum	NA
Anteon shaanxianum	NA
Anteon shandonganum	NA
Anteon tengchongense	NA
Anteon yuani	NA

Table S3. Interspecific pairwise distance of *Anteon* based on COI sequences (%)

		1	2	3	4	5	6	7	8	9	10	11	12	13	14	15	16	17	18	19	20	21	22	23	24	25	26	27	28	29	30	31	32
1	*Anteon abdulnouri*		5.7	19.4–19.9	15.5	20	15.5–16.1	16.1	18–18.8	16.9	15.7–15.8	18.5	16.8	16.2	18.5	18.8–18.9	15.7	16.5	20.7	16.6–16.7	15.8	17–17.5	17.6	17.8	15.7–16.5	15.8–16.4	19.6	16.1–16.8	18.2	17.1	14	15.5	15
2	*Anteon achterbergi*			16.1–16.5	15.2	16.5	3.1–4.6	9.1	12.1–12.6	9.4	11–11.3	10.8	12.7	13.8	12.3	17.1–17.2	17.7	15.2	17.9	14.5–14.6	15.2	9.6–10.1	13.3	13.3	14.1–14.3	11.4–12.1	17.7	11–11.9	15.3	10.4	8.8	4.6	12.1
3	*Anteon blanduscutum*				17.8–18	19.4–19.8	17.2–18.1	16.7–17	16.7–17.6	17–17.2	17.3–18.1	17–17.5	17.8–18.1	13.1–13.2	18.3–18.8	19.2–20.2	20.7–21.1	17.9–18.5	17.6–17.7	18.9–19.3	17.6–18	16.4–17.8	17.8–18.2	19.3–19.8	19–19.6	16.8–17.7	19.6–19.8	15.2–16.4	19.8–19.9	15.8–16.3	16.5–16.7	17.4–17.6	14.9–15.3
4	*Anteon borneanum*					17.1	16.4–17	14.9	14.6–14.8	15.7	15.9–16.1	16.8	14.7	15.7	17.7	16.4–16.8	16.4	14.7	19.9	14.5–14.6	15.5	16.5–16.9	15.7	18.6	16.1–16.4	14.6–15.4	15.8	15.7–16.4	15.2	15.2	16.3	15.1	13.7
5	*Anteon clariclypeum*						16.8–17.7	15.2	16.9–17.6	17.2	17.3–18	17.5	16.3	15.5	18.1	19.4–19.6	19.3	18.9	18.5	16.7–16.8	17.9	17–17.2	15.4	17.3	17.1–17.3	15.9–16.7	18.9	17.3–18	19.1	16.9	16.4	16.4	15.3
6	*Anteon claricolle*							10.3–10.4	13.7–14.7	9.8–10	11.2–11.7	12.3–12.5	13.2–13.4	13.9–14.5	12.5–12.9	16.9–17.2	17.7–18.1	16.7	19.1–19.3	15.5–16.1	16–16.3	10.2–11.4	14.5–15.1	13.6–15	14.6–15.5	11.6–12.5	18.3–18.4	11.9–13.1	16–17	11.3–11.5	10.2–10.5	15.4–15.8	12.6–13.1
7	*Anteon collare*								13.5–14.1	4.1	11.1	8.7	11	12.3	9.1	17.8–18.1	17.4	15.4	18.3	15.2	13.9	10–10.2	13.4	13.4	13.4–13.7	11.1–11.6	16.4	10.4–11.1	15.6	10.2	8.7	4.3	10.8
8	*Anteon confusum*									14.5–14.7	15.1–15.7	14.6–14.7	15.3	13.1–13.7	5.6–6.3	18.3–18.9	16.3–16.8	15.7–15.8	17.7–18.6	16.6–17	16.2	13.3–14.6	13.5–14.3	17.1–17.4	12.4–13.1	14.8–15.1	17.9–18.1	12.8–13.9	14.6–15.3	13.2–14.1	13.7–14.8	14.5–14.7	14.3
9	*Anteon ephippiger*										11.1–11.4	8.5	11.6	13.1	9.9	18.1–18.4	18.3	16.4	19	16.4	15.2	10.7–15.2	15.2	14.3	14.2–14.3	11.6–12.4	16.9	10.9–11.8	15.4	11	9.7	15.2	11.7
10	*Anteon exiguum*											13.2	7.5	14.3–14.5	14.2–14.5	19.4–20.1	16.8–16.9	16.6–16.7	18–18.4	15.5–15.8	15.1	11.7–12.6	15.4	14.5–14.8	12.6–13.7	7.1–7.7	17.3–17.7	11.8–12.6	16.8	12.9–13.2	11.1–11.4	14.9–15.8	11.9–12.2
11	*Anteon fulviventre*												14.3	14.2	12.3	18.6–18.7	18.9	17.2	18.4	15.7	17.2	10.5–10.7	14.3	14.7	16.9–17.2	14.2–14.6	17.5	13.4–14.2	16.2	11.6	11.1	14.2	12.9
12	*Anteon fidum*													12.9	15	18.4–18.9	15.9	16.8	18.5	15	13.4	12.3–13	14.2	14.9	13.2–13.3	3.8–4	18	11.7–12.1	15.6	12.8	11.1	14.9	10.4
13	*Anteon funiuense*														15.1	17–17.2	16.7	16	17.3	13.9–14	14	14.2–14.6	13.9	15.6	11.7–11.8	12.1–12.7	17.6	13.2–13.8	14.8	13.2	13.5	13.2	12.9
14	*Anteon gaullei*															19.9–20.4	18.7	18	21	19	17.7	12–12.6	14.9	15.3	16.4–17.2	14.1–14.9	17.2	13.3–14.1	18.3	12.8	11.9	16	13.7
15	*Anteon henanense*																18.9–19.4	18.6–18.8	19.9–20	17.4	17.9–18.6	17–18.1	7.9–8.3	20.6–20.7	17–17.6	17.1–17.9	17.2–17.3	18.2–18.6	18.4–18.6	16.3–16.4	17.6–17.7	15.5–15.9	17.1–17.7
16	*Anteon hilare*																	16.5	20.5	18.7–18.8	17	17.7–18.2	17.5	19.2	15.9	15.5–16	18	16.7–17.7	17.6	15.8	16.6	16.3	14.7
17	*Anteon hirashimai*																		20	14.6–14.7	13.5	16.5–17.1	16.4	18.3	16.1	16.7–17.1	16.4	16–17.3	15.9	15.4	16.6	14.9	14.6
18	*Anteon maguanense*																			18.3	20.2	18.6–19.1	18.8	19.6	17.4–17.7	17.3–17.9	20.4	16.6–17.6	20.5	18.6	18.5	18.9	18.2
19	*Anteon meifenganum*																				14.7–14.8	15.8–16.3	15.4–15.5	17.5–17.6	14.5–14.8	13.8–14.7	15.6–15.7	15.5–16.2	11.1	16.3	16–16.1	14.6	15.5–15.6
20	*Anteon mite*																					16–17.1	15.5	16.7	13–13.6	13.1–13.5	15	14.5–14.9	15.4	15.2	14.6	14.9	13
21	*Anteon multicolor*																						14.2–14.5	13.7–14.5	15.9–16.4	11.6–12.7	17–17.4	11.4–12.9	14.7–15.1	10.7–11.2	08.7–9	15.6–16.2	12–12.8
22	*Anteon munitum*																							15.3	13.9–14.2	12.7–13.1	15.8	12.9–13.6	17.3	14.3	12.4	15.6	13.2
23	*Anteon naduense*																								16.7–17.1	14.1–14.4	19	15.3–15.8	19.4	14.5	13.6	17.8	14
24	*Anteon nanlingense*																									12.5–13.7	18–18.5	14–14.5	14.5–15.5	14.9–15.1	14.1–14.3	14.9–15.1	13.2–13.5
25	*Anteon parafidum*																										17.9–18.7	20.5–11.3	14.1–14.9	11.6–12.4	10.9–11.3	15.2–15.8	9.4–10.1
26	*Anteon pilosum*																											15.2–15.7	17.8	16.9	17.4	15.9	15.9
27	*Anteon priscum*																												14.4–15.1	13.8–14.5	11.5–12.5	15.1–15.7	10.3–11.3
28	*Anteon pteromaculatum*																													15.9	16.2	15.7	15.2
29	*Anteon shaanxianum*																														11	14.3	12.3
30	*Anteon shandonganum*																															15.2	12.3
31	*Anteon tengchongense*																																15.1
32	*Anteon yuani*																																
